# Comparative genomics of pyridoxal 5′-phosphate-dependent transcription factor regulons in *Bacteria*

**DOI:** 10.1099/mgen.0.000047

**Published:** 2016-01-18

**Authors:** Inna A. Suvorova, Dmitry A. Rodionov

**Affiliations:** ^1^​ A. A. Kharkevich Institute for Information Transmission Problems, Russian Academy of Science, Moscow, Russia; ^2^​ Sanford-Burnham-Prebys Medical Discovery Institute, La Jolla, CA 92037, USA

**Keywords:** bacteria, comparative genomics, DNA motif, regulon reconstruction, transcription factor

## Abstract

The MocR-subfamily transcription factors (MocR-TFs) characterized by the GntR-family DNA-binding domain and aminotransferase-like sensory domain are broadly distributed among certain lineages of *Bacteria*. Characterized MocR-TFs bind pyridoxal 5′-phosphate (PLP) and control transcription of genes involved in PLP, gamma aminobutyric acid (GABA) and taurine metabolism via binding specific DNA operator sites. To identify putative target genes and DNA binding motifs of MocR-TFs, we performed comparative genomics analysis of over 250 bacterial genomes. The reconstructed regulons for 825 MocR-TFs comprise structural genes from over 200 protein families involved in diverse biological processes. Using the genome context and metabolic subsystem analysis we tentatively assigned functional roles for 38 out of 86 orthologous groups of studied regulators. Most of these MocR-TF regulons are involved in PLP metabolism, as well as utilization of GABA, taurine and ectoine. The remaining studied MocR-TF regulators presumably control genes encoding enzymes involved in reduction/oxidation processes, various transporters and PLP-dependent enzymes, for example aminotransferases. Predicted DNA binding motifs of MocR-TFs are generally similar in each orthologous group and are characterized by two to four repeated sequences. Identified motifs were classified according to their structures. Motifs with direct and/or inverted repeat symmetry constitute the majority of inferred DNA motifs, suggesting preferable TF dimerization in head-to-tail or head-to-head configuration. The obtained genomic collection of *in silico* reconstructed MocR-TF motifs and regulons in *Bacteria* provides a basis for future experimental characterization of molecular mechanisms for various regulators in this family.

## Data Summary

Inferred transcription factor binding sites and reconstructed MocR-subfamily regulons have been deposited in the RegPrecise database (http://regprecise.lbl.gov/RegPrecise/collection_tffam.jsp?tffamily_id = 83).

## Impact Statement

Bacteria in most ecological niches are constantly exposed to variations in many factors including nutrient availability. Changes in gene expression using transcription factors allow bacteria to adapt to these variations. Regulators from the MocR subfamily of the GntR family are broadly distributed in *Bacteria*. As most of these regulators remain uncharacterized, we utilized a comparative genomics approach to predict target DNA binding sites and regulated genes for a large portion of MocR-like regulators in near three hundreds of bacterial genomes. The inferred DNA binding motifs were classified based on their sequence and structure for the first time here. The reconstructed regulon contents suggest numerous novel functional associations between both known and uncharacterized genes encoding enzymes and transporters involved in the metabolisms of pyridoxal 5′-phosphate, gamma aminobutyric acid, ectoine and taurine, thus providing testable hypotheses for future experimental studies. This study demonstrates the power of comparative genomics for the reconstruction of transcription factor regulons in bacteria. The inferred collection of reconstructed regulons can be used in genetic experiments, metabolic modelling and evolutionary analysis.

## Introduction

Transcription factors (TFs) are proteins that recognize specific *cis*-regulatory DNA sequences (transcription factor binding sites, TFBSs) to either stimulate or repress transcription of genes. The GntR family is a large and widespread group of bacterial TFs that regulate various biological processes including utilization of sugars and other carbon sources, and amino acid and fatty acid metabolism (Haydon & Guest, 1991; Rigali *et al.*, 2002). TFs from the GntR family (GntR-TFs) contain a conserved N-terminal DNA-binding domain with the helix–turn–helix (HTH) motif, but differ in their C-terminal effector-binding and oligomerization domains. Depending on the structure of a C-terminal domain, GntR-TFs are classified into six subfamilies (Rigali *et al.*, 2002, 2004).

The MocR subfamily was named after the putative rhizopine utilization regulator MocR in *Sinorhizobium meliloti* (Rossbach *et al.*, 1994). It was also called MocR/GabR, after the gamma aminobutyric acid (GABA) utilization regulator GabR, the first experimentally characterized TF from this subfamily (Belitsky & Sonenshein, 2002). Regulators from the MocR subfamily (MocR-TFs) have a large C-terminal domain of ∼300–350 aa homologous to the class I aminotransferases, fused to a GntR-family DNA-binding domain (Rigali *et al.*, 2002; Bramucci *et al.*, 2011; Milano *et al.*, 2015). The class I aminotransferases catalyse transamination of amino acids to α-keto acids and use pyridoxal 5′-phosphate (PLP) as a cofactor. Similar requirement for PLP was shown *in vivo* and *in vitro* for several characterized MocR-TFs including GabR, TauR and various PdxRs (see below). Moreover, PdxR proteins are directly involved in the regulation of PLP metabolism.

PLP is a biologically active form of vitamin B6, which exists in the form of six interconvertible vitamers: pyridoxine, pyridoxamine, pyridoxal and their 5′-phosphorylated forms. Bacteria can synthesize PLP *de novo* using either the PdxS/PdxT or the PdxA/PdxJ pathway (El Qaidi *et al.*, 2013), or via the salvage pathway, where vitamin B6 vitamers are phosphorylated by kinases from the PdxK or ThiD families (Park *et al.*, 2004, Newman *et al.*, 2006). Pyridoxine 5′-phosphate (and, less preferably, pyridoxamine 5′-phosphate) can be oxidized to PLP via PdxH (di Salvo *et al.*, 2003). PLP is an essential cofactor for numerous enzymes in prokaryotes and eukaryotes, the majority of which are involved in amino acid metabolism (Percudani & Peracchi, 2003). In free-living prokaryotes, almost 1.5 % of genes generally encode PLP-dependent enzymes. The most common enzymes requiring PLP are aminotransferases, amino acid racemases, decarboxylases, glycogen phosphorylases, as well as enzymes catalysing β- or γ-elimination or replacement. Moreover, PLP is involved in stress responses, particularly to oxidative stress, quenching reactive oxygen species (Bilski *et al*., 2000). Thus, dysregulation of PLP metabolism can cause various defects, such as growth inhibition, lowered stress tolerance, reduced glucan production and biofilm formation (Liao *et al*., 2015).

The few MocR-TFs characterized so far include several activators of PLP biosynthesis or salvage genes in Corynebacterium glutamicum, Listeria monocytogenes, Bacillus clausii and two Streptococcus species (Streptococcus mutans and Streptococcus pneumoniae) (Jochmann *et al*., 2011; El Qaidi *et al*., 2013; Belitsky, 2014; Liao *et al*., 2015; Tramonti *et al.*, 2015), GabR from *Bacillus subtilis*, an activator of GABA utilization (Belitsky & Sonenshein, 2002; Belitsky, 2004), and TauR in various bacteria (e.g. *Sinorhizobiummeliloti*, *Rhodobacter capsulatus*), which upregulates taurine dissimilation (Wiethaus *et al.*, 2008; Mostafavi *et al.*, 2014).

The MocR-TFs are often encoded by genes that are divergently transcribed with their positively regulated target genes (forming so-called divergons), and also often act as auto-repressors. It was shown that apo-GabR binds to DNA and represses expression from its own promoters, but requires the presence of PLP and GABA to activate expression of the structural genes (Belitsky & Sonenshein, 2002, Belitsky, 2004). In contrast, the apo-PdxR regulators from *B. clausii*, *C. glutamicum*, *L. monocytogenes* and *Streptococcuspneumonia* bind their DNA motifs in the common intergenic region of *pdxST* and *pdxR* to activate the former operon and repress the latter gene, while PLP acts as an anti-activator of PdxR (Jochmann *et al.*, 2011; El Qaidi *et al.*, 2013; Belitsky, 2014; Tramonti *et al.*, 2015).

It was proposed that, similarly to class I aminotransferases, MocR-TFs form head-to-tail dimers (Rigali *et al.*, 2002). Indeed, the obtained crystal structure of GabR confirmed the head-to-tail dimer arrangement (Edayathumangalam *et al.*, 2013). GntR-TFs generally bind as dimers to inverted repeats/palindromic DNA operator sequences so that each monomer recognizes a half-site, but it was proposed that the head-to-tail configuration is not appropriate to binding inverted repeats, although it is well adapted to binding direct repeats sufficiently spaced to form DNA looping (Rigali *et al.*, 2002). Therefore, it is likely that DNA operators of MocR-TFs would often include direct repeats. In agreement with this hypothesis, many experimentally characterized MocR-TFs bind either direct DNA repeats [such as GabR in *B. subtilis* (Belitsky, 2004) and TauR in *Rhodobacter capsulatus* (Wiethaus *et al.*, 2008)], or mixed direct and inverted repeats [PdxR in *B. clausii* (Tramonti *et al.*, 2015) and *C. glutamicum* (Jochmann *et al.*, 2011)]. Notably, directs repeats in these motifs are separated by either two or three DNA helical turns, so that they are located on the same side of the DNA. Some MocR-TFs bind inverted repeats as well [PdxR in *L. monocytogenes* (Belitsky, 2014)]. It was proposed that PLP binding might greatly change TF conformation (which is possible due to the flexible linker region between the domains of MocR-TFs) and consequently its specificity towards the DNA motif structure (Tramonti *et al.*, 2015). Apo-PdxR is able to bind both direct and inverted repeats, favouring the former, while PdxR in complex with PLP has much lower affinity for direct repeats and favours binding to inverted repeats.

Genes encoding putative MocR-TFs are widespread in the genomes of various bacteria, being often present as multiple paralogues, although most of these regulators remain uncharacterized (Bramucci *et al.*, 2011; Belitsky, 2014). *In silico* identification of DNA-binding sites and comparative genomics-based reconstruction of putative TF regulons (sets of co-regulated genes) is a powerful approach to initially map potential functions of multiple diverse representatives in large TF families. We previously used this bioinformatics approach for reconstruction of reference sets of regulons in various groups of bacterial genomes including the *Bacillales*, *Lactobacillales*, *Staphylococcus* and *Shewanella* species (Ravcheev *et al.*, 2011, 2013a; Rodionov *et al.*, 2011; Leyn *et al.*, 2013). In particular, we have recently analysed two large collections of TFs from the LacI family (Ravcheev *et al.*, 2014), and from the FadR, HutC and YtrA subfamilies of the GntR family (Suvorova *et al.*, 2015).

In this study, we utilized the comparative genomics approach to identify putative DNA binding motifs and reconstruct transcriptional regulons for MocR-TFs in a non-redundant set of 390 genomes that represent species from 43 diverse lineages of *Bacteria*. As result, we report identification of putative regulons for 825 TFs classified into 86 orthologous groups. Metabolic reconstructions revealed that most of the reconstructed regulons are involved in the metabolism of PLP, GABA, ectoine and taurine, while other MocR-TFs control genes encoding various enzymes involved in reduction/oxidation processes, putative metabolite transporters and PLP-dependent enzymes. By combining the functional annotations with phylogenetic analysis, we describe functional diversity for the MocR-TF regulons.

## Methods

Homologues of MocR-TFs were identified by psi-blast (Altschul *et al.*, 1997) (default E-value cut-off, e^− 20^), and orthologues were identified by reconstruction of phylogenetic trees for identified homologues, and by analysis of gene neighbourhoods (e.g. co-localization with the same genes of a certain metabolic pathway in related genomes) using MicrobesOnline (Dehal *et al.*, 2010). In most cases, an orthologous group contained one TF per genome. However, in some cases, several closely related TFs from the same genome (resulting from recent duplications or close-range horizontal transfers) were assigned to the same orthologous group.

For each identified group of orthologous MocR-TFs, we performed comparative genomics-based identification of their putative TFBS motifs followed by regulon reconstruction. Candidate TFBSs were identified by phylogenetic footprinting and confirmed by the consistency check approach (Rodionov, 2007). These bioinformatics techniques were previously used for identification of TFBSs in poorly studied groups of bacteria such as *Bacteroides* and *Thermotoga* (Ravcheev *et al.*, 2013b; Rodionov *et al.*, 2013). We analysed multiple alignments of the upstream regions of genes potentially regulated by orthologous MocR-TFs and identified groups of conserved positions with inverted or direct repeat symmetry. Initial candidate genes were those encoding TFs, as they are often auto-regulated, and nearby genes, as genes of the TFs and the genes they regulate are usually co-localized in the genome (Tan *et al.*, 2005).

For each candidate TFBS motif, a specific nucleotide position weight matrix (PWM, or a profile) was constructed by SignalX (as previously described by Gelfand *et al.*, 2000) and RegPredict (Novichkov *et al.*, 2010) using training sets of upstream regions of genes presumably belonging to the corresponding regulon. The constructed profiles were used to search for additional regulon members in the analysed genomes possessing a MocR-TF orthologue. The PWM-based searches for TFBSs in upstream gene regions (usually from − 350 to +50 nt relative to the translation start) were performed using GenomeExplorer (Mironov *et al.*, 2000) and RegPredict. The TFBS score thresholds for site searches were usually selected 10 % below the lowest site score in the training set. Weaker sites (with scores 10 % less than the threshold) were also taken into account if their positions were similar to positions of stronger sites upstream of orthologous genes and there were no stronger competing sites in the same intergenic region. New candidate members were attributed to the regulon if they were preceded by candidate binding sites in several genomes, the exact number of genomes depending on the number of genomes in a taxonomic group. The reconstructed regulons were extended to include all genes in putative operons. Genes were considered to belong to an operon if they were transcribed in the same direction, with intergenic distances not exceeding 200 nt, and when such organization persisted in several genomes. All reconstructed regulons including TFs, TFBSs and TF-regulated genes were captured and displayed in RegPrecise (Novichkov *et al.*, 2013), a specialized database of bacterial regulons (Data Citation 1).

The selected bacterial genomes were downloaded from GenBank (Benson *et al.*, 1999). Biological functions of predicted target genes were assigned using genomic context-based methods and the UniProt (Magrane & UniProt Consortium, 2011), MicrobesOnline (Dehal *et al.*, 2010) and Pfam (Finn *et al.*, 2014) databases. Multiple amino acid and nucleotide sequence alignments were built by muscle (Edgar, 2004). Phylogenetic trees were reconstructed with the phylip package using the maximum-likelihood method for tree reconstruction and the protdist program for distance calculation (Felsenstein, 1996). Motif logos were drawn with WebLogo (Crooks *et al.*, 2004).

## Results and Discussion

### Statistics of reconstructed regulons and regulogs

For the comparative analysis of MocR-TF regulons, we selected a set of 390 representative genomes from 43 diverse taxonomic groups of *Bacteria* that excludes closely related strains and species and which is most suitable for the comparative genomics-based regulon reconstruction (Table S1a, available in the online Supplementary Material). Among the analysed lineages that possess at least one MocR-TF, there are 22 taxonomic groups of *Proteobacteria*, ten groups of *Firmicutes*, six groups of *Actinobacteria*, three lineages of *Bacteroidetes*, as well as the *Cyanobacteria* and *Deinococcus*/*Thermus* groups (Table 1). MocR-TFs are widely distributed among *Proteobacteria* (∼73 % of the studied TFs), as well as *Actinobacteria* (∼13 %) and *Firmicutes* (∼11 %). The percentage of analysed genomes that contain MocR-TFs and the mean number of MocR-TFs per genome varied in different taxonomic groups (Table 1), and general trends observed in this work correspond well to the previously described data (Bramucci *et al.*, 2011). Thus, the genomes of betaproteobacteria encode the highest number of MocR-TFs.

**Table 1.** Taxonomic distribution of studied MocR-subfamily TFs in *Bacteria*

Taxonomic group	Lineages	Genomes*	Regulogs	Regulons	TFs†
*Alphaproteobacteria*	5	37 (50)	42	101	105 (2.84)
*Betaproteobacteria*	5	36 (40)	82	242	268 (7.44)
*Gammaproteobacteria*	10	67 (90)	81	203	208 (3.10)
*Deltaproteobacteria*	2	14 (19)	7	19	19 (1.36)
Total for *Proteobacteria*:	22	154 (199)	212	565	600 (3.89)
*Firmicutes* (*Bacilli*)	6	35 (54)	22	60	64 (1.83)
*Firmicutes* (*Clostridia*)	4	21 (32)	9	27	29 (1.38)
*Actinobacteria*	6	35 (48)	31	87	106 (3.03)
*Bacteroidetes* (*Bacteroidaceae*, *Flavobacteria*, *Sphingobacteria*)	3	11 (38)	8	19	23 (2.09)
*Deinococcus*–*Thermus*	1	1 (5)	1	1	1 (1.0)
*Cyanobacteria*	1	2 (14)	1	2	2 (1.0)
Total	43	259 (390)	284	761	825 (3.19)

* The number of genomes containing the studied MocR-TFs. The total number of analysed genomes in each taxonomic group is given in parentheses. The complete list of analysed genomes is provided in Table S1a.

† Total number of studied TFs in each taxonomic group. The mean number of TFs per genome is given in parentheses.

The entire set of 974 identified MocR-TFs was broken into taxonomic collection-specific groups that were further analysed using the comparative genomics approach (see Methods). By analysing orthologous regulators in each taxonomic group, candidate motifs and binding sites were predicted for 825 MocR-TFs in 259 bacterial genomes (Table S1a). The main outcome of this analysis is an annotated regulog, which is defined as a set of regulons controlled by orthologous TFs in a group of closely related genomes. Overall, we inferred 761 MocR-TF regulons that constitute 284 regulogs unevenly distributed across 43 studied taxonomic groups (Table 1). The reconstructed regulons included ∼1300 candidate sites and over 1800 target genes and are captured in the RegPrecise database (Data Citation 1).

Based on the phylogenetic analysis, MocR-TFs from the reconstructed regulogs were merged into 86 orthologous groups. Each group of MocR-TF orthologues generally regulates orthologous genes and is characterized by similar DNA motifs. In total, 25 of the obtained orthologous groups contain a single regulog, 19 orthologous groups include two regulogs and the remaining 42 groups consisted of three or more (maximum 12) regulogs (Table S2). The total number of MocR-TFs per orthologous group varies between one and 65, with the mean being 9.6. The most populated orthologous groups of MocR-TFs are YdcR (65 TFs, 12 regulogs) and GabR in *Proteobacteria* (40 TFs, eight regulogs). Notably, the majority of PLP metabolism regulators constitute a large number of small orthologous groups (see below).

### Structure of DNA-binding motifs of MocR-TFs

**Fig. 1.**
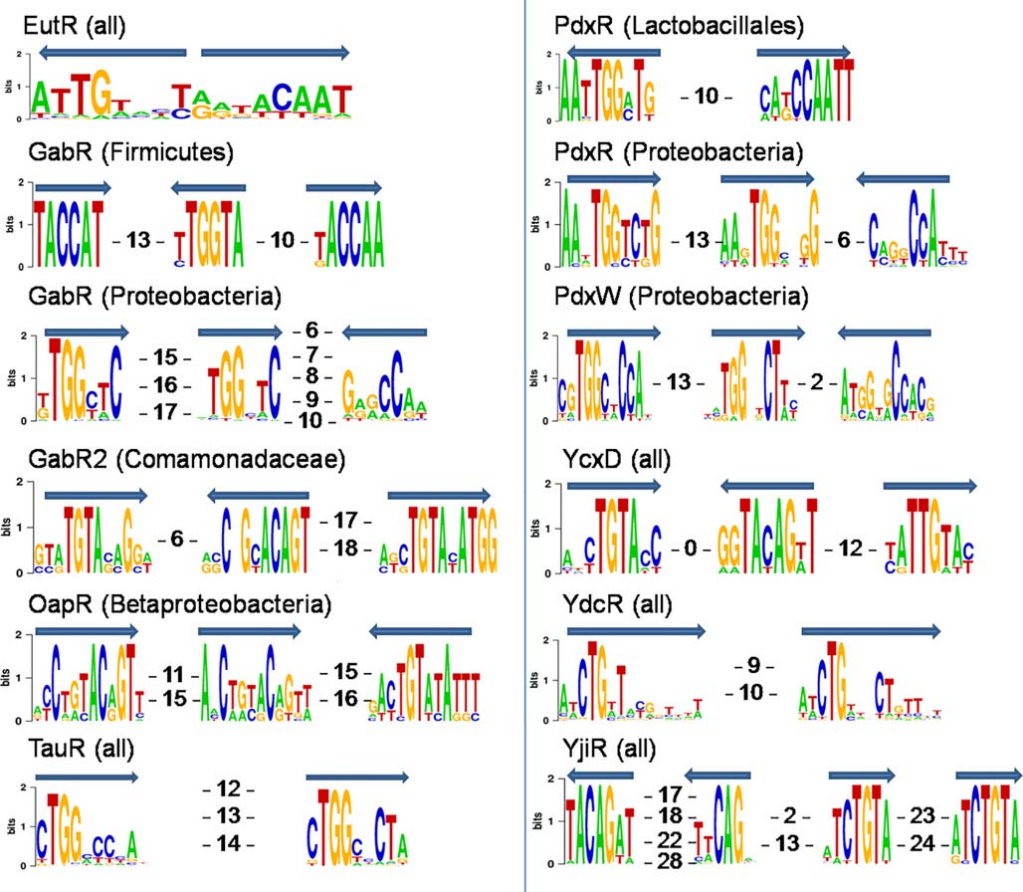
DNA binding motifs of selected MocR-subfamily TFs. Motif boxes and their orientation are shown with arrows. Numbers denote the distances between the boxes.

Direct and/or inverted repeats are prevalent among the predicted DNA-binding motifs of MocR-TFs, which is consistent with previous studies (Belitsky, 2004, 2014; Wiethaus *et al.*, 2008; Jochmann *et al.*, 2011). However, the structure of these DNA motifs, i.e. consensus sequence, and number and orientation of boxes (subunits of the motif that presumably bind a TF monomer), is highly variable (see representative examples of DNA motifs in Fig. 1). In total, 32 % of the studied regulons have motifs that consist of two direct boxes and one inverted repeat, which is the most frequent motif structure in the MocR subfamily (Table 2). Motifs with two or three direct repeats were identified in 27 % of the regulons. Motifs that comprise two inverted repeats are also quite frequently found among MocR-TFs (20 % of regulons), but such structure is not nearly as abundant as for other subfamilies of the GntR family (Rigali *et al.*, 2002; Suvorova *et al.*, 2015). Another common motif structure consists of three boxes with alternating direction (11 % of regulons). Other structures mostly comprising various four-box motifs are rare among the reconstructed regulons (Table 2). With a few exceptions, all MocR-TFs comprising a single orthologous group have similar binding motif structure.

**Table 2.** Structure of DNA binding motifs of MocR-subfamily TFs

TFBS motif structure		Regulogs	Regulons
2 boxes	→ →	70	186
	← →	57	150
3 boxes	→ → ←	103	245
	→ ← →	33	85
	→ → →	4	18
	→ ← ←	1	3
4 boxes	← ← → →	8	39
	→ ← → →	4	19
	→ → → ←	3	14
	→ → ← →	1	2

The distance between the repeats equals one or several DNA-helix turns and is generally constant for a given orthologous group, although it may vary slightly even among the members of the same regulog, which was not observed for typical binding sites of other GntR subfamilies. This may be due to the head-to-tail dimerization of MocR-subfamily TFs, as this configuration allows more flexibility compared with the head-to-head configuration.

Consensus sequence and length of the MocR-TF binding motifs vary (Fig. 1), although the majority of them include a highly conserved TGT or TGG group. Despite the differences in motif symmetry, same conserved groups are also typical for binding motifs from other subfamilies of the GntR family. This corresponds well to the data on DNA–protein interactions of the FadR repressor from *Escherichia coli* (PDB code 1H9T) and the AraR DNA-binding domain from *B. subtilis* (4EGY, 4EGZ, 4H0E), only two structures of the GntR-TFs solved in complex with DNA, as well as to the DNA–protein interactions predicted by correlation analysis for the FadR, HutC and YtrA subfamilies in general (Suvorova *et al.*, 2015).

### Functions of regulated genes and pathways

By assessing the functional content of the reconstructed regulons, we tentatively predicted their possible biological functions. MocR-subfamily regulons comprise various structural genes involved in diverse biological processes (Tables S1b and S3). Most frequently (31 of 86 orthologous groups comprising 249 of 825 studied TFs) MocR-TFs regulate genes involved in PLP metabolism, which corresponds well to the fact that these TFs are PLP-dependent (Rigali *et al.*, 2002; Tramonti *et al.*, 2015). There are also four orthologous groups of TFs that control GABA and/or putrescine utilization, two orthologous groups for regulation of taurine metabolism and one for ectoine utilization. These functional groups of regulons are described in more detail in the following sections.

Other studied MocR-TFs regulate putative enzymes from yet unknown metabolic pathways and/or putative transporters from various families. Most notably, these regulons include many PLP-dependent enzymes, such as various aminotransferases, glutamate decarboxylase, serine hydroxymethyltransferase, threonine dehydratase and threonine synthase (Tables S1b and S3) (Percudani & Peracchi, 2003). Moreover, MocR-TFs frequently regulate genes encoding proteins involved in reduction/oxidation processes, including a number of cytochromes, cytochrome oxidases, various dehydrogenases/oxidoreductases, as well as ECF σ-factor required for resistance against oxidative stress (Ryu *et al.*, 2006). Interestingly, five regulogs in four orthologous groups include a putative enamine/imine deaminase from the RidA family (Tables S1b and S3). Many PLP-dependent enzymes generate enamine/imine intermediates that can inactivate other enzymes by covalent binding to their active site, whereas the RidA-family proteins quench these reactive intermediates to prevent metabolic damage (Lambrecht *et al.*, 2012, 2013; Flynn *et al.*, 2013; Flynn & Downs, 2013). Thus, some of the identified MocR-family regulons may be involved in the response to metabolic damage caused by PLP-dependent enzymes.

### Regulons involved in PLP metabolism

Overall, we have identified 31 orthologous groups of MocR-TFs that presumably regulate genes involved in PLP metabolism (Tables S1b and S2). Notably, these groups are not clustered on the phylogenetic tree. Based on the phylogenetic and regulon content analysis we classified these regulators into three large groups, namely PdxR, PdxW and PdxQ, which are described in more detail below.

**Fig. 2.**
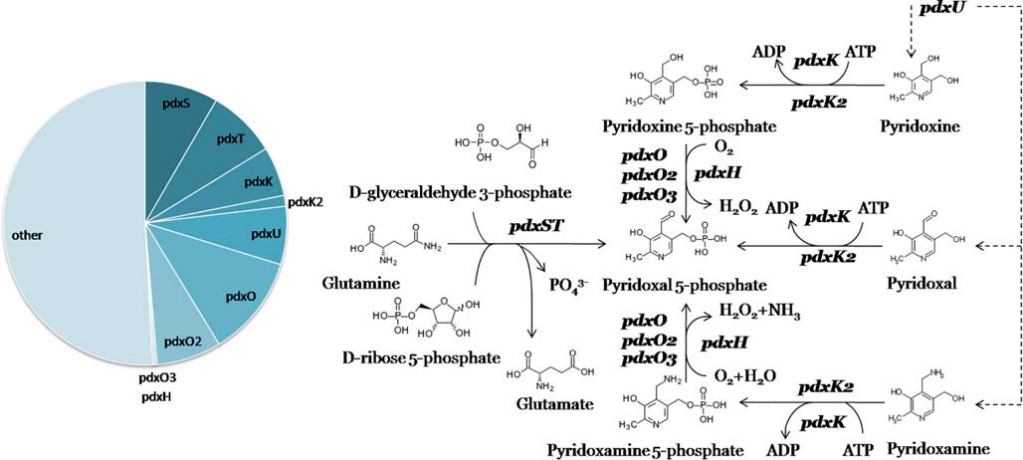
Metabolic context of the reconstructed PdxR, PdxW and PdxQ regulons involved in PLP metabolism. Members of the corresponding regulons are shown in bold type, and transport is shown as dashed lines. A pie chart of regulon content is shown.

PdxR regulons comprise 124 TFs from 11 orthologous groups that are mostly present among diverse lineages of the *Proteobacteria*, *Firmicutes* and *Actinobacteria* (Table S2). These groups include the previously described PdxR regulators from *B. clausii* (Tramonti *et al.*, 2015), *Streptococcus mutans* (Liao *et al.*, 2015), *C. glutamicum* (Jochmann *et al.*, 2011) and *L. monocytogenes* (Belitsky, 2014), and our predictions of the regulon composition and the binding motif structure for these regulators conform to known data. The main genes of the reconstructed PdxR regulons include those involved in PLP biosynthesis (*pdxST*) and/or salvage (*pdxK*) and transport (*pdxU*) (Fig. 2). Moreover, the PdxR regulons often include additional genes encoding putative pyridoxamine 5′-phosphate oxidase (*pdxO*), PLP-dependent proteins including BioF- and ArgD-family aminotransferases and GlyA-family serine hydroxymethyltransferase, PLP-binding proteins from the COG325 (Ito *et al.*, 2013; V. de Crecy-Lagard *et al.*, personal communication) and COG2258 families, a putative pyridoxine transporter from the BenE family, RimI- and RimL-family acetyltransferases, GuaA-family amidotransferase, DapA-family dihydrodipicolinate synthase, and several other metabolic and transport genes (Tables S1b and S3).

We have also characterized 14 novel orthologous groups of PdxW regulators (66 TFs) and six groups of PdxQ regulators (59 TFs), most of which are present among various lineages of *Proteobacteria* and less commonly found among *Actinobacteria*, *Firmicutes* and *Bacteroidetes* (Table S2). PdxW regulons generally include very few genes, mainly putative pyridoxamine 5′-phosphate oxidases (*pdxO*, *pdxO2*, *pdxO3*), and additionally RimI- and RimL-family acetyltransferases and OsmC-family peroxiredoxin, but do not contain transporters. In contrast, the PdxQ regulons include both PLP-metabolic genes (*pdxO*, *pdxO2*, *pdxH*) and genes encoding putative transporters from the drug/metabolite transporter (DMT) superfamily or LysE/YggA family. Additional members of PdxQ regulons include PLP-dependent aminotransferases, CsdA-family cysteine desulfurase, RimI-family acetyltransferase, DapA-family dihydrodipicolinate synthase, GloA-family lyase, COG384-family protein, and some other metabolic and transport genes (Tables S1b and S3). COG384 is a PhzF-related epimerase/isomerase, but the corresponding genes in PdxQ regulons are not co-localized with the other known phenazine biosynthesis genes (Mavrodi *et al.*, 1998; Blankenfeldt *et al.*, 2004). Notably, the phenazine biosynthesis pathway oxidase PhzG is homologous to pyridoxine oxidase PdxH (Parsons *et al.*, 2004). Thus, the PdxQ-regulated COG384 might be involved in PLP metabolism.

Several enzymes and transporters identified in PLP metabolic regulons (e.g. proteins from the BenE, GlyA, DapA, PhzF, RimI, RimL, RhtB and RhaT families) were also frequently found in other MocR-TF regulons with unknown metabolic function (Tables S1b and S3).

### Ectoine utilization regulons

**Fig. 3.**
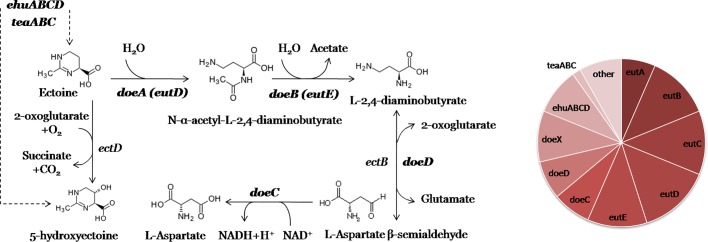
Metabolic context of the reconstructed EutR regulons involved in ectoine utilization. Members of the corresponding regulons are shown in bold type, and transport is shown as dashed lines. A pie chart of regulon content is shown.

Imino acid ectoine can be used by bacteria, predominantly halophilic bacterial, as sole energy, carbon and nitrogen source or as a compatible solute that helps the cell adapt to the high environmental osmolality and other stress conditions (Grammann *et al.*, 2002; Jebbar *et al.*, 2005; Schwibbert *et al.*, 2011). Such osmoprotectants can be either synthesized in the cell or exported from the medium. The putative ectoine utilization pathway was proposed for *Halomonas elongata* (Schwibbert *et al.*, 2011) (Fig. 3). Ectoine is presumably hydrolysed via hydrolase DoeA, followed by conversion to l-2,4-diaminobutyric acid (DABA) by deacetylase DoeB. DABA is metabolized via transaminase DoeD to l-aspartate-β-semialdehyde, which is then oxidized to l-aspartate by dehydrogenase DoeC. Ectoine utilization genes in *H. elongata* are predicted to be regulated by the AsnC/Lrp-family regulator DoeX (Schwibbert *et al.*, 2011). Ectoine-induced genes were also described in *Sinorhizobium meliloti* and include putative ectoine/hydroxyectoine ABC transport system genes *ehuABCD*, ectoine catabolic genes *eutABCDE*, and orthologues of *doeC*, *doeD*, *doeX* and a putative oxidoreductase (COG604) (Jebbar *et al.*, 2005). The *eutD* and *eutE* genes of *Sinorhizobium meliloti* are orthologues of *doeA* and *doeB* in *H. elongata*, respectively. Orthologues of *eutB* and *eutC* are also present near the *doe* genes in *H. elongata*, although an orthologue of *eutA* is absent. The role of *eutB* and *eutC* in ectoine degradation is arguable (Schwibbert *et al.*, 2011), but it is possible that they can participate in another ectoine utilization pathway, as yet unknown. Cyclodeaminase EutC might be involved in the cleavage of the ectoine ring structure. One of the further intermediates is presumably l-threonine, which can be converted to 2-oxobutyrate by threonine dehydratase EutB.

The predicted MocR-subfamily regulator for ectoine transport and utilization, EutR, was identified in many species of the orders *Rhizobiales* (including *Sinorhizobium meliloti*) and *Rhodobacterales*, as well as in some species of betaproteobacteria and gammaproteobacteria. The predicted EutR palindromic binding motif is conserved in all studied *Proteobacteria* (Fig. 1). Most of the reconstructed EutR regulons include the *eutABCDE*, *doeC*, *doeD, doeX* and *ehuABCD* genes (Table S1b). COG604 is also regulated by EutR in several *Rhizobiales* species. The TeaABC TRAP transport system, which mediates the uptake of external ectoine and hydroxyectoine in response to osmotic shock (Grammann *et al.*, 2002), belongs to EutR regulons in *Oceanicola batsensis* and *Silicibacter pomeroyi*, two *Rhodobacterales* species that lack the EhuABCD transporter. A gene encoding a homologue of universal stress protein UspA from *E. coli* is found downstream of *teaABC* in these genomes and is probably transcribed along with it, which is consistent with previous studies on *H. elongata* (Grammann *et al.*, 2002). As ectoine is involved in stress response, *uspA* might indeed be a part of the EutR regulon. Only a few *Rhodobacterales* have *ehu* and *eut* genes in their EutR regulons, while most of these regulons include an uncharacterized gene encoding YjjS-related protein (COG5457), which is also frequently present in other MocR-TF regulons (Table S1b).

### GABA utilization regulons

**Fig. 4.**
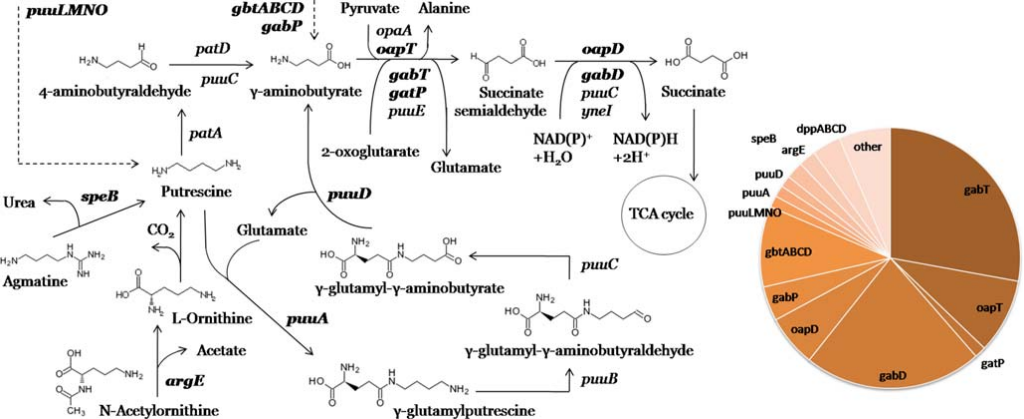
Metabolic context of the reconstructed GabR and OapR regulons involved in GABA utilization. Members of the corresponding regulons are shown in bold type, and transport is shown as dashed lines. A pie chart of regulon content is shown.

GABA is an important nitrogen and carbon source for many bacteria that is produced intracellularly from putrescine or glutamate or imported into the cell (Belitsky & Sonenshein, 2002; Prell *et al.*, 2009; Dhakal *et al.*, 2012). The GABA utilization pathway starts from transamination by various GABA- or ω-aminotransferases that involves keto acid acceptors (2-oxoglutarate, pyruvate) and yields succinic semialdehyde, and proceeds by oxidation of succinic semialdehyde to succinate via various NAD(P)-dependent dehydrogenases (Fig. 4) (Prell *et al.*, 2009; Kurihara *et al.*, 2010; Schneider & Reitzer, 2012). In most bacteria, GABA is transported by permease GabP and utilized by GABA aminotransferase GabT and dehydrogenase GabD.

Polyamines (e.g. putrescine) play important roles in various processes such as protein synthesis, stress response, cell growth and development. Putrescine is produced from ornithine or from arginine via agmatine and can be used as a sole carbon and nitrogen source. Putrescine is converted to GABA using either the γ-glutamylated putrescine pathway that involves the PuuABCD enzymes, or the aminotransferase pathway that involves PLP-dependent aminotransferase PatA and NAD-dependent dehydrogenases PatD or PuuC (Fig. 4) (Kurihara *et al.*, 2010; Schneider & Reitzer, 2012; Cha *et al.*, 2014).

We identified four orthologous groups of MocR-TFs that probably control GABA and/or putrescine utilization. The group of GabR regulators in *Firmicutes* includes orthologues of the previously characterized GabR in *B. subtilis* (Belitsky & Sonenshein, 2002; Belitsky, 2004) identified only in several other *Bacillaceae* and *Staphylococcaceae* genomes. The reconstructed GabR regulons in *Firmicutes* include only the *gabT*, *gabD* and *gabP* genes and no novel regulon members. Using comparative genomics, we refine the structure of the GabR binding motif that was identified in previous studies (Belitsky, 2004). We propose a novel conserved structure of the GabR operator motif that contains an inverted repeat between two direct repeats (Fig. 1).

In *Proteobacteria*, we identified two non-orthologous groups of GABA regulators, GabR and GabR2. Members of the GabR group are widely distributed in various lineages of betaproteobacteria and gammaproteobacteria (28 genomes from eight taxonomic groups), whereas GabR2 regulators were found only in six species from the *Comamonadaceae* and *Pseudomonadaceae* lineages (Table S2). The predicted GabR binding motif in *Proteobacteria* consists of two direct repeats and one inverted repeat, the distance between them varying slightly among lineages (Fig. 1). The GabR2 binding motif in *Comamonadaceae* has three boxes with alternating direction (Fig. 1), while in *Pseudomonadaceae* it is an imprecise 12 nt palindrome with consensus resembling the first box of the *Comamonadaceae* motif.

The content of reconstructed GabR/GabR2 regulons in *Proteobacteria* varies (Table S1b). Among betaproteobacteria, GabR regulates *gabT* and *gabD* (but not *gabP*) in most genomes, as well as several other novel regulon members (see below). There are several paralogues of GabR in all studied *Alcaligenaceae* and most *Burkholderia* species. According to genomic co-localization, one of these paralogues regulates *gabT* and *gabD*, whereas the other one controls genes encoding: (i) putative GABA ABC-transporter *gbtABCD* in *Burkholderia* species, and (ii) GABA aminotransferase *gabT2* and putative dipeptide ABC-transporter *dppABCD* in *Bordetella* species. The above ABC transporters also belong to the GabR regulon in *Ralstonia* species. The GabR regulon in *Bordetella avium* also includes *gabT3*, COG2334-family kinase (notably, these genes often merge into one multi-domain gene with *gabT* in many *Proteobacteria* that lack GabR), and acetylornithine deacetylase *argE* that deacetylates *N*-acetylornithine to l-ornithine, which is further converted to putrescine, a compound metabolically related to GABA (Fig. 4). ArgE is also present in GabR regulons of two *Ralstonia* species. GabR regulons in some *Comamonadaceae* include a novel PLP-dependent GABA aminotransferase *gatP* (instead of homologous *gabT*) and genes encoding enzymes involved in putrescine metabolism (*puuA*, *puuD*), as well as an ABC transporter homologous to *gbtABCD*. The latter transporter is probably involved in putrescine transport and was thus named *puuLMNO* (Fig. 4). The XRE-family regulator PuuR, which represses *puu* genes in *E. coli* (Kurihara *et al.*, 2010; Schneider & Reitzer, 2012), is absent in *Comamonadaceae*, and thus their GabR regulons are presumably expanded to include the related putrescine metabolic pathway. Among gammaproteobacteria, GabR regulons include *gabT*, *gabD*, and *gabP* or *gbtBCD*. Finally, the reconstructed GabR2 regulons include: (i) *gabP* in *Pseudomonadaceae*; and (ii) *gabT*, *gabD*, *gbtABCD* and *speB* encoding agmatinase that produces putrescine from agmatine in *Comamonadaceae* (Fig. 4) (Cha *et al.*, 2014). Notably, in all studied GabR/GabR2 regulons in *Proteobacteria*, *gabP*, *gbtABCD* and *dppABCD* are never present simultaneously, thus being alternative transporters.

Another orthologous group of MocR-TFs potentially involved in GABA utilization, named OapR, was identified in several alphaproteobacteria and betaproteobacteria (13 genomes from five taxonomic groups). The reconstructed OapR regulons contain the predicted alternative enzymes from the GABA utilization pathway, ω-amino acid–pyruvate aminotransferase *oapT* and succinic semialdehyde dehydrogenase *oapD* (Fig. 4). Predicted OapR binding motifs differ between the regulogs, although the sequence of a single box is similar in the whole group. In betaproteobacteria, the motif consists of two direct repeats and one inverted repeat (Fig. 1), in *Rhizobiales*, it has three boxes with alternating direction, and in *Rhodospirillales*, it lacks the first box and consists of two inverted repeats.

### Taurine utilization regulons

**Fig. 5.**
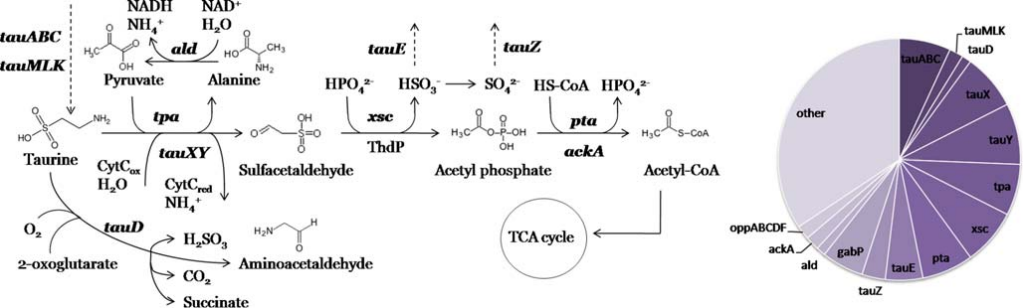
Metabolic context of the reconstructed TauR regulons involved in taurine utilization. Members of the corresponding regulons are shown in bold type, and transport is shown as dashed lines. A pie chart of regulon content is shown.

Taurine (2-aminoethanesulfonate) is a widespread natural product that can be used as an osmolyte and a source of energy, carbon, nitrogen or sulfur for growth by various bacteria (Denger *et al.*, 2004, 2006; Weinitschke *et al.*, 2007; Felux *et al.*, 2013). Taurine can be synthesized (e.g. from cysteine) or imported into the cell (e.g. by TauABC or TauMLK transporters). There are two alternative taurine utilization pathways active under different metabolic conditions (Fig. 5). In the first pathway, widespread among bacteria, taurine is converted to sulfoacetaldehyde via either Tpa aminotransferase (coupled with conversion of alanine to pyruvate via dehydrogenase Ald) or TauXY dehydrogenase. Acetyltransferase Xsc converts sulfoacetaldehyde to acetyl phosphate and the sulfite ion. Sulfite is either exported by TauE transporter or is oxidized to sulfate and further exported via TauZ. Acetyl phosphate is further converted to acetyl-CoA, either by transacetylase Pta or by acetate kinase AckA and acetate-CoA ligase. This pathway is used for dissimilation of taurine as a carbon and nitrogen source, possibly serving also for sulfite detoxification and maintaining cell osmolarity (Denger *et al.*, 2004). The second pathway is used for utilization of taurine as a source of sulfur for aerobic growth and includes desulfonation of taurine to aminoacetaldehyde by dioxygenase TauD (Denger *et al.*, 2004; Felux *et al.*, 2013).

Taurine utilization is described in many bacteria, for example *Paracoccusdenitrificans* (Felux *et al.*, 2013), *Ralstonia eutropha* (Weinitschke *et al.*, 2007), *Rhodobacter sphaeroides* (Denger *et al.*, 2006) and *Rhodococcus* species (Denger *et al.*, 2004). TauR from the MocR subfamily was predicted and further experimentally confirmed to be a transcriptional activator of taurine metabolism (Wiethaus *et al.*, 2008, Denger *et al.*, 2004, Mostafavi *et al.*, 2014). We identified TauR orthologues in diverse alpha-, beta- and gammaproteobacteria (24 genomes from eight taxonomic groups) and in two *Rhodococcus* species from the phylum *Actinobacteria* (Table S2). TauR in *Rhodobacter capsulatus* is known to bind direct repeats with the consensus CTGGACYTAA (Wiethaus *et al.*, 2008). All reconstructed TauR regulons have a conserved DNA motif with similar consensus sequence and structure (Fig. 1). However, the content of TauR regulons is highly variable even between closely related genomes (Table S1b).

The reconstructed TauR regulons in alphaproteobacteria include the taurine transporters *tauABC* and *tauMLK*, the taurine utilization pathway genes *tauXY*, *tpa*, *xsc*, *pta* and *ackA*, as well as the sulfite/sulfate exporters *tauE* and *tauZ*. Notably, the enzymes Pta and AckA, catalysing alternative means of acetyl phosphate conversion, are never present in TauR regulons simultaneously. TauR regulons in some *Rhodobacterales* also include *bisC*, *dmsC* and *hybA*, which encode 4Fe–4S ferredoxin oxidoreductase subunits and may function as the potential electron acceptor for cytochrome *c* coupled TauXY. In some genomes of *Rhodobacterales* TauR also regulates genes encoding a Tpa-related aminotransferase (COG161), an oxidoreductase (COG665) and an alkylhydroperoxidase (COG2128). The content of TauR regulons in betaproteobacteria is mostly similar to that in alphaproteobacteria, excluding *tpa*, *tauZ* and *ackA*, and with a few additional members: (i) the permease GabP, RimI-family acetyltransferase and a putative aldehyde dehydrogenase (COG1012) in *Burkholderia*; (ii) taurine dioxygenase TauD in two *Ralstonia* species; and (iii) the permease GabP and the putative oligopeptide ABC transporter OppABCDF, as well as COG523- and COG1574-family proteins in *Comamonadaceae* (Tables S1b and S3). In gammaproteobacteria, the predicted TauR regulons comprise *tauABC*, *tpa* and genes encoding a putative amino acid ABC transporter. TauR regulons in *Actinobacteria* include *tauE*, *tpa*, *pta*, *ald*, *gabP*, the thiazole biosynthesis gene *thiG* and *pdxS*.

We have also identified another group of closest TauR homologues in two *Rhodobacterales* and one *Rhizobiales* species, named TauR2. The predicted TauR2 binding motif is similar to the TauR motif. The content of the reconstructed TauR2 regulons resembles that of TauR regulons in *Comamonadaceae*. TauR2 regulons include *tauE*, *oppABCDF*, genes encoding COG523- and COG1574-family proteins, as well as TRAP transporters of unknown specificity, a putative aldehyde dehydrogenase (COG1012) and hydrolase (COG596) (Tables S1b and S3).

### Common features in MocR-TF regulated pathways

Notably, GABA, ectoine and taurine and their corresponding transport and utilization pathways share some similarities. DABA, a precursor of ectoine, is an analogue of GABA and is known to reduce its uptake, while both GABA and DABA inhibit taurine transport into the cell, since all these compounds are structurally related (Kontro & Oja, 1981). The GABA, ectoine and taurine metabolic pathways include a transamination step carried out by homologous aminotransferases, GabT/GatP/OapT, DoeD and Tpa, respectively. Reactions following PLP-dependent transamination in the GABA and ectoine utilization pathways also involve homologous enzymes, the GabD/OapD and DoeC dehydrogenases, respectively. It is also known that proteins involved in GABA transport (GabP) and metabolism (GabT and GabD) can also participate in uptake of homotaurine (3-aminopropanesulfonate, a homologue of taurine and a sulfonate analogue of GABA) and its conversion to 3-sulfopropanoate (Mayer & Cook, 2009). Thus, it may explain the occurrence of GabP and aldehyde dehydrogenases (COG1012) related to GabD in some TauR and TauR2 regulons. The presence of homologous oligopeptide ABC transporters in GabR (DppABCD), and TauR and TauR2 (OppABCDF) regulons also possibly reflects this connection between GABA and taurine metabolism.

## Conclusions

We used the comparative genomics approach to identify candidate binding sites and reconstruct apparent regulons of TFs from the MocR subfamily of the GntR family, which is broadly distributed in *Bacteria* but not present in *Archaea*. We have identified putative DNA binding motifs for 825 MocR-TFs in 259 bacterial genomes and analysed their structure. The predicted motifs are generally similar within each orthologous group and consist of two to four repeated boxes in different orientation. Motifs with direct and/or inverted repeats are most common, suggesting preferable dimerization of MocR-TFs in head-to-tail or head-to-head configuration.

By analysing putative functional roles of regulated genes we revealed that over one-third of analysed MocR-TFs control genes involved in vitamin B6 biosynthesis and salvage and other aspects of PLP metabolism, as well as several PLP-dependent enzymes. Among other functionally characterized MocR-TFs are regulators of utilization of organic osmolytes such as ectoine and taurine, and utilization of the non-protein amino acid GABA. By reconstructing MocR-TFs regulons, we have identified several novel members of these metabolic systems. In particular, the reconstructed GABA utilization regulons in *Proteobacteria* contain novel GABA aminotransferases (GatP, OapT) and succinic semialdehyde dehydrogenase (OapD) that are alternative to GabT and GabD, respectively, as well as novel transport systems (GbtABCD and DppABCD). Interestingly, presumed GabR regulons in betaproteobacteria are expanded to include components of adjacent metabolic pathways, putative putrescine transporter (PuuLMNO) and *N*-acetylornithine and putrescine utilization enzymes (PuuAD, ArgE, SpeB). EutR regulons additionally include universal stress protein (UspA), which is consistent with the fact that ectoine is involved in stress response. Additional members of TauR regulons include the putative ferredoxin oxidoreductase subunits (BisC, DmsC, HybA) that may function as a potential electron acceptor for taurine dehydrogenase TauXY, the GabP and OppABCDF transporters and a GabD-family dehydrogenase, which possibly reflects the connection between GABA and taurine metabolism.

Moreover, we predict novel functional connections between several types of enzymes and/or transporters, including those with unknown function, based on their co-occurrence in the MocR-TF regulons such as BenE-family transporter with GlyA-family serine hydroxymethyltransferase, PhzF-like epimerase/isomerase with transporters of the DMT superfamily, and RimI- and RimL-family acetyltransferases with PLP metabolic enzymes.

The comparative genomics analysis revealed DNA motifs and regulon contents for a large number of MocR-TFs in bacteria. The majority of inferred regulons, including their target DNA motifs and regulated genes, constitute novel regulatory interactions that await future experimental testing.

## Supplementary Data

421 Supplementary Data
