# Supplementary material for: Comparative genomics of pyridoxal 5′-phosphate-dependent transcription factor regulons in Bacteria
Source: Microb Genom. 2016 Jan 18;2(1):e000047. doi: 10.1099/mgen.0.000047 (PMC5320631; doi:10.1099/mgen.0.000047)
Supplement: Supplementary file 1 — Supplementary Data [file mgen-02-47-s001.pdf]

Manuscript Including References (Word document) Click here to download Manuscript Including References (Word document) Supplementary_Tables_S1-2-3_revised.pdf

Table S1a. Studied bacterial genomes and MocR-TF regulogs.

Tax ID	Phylum / Class / Taxonomic collection [1] / Genome [2]	Number of analyzed MocR-TFs*	Total number of MocR-TFs**	Number of genomes per taxon	Number of regulogs per taxon
Phylum	Proteobacteria	600	685	199	212
Class	Gammaproteobacteria	208	237	90	81
	Enterobacteriales	48	51	12	15
290338	Citrobacter koseri ATCC BAA-895	5	5		
498217	Edwardsiella tarda EIB202	0	1		
399742	Enterobacter sp. 638	7	7		
716540	Erwinia amylovora ATCC 49946	3	3		
218491	Erwinia carotovora subsp. atroseptica SCRI1043	7	7		
511145	Escherichia coli str. K-12 substr. MG1655	2	2		
272620	Klebsiella pneumoniae subsp. pneumoniae MGH 78578	8	9		
243265	Photorhabdus luminescens subsp. laumondii TTO1	1	1		
529507	Proteus mirabilis HI4320	1	1		
99287	Salmonella typhimurium LT2	3	3		
399741	Serratia proteamaculans 568	9	10		
187410	Yersinia pestis KIM	2	2		
	Pasteurellales	3	3	9	1
537457	Actinobacillus pleuropneumoniae serovar 7 str. AP76	1	1		
339671	Actinobacillus succinogenes 130Z	0	0		
634176	Aggregatibacter aphrophilus NJ8700	0	0		
233412	Haemophilus ducreyi 35000HP	1	1		
71421	Haemophilus influenzae Rd KW20	0	0		
557723	Haemophilus parasuis SH0165	0	0		
228400	Haemophilus somnus 2336	0	0		
221988	Mannheimia succiniciproducens MBEL55E	0	0		
272843	Pasteurella multocida subsp. multocida str. Pm70	1	1		
	Vibrionales	30	30	10	7
298386	Photobacterium profundum SS9	4	4		
314292	Vibrio angustum S14	5	5		
243277	Vibrio cholerae O1 biovar eltor str. N16961	1	1		
312309	Vibrio fischeri ES114	0	0		
338187	Vibrio harveyi ATCC BAA-1116	3	3		
223926	Vibrio parahaemolyticus RIMD 2210633	4	4		
316275	Vibrio salmonicida LFI1238	1	1		
391591	Vibrio shilonii AK1	3	3		
575788	Vibrio splendidus LGP32	5	5		
216895	Vibrio vulnificus CMCP6	4	4		
	Psychromonadaceae/Aeromonadales	12	12	6	7
380703	Aeromonas hydrophila subsp. hydrophila ATCC 7966	3	3		
382245	Aeromonas salmonicida subsp. salmonicida A449	2	2		
58051	Moritella sp. PE36	2	2		
357804	Psychromonas ingrahamii 37	2	2		
314282	Psychromonas sp. CNPT3	1	1		
595494	Tolumonas auensis DSM 9187	2	2		
	Shewanellaceae	23	25	16	6
326297	Shewanella amazonensis SB2B	1	1		
325240	Shewanella baltica OS155	5	5		
318161	Shewanella denitrificans OS217	0	0		
318167	Shewanella frigidimarina NCIMB 400	0	0		
458817	Shewanella halifaxensis HAW-EB4	1	1		
323850	Shewanella loihica PV-4	0	0		
211586	Shewanella oneidensis MR-1	1	1		
398579	Shewanella pealeana ATCC 700345	1	1		
225849	Shewanella piezotolerans WP3	1	1		

319224	Shewanella putrefaciens CN-32	3	4		
425104	Shewanella sediminis HAW-EB3	0	0		
94122	Shewanella sp ANA-3	2	2		
60480	Shewanella sp MR-4	1	1		
60481	Shewanella sp MR-7	2	2		
351745	Shewanella sp W3-18-1	3	3		
392500	Shewanella woodyi ATCC 51908	2	3		
	**Alteromonadales**	7	7	9	4
342610	Pseudoalteromonas atlantica T6c	0	0		
314275	Alteromonas macleodii 'Deep ecotype'	0	0		
455436	Glaciecola sp. HTCC2999	0	0		
167879	Colwellia psychrerythraea 34H	3	3		
156578	Alteromonadales bacterium TW-7	0	0		
326442	Pseudoalteromonas haloplanktis TAC125	1	1		
87626	Pseudoalteromonas tunicata D2	3	3		
314276	Idiomarina baltica OS145	0	0		
283942	Idiomarina loihiensis L2TR	0	0		
	**Oceanospirillales/Alteromonadales**	29	30	12	16
393595	Alcanivorax borkumensis SK2	0	0		
498211	Cellvibrio japonicus Ueda107	2	2		
290398	Chromohalobacter salexigens DSM 3043	5	5		
349521	Hahella chejuensis KCTC 2396	6	6		
351348	Marinobacter aqueolei	2	2		
270374	Marinobacter sp. ELB17	2	2		
400668	Marinomonas sp. MWYL1	5	6		
207949	Oceanobacter sp. RED65	0	0		
207954	Oceanospirillum sp. MED92	4	4		
314283	Reinekea sp. MED297	1	1		
203122	Saccharophagus degradans 2-40	1	1		
377629	Teredinibacter turnerae T7901	1	1		
	**Pseudomonadaceae**	38	60	8	13
322710	Azotobacter vinelandii AvOP	1	2		
208964	Pseudomonas aeruginosa PAO1	6	8		
384676	Pseudomonas entomophila L48	6	12		
220664	Pseudomonas fluorescens Pf-5	9	12		
399739	Pseudomonas mendocina ymp	5	8		
160488	Pseudomonas putida KT2440	8	11		
379731	Pseudomonas stutzeri A1501	1	3		
223283	Pseudomonas syringae pv. tomato str. DC3000	2	4		
	**Moraxellaceae**	11	12	4	7
62977	Acinetobacter sp. ADP1	4	4		
480119	Acinetobacter baumannii AB0057	7	7		
259536	Psychrobacter arcticum 273-4	0	1		
349106	Psychrobacter sp. PRwf-1	0	0		
	**Xanthomonadales**	7	7	4	5
522373	Stenotrophomonas maltophilia K279a	5	5		
190486	Xanthomonas axonopodis pv. citri str. 306	1	1		
190485	Xanthomonas campestris pv. campestris str. ATCC 33913	1	1		
160492	Xylella fastidiosa 9a5c	0	0		
Class	**Betaproteobacteria**	268	301	40	82
	**Alcaligenaceae**	27	28	3	11
360910	Bordetella avium 197N	9	10		
257310	Bordetella bronchiseptica RB50	9	9		
340100	Bordetella petrii DSM 12804	9	9		
	**Ralstonia**	59	59	6	18
164546	Cupriavidus taiwanensis	10	10		
381666	Ralstonia eutropha H16	15	15		

264198	Ralstonia eutropha JMP134	11	11		
266264	Ralstonia metallidurans CH34	10	10		
402626	Ralstonia pickettii 12J	7	7		
267608	Ralstonia solanacearum GMI1000	6	6		
	**Burkholderia**	100	121	8	19
339670	Burkholderia cepacia AMMD (Burkholderia ambifaria AMMD)	15	19		
626418	Burkholderia glumae BGR1	9	10		
243160	Burkholderia mallei ATCC 23344	7	9		
391038	Burkholderia phymatum STM815	20	24		
272560	Burkholderia pseudomallei K96243	9	11		
269483	Burkholderia sp. 383	16	19		
269482	Burkholderia vietnamiensis G4	12	13		
266265	Burkholderia xenovorans LB400	12	16		
	**Comamonadaceae**	61	68	11	23
397945	Acidovorax avenae subsp. citrulli AAC00-1	2	3		
232721	Acidovorax sp. JS42	1	2		
399795	Comamonas testosteroni KF-1	6	9		
398578	Delftia acidovorans SPH-1	15	16		
395495	Leptothrix cholodnii SP-6	2	3		
420662	Methylibium petroleiphilum PM1	6	6		
365044	Polaromonas naphthalenivorans CJ2	7	7		
296591	Polaromonas sp. JS666	5	5		
338969	Rhodoferax ferrireducens DSM 15236	3	3		
543728	Variovorax paradoxus S110	9	9		
391735	Verminephrobacter eiseniae EF01-2	5	5		
	**Various betaproteobacteria**	21	25	12	11
76114	Azoarcus sp. EbN1	2	3		
243365	Chromobacterium violaceum ATCC 12472	5	6		
159087	Dechloromonas aromatica RCB	2	2		
557598	Laribacter hongkongensis HLHK9	2	2		
265072	Methylobacillus flagellatus KT	2	3		
383631	Methylophilales bacterium HTCC2181	0	0		
583345	Methylotenera mobilis JLW8	3	3		
122586	Neisseria meningitidis MC58	0	0		
228410	Nitrosomonas europaea ATCC 19718	0	0		
323848	Nitrosospira multiformis ATCC 25196	0	1		
85643	Thauera sp. MZ1T	4	4		
292415	Thiobacillus denitrificans	1	1		
**Class**	**Alphaproteobacteria**	**105**	**126**	**50**	**42**
	**Rhizobiales**	48	55	15	17
176299	Agrobacterium tumefaciens str. C58 (Cereon)	4	4		
438753	Azorhizobium caulinodans ORS 571	5	5		
283165	Bartonella quintana str. Toulouse	0	0		
224911	Bradyrhizobium japonicum USDA 110	5	7		
288000	Bradyrhizobium sp. BTAi1	4	4		
224914	Brucella melitensis 16M	1	1		
266835	Mesorhizobium loti MAFF303099	6	8		
266779	Mesorhizobium sp. BNC1	2	3		
323098	Nitrobacter winogradskyi Nb-255	1	1		
347834	Rhizobium etli CFN 42	4	4		
216596	Rhizobium leguminosarum bv. viciae 3841	4	5		
394	Rhizobium sp. NGR234	4	4		
258594	Rhodopseudomonas palustris CGA009	2	3		
266834	Sinorhizobium meliloti 1021	5	5		
78245	Xanthobacter autotrophicus Py2	1	1		
	**Rhodobacterales**	34	38	15	11
228405	Hyphomonas neptunium ATCC 15444	0	0		

290400	Jannaschia sp. CCS1	1	1		
314232	Loktanella vestfoldensis SKA53	2	2		
314254	Oceanicaulis alexandrii HTCC2633	1	1		
252305	Oceanicola batsensis HTCC2597	2	2		
314256	Oceanicola granulosus HTCC2516	0	0		
318586	Paracoccus denitrificans PD1222	6	6		
272943	Rhodobacter sphaeroides 2.4.1	3	4		
314271	Rhodobacterales bacterium HTCC2654	0	0		
314262	Roseobacter sp. MED193	2	2		
89187	Roseovarius nubinhibens ISM	4	4		
314264	Roseovarius sp. 217	3	3		
246200	Silicibacter pomeroyi DSS-3	7	9		
292414	Silicibacter TM1040	2	3		
52598	Sulfitobacter sp. EE-36	1	1		
	**Rhodospirillales**	11	20	9	6
634452	Acetobacter pasteurianus IFO 3283-01	2	2		
137722	Azospirillum sp. B510	0	5		
272568	Gluconacetobacter diazotrophicus PAl 5	3	6		
290633	Gluconobacter oxydans 621H	0	0		
391165	Granulibacter bethesdensis CGDNIH1	0	0		
342108	Magnetospirillum magneticum AMB-1	1	1		
272627	Magnetospirillum magnetotacticum MS-1	1	1		
414684	Rhodospirillum centenum SW	0	1		
269796	Rhodospirillum rubrum ATCC 11170	4	4		
	**Sphingomonadales**	5	6	7	4
314225	Erythrobacter litoralis HTCC2594	0	0		
237727	Erythrobacter sp. NAP1	0	0		
279238	Novosphingobium aromaticivorans DSM 12444	0	0		
452662	Sphingobium japonicum UT26S	0	1		
392499	Sphingomonas wittichii RW1	3	3		
317655	Sphingopyxis alaskensis RB2256	0	0		
264203	Zymomonas mobilis subsp. mobilis ZM4	2	2		
	**Caulobacterales**	7	7	4	4
190650	Caulobacter crescentus CB15	1	1		
509190	Caulobacter segnis ATCC 21756	2	2		
366602	Caulobacter sp. K31	2	2		
450851	Phenylobacterium zucineum HLK1	2	2		
**Class**	**Proteobacteria/Delta**	**19**	**21**	**19**	**7**
	**Desulfovibrionales**	12	14	10	4
485915	Desulfohalobium retbaense DSM 5692	1	1		
525897	Desulfomicrobium baculatum DSM 4028	2	2		
207559	Desulfovibrio desulfuricans G20	1	1		
525146	Desulfovibrio desulfuricans subsp. desulfuricans str. ATCC 27774	1	1		
573370	Desulfovibrio magneticus RS-1	1	1		
411464	Desulfovibrio piger ATCC 29098	0	0		
526222	Desulfovibrio salexigens DSM 2638	1	2		
882	Desulfovibrio vulgaris Hildenborough	3	3		
883	Desulfovibrio vulgaris str. Miyazaki F	2	3		
363253	Lawsonia intracellularis PHE/MN1-00	0	0		
	**Desulfuromonadales**	7	7	9	3
281689	Desulfuromonas acetoxidans DSM 684	1	1		
398767	Geobacter lovleyi SZ	0	0		
269799	Geobacter metallireducens GS-15	0	0		
316067	Geobacter sp. FRC-32	1	1		
443144	Geobacter sp. M21	2	2		
243231	Geobacter sulfurreducens PCA	1	1		
351605	Geobacter uraniireducens Rf4	1	1		

338963	Pelobacter carbinolicus str. DSM 2380	1	1		
338966	Pelobacter propionicus DSM 2379	0	0		
**Phylum**	**Firmicutes**	**93**	**134**	**86**	**31**
**Class**	**Bacilli**	**64**	**86**	**54**	**22**
	**Bacillales**	31	44	11	10
491915	Anoxybacillus flavithermus WK1	0	0		
326423	Bacillus amyloliquefaciens FZB42	4	4		
226900	Bacillus cereus ATCC 14579	4	9		
66692	Bacillus clausii KSM-K16	2	3		
272558	Bacillus halodurans C-125	3	4		
279010	Bacillus licheniformis DSM 13	3	6		
315750	Bacillus pumilus SAFR-032	2	4		
224308	Bacillus subtilis subsp. subtilis str. 168	7	7		
235909	Geobacillus kaustophilus HTA426	1	1		
221109	Oceanobacillus iheyensis HTE831	1	1		
324057	Paenibacillus sp. JDR-2	3	5		
	**Staphylococcaceae**	12	12	7	4
458233	Macrococcus caseolyticus JCSC5402	1	1		
158879	Staphylococcus aureus subsp. aureus N315	2	2		
553212	Staphylococcus capitis SK14	1	1		
396513	Staphylococcus carnosus subsp. carnosus TM300	2	2		
176280	Staphylococcus epidermidis ATCC 12228	1	1		
279808	Staphylococcus haemolyticus JCSC1435	1	1		
342451	Staphylococcus saprophyticus subsp. saprophyticus ATCC 15305	4	4		
	**Streptococcaceae**	8	16	15	2
272622	Lactococcus lactis subsp. cremoris SK11	0	0		
272623	Lactococcus lactis subsp. lactis Il1403	0	0		
208435	Streptococcus agalactiae 2603V/R	0	1		
486410	Streptococcus dysgalactiae subsp. equisimilis GGS_124	1	1		
552526	Streptococcus equi subsp. zooepidemicus MGCS10565	1	1		
637909	Streptococcus gallolyticus UCN34	1	3		
467705	Streptococcus gordonii str. Challis substr. CH1	1	1		
365659	Streptococcus mitis B6	0	1		
210007	Streptococcus mutans UA159	2	2		
170187	Streptococcus pneumoniae TIGR4	0	1		
160490	Streptococcus pyogenes M1 GAS	1	1		
388919	Streptococcus sanguinis SK36	1	1		
391295	Streptococcus suis 05ZYH33	0	1		
299768	Streptococcus thermophilus CNRZ1066	0	1		
218495	Streptococcus uberis 0140J	0	1		
	**Lactobacillaceae**	5	6	15	3
272621	Lactobacillus acidophilus NCFM	0	0		
387344	Lactobacillus brevis ATCC 367	1	1		
321967	Lactobacillus casei ATCC 334	0	0		
321956	Lactobacillus delbrueckii subsp. bulgaricus ATCC BAA-365	0	0		
334390	Lactobacillus fermentum IFO 3956	0	0		
405566	Lactobacillus helveticus DPC 4571	0	0		
257314	Lactobacillus johnsonii NCC 533	0	0		
220668	Lactobacillus plantarum WCFS1	1	2		
557433	Lactobacillus reuteri JCM 1112	1	1		
568703	Lactobacillus rhamnosus GG	0	0		
314315	Lactobacillus sakei subsp. sakei 23K	1	1		
362948	Lactobacillus salivarius subsp. salivarius UCC118	0	0		
203120	Leuconostoc mesenteroides subsp. mesenteroides ATCC 8293	1	1		
203123	Oenococcus oeni PSU-1	0	0		
278197	Pediococcus pentosaceus ATCC 25745	0	0		
	**Enterococcaceae**	4	4	2	2

226185	Enterococcus faecalis V583	2	2		
333849	Enterococcus faecium DO	2	2		
	**Listeriaceae**	4	4	4	1
272626	Listeria innocua Clip11262	1	1		
169963	Listeria monocytogenes EGD-e	1	1		
683837	Listeria seeligeri serovar 1/2b str. SLCC3954	1	1		
386043	Listeria welshimeri serovar 6b str. SLCC5334	1	1		
**Class**	**Clostridia**	29	48	**32**	9
	**Clostridia-1**	8	22	8	4
272562	Clostridium acetobutylicum ATCC 824	0	2		
447214	Clostridium butyricum 5521	1	1		
290402	Clostridium beijerincki NCIMB 8052	3	5		
413999	Clostridium botulinum A str. ATCC 3502	3	7		
431943	Clostridium kluyveri DSM 555	1	3		
386415	Clostridium novyi NT	0	1		
195103	Clostridium perfringens ATCC 13124	0	2		
212717	Clostridium tetani E88	0	1		
	**Clostridia-2**	3	5	3	2
445973	Clostridium bartlettii DSM 16795	0	0		
272563	Clostridium difficile 630	3	5		
500633	Clostridium hiranonis DSM 13275	0	0		
	**Clostridia-3**	16	16	14	2
483218	Bacteroides pectinophilus ATCC 43243	1	1		
537007	Blautia hansenii DSM 20583	1	1		
478749	Bryantella formatexigens DSM 14469	1	1		
457421	Clostridiales bacterium 1_7_47_FAA	1	1		
411902	Clostridium bolteae ATCC BAA-613	2	2		
500632	Clostridium nexile DSM 1787	1	1		
411468	Clostridium scindens ATCC 35704	1	1		
411461	Dorea formicigenerans ATCC 27755	1	1		
411462	Dorea longicatena DSM 13814	1	1		
515620	Eubacterium eligens ATCC 27750	2	2		
515619	Eubacterium rectale ATCC 33656	1	1		
536231	Roseburia intestinalis L1-82	1	1		
411470	Ruminococcus gnavus ATCC 29149	1	1		
471875	Ruminococcus lactaris ATCC 29176	1	1		
	**Thermoanaerobacterales**	2	5	7	1
399726	Thermoanaerobacter ethanolicus X514	0	1		
580331	Thermoanaerobacter italicus Ab9	0	1		
273068	Thermoanaerobacter tengcongensis MB4	0	1		
351627	Caldicellulosiruptor saccharolyticus DSM 8903	1	1		
246194	Carboxydothermus hydrogenoformans Z-2901	0	0		
521460	Anaerocellum thermophilum DSM 6725	1	1		
264732	Moorella thermoacetica ATCC 39073	0	0		
**Phylum**	**Actinobacteria**	**106**	**120**	**48**	**31**
	**Streptomycetaceae**	30	31	4	5
227882	Streptomyces avermitilis MA-4680	11	11		
100226	Streptomyces coelicolor A3(2)	5	5		
455632	Streptomyces griseus subsp. griseus NBRC 13350	8	8		
680198	Streptomyces scabiei 87.22	6	7		
	**Mycobacteriaceae**	20	29	9	6
561007	Mycobacterium abscessus ATCC 19977	5	5		
243243	Mycobacterium avium 104	1	1		
350054	Mycobacterium flavescens PYR-GCK (Mycobacterium gilvum)	2	2		
272631	Mycobacterium leprae TN	0	0		
216594	Mycobacterium marinum M	1	1		
246196	Mycobacterium smegmatis str. MC2 155	7	16		

164757	Mycobacterium sp. JLS	2	2		
83332	Mycobacterium tuberculosis H37Rv	0	0		
350058	Mycobacterium vanbaalenii PYR-1	2	2		
	**Corynebacteriaceae**	3	3	8	1
553204	Corynebacterium amycolatum SK46	0	0		
548476	Corynebacterium aurimucosum ATCC 700975	1	1		
257309	Corynebacterium diphtheriae NCTC 13129	1	1		
196164	Corynebacterium efficiens YS-314	0	0		
196627	Corynebacterium glutamicum ATCC 13032	1	1		
306537	Corynebacterium jeikeium K411	0	0		
645127	Corynebacterium kroppenstedtii DSM 44385	0	0		
504474	Corynebacterium urealyticum DSM 7109	0	0		
	**Micrococcineae**	19	20	14	7
290340	Arthrobacter aurescens TC1	2	2		
452863	Arthrobacter chlorophenolicus A6	2	2		
290399	Arthrobacter sp. FB24	2	2		
471853	Beutenbergia cavernae DSM 12333	3	3		
446465	Brachybacterium faecium DSM 4810	1	1		
321955	Brevibacterium linens BL2	2	2		
443906	Clavibacter michiganensis subsp. michiganensis NCPPB 382	1	1		
313589	Janibacter sp. HTCC2649	3	3		
471856	Jonesia denitrificans DSM 20603	1	1		
378753	Kocuria rhizophila DC2201	1	1		
478801	Kytococcus sedentarius DSM 20547	0	0		
281090	Leifsonia xyli subsp. xyli str. CTCB07	1	1		
288705	Renibacterium salmoninarum ATCC 33209	0	1		
203267	Tropheryma whipplei str. Twist	0	0		
	**Nocardiaceae**	19	21	4	7
247156	Nocardia farcinica IFM 10152	4	5		
234621	Rhodococcus erythropolis PR4	4	4		
101510	Rhodococcus sp. RHA1	6	7		
632772	Rhodococcus opacus B4	5	5		
	**Frankineae/Propionibacterineae/Pseudonocardiaceae**	15	16	9	5
446462	Actinosynnema mirum DSM 43827	2	2		
405948	Saccharopolyspora erythraea NRRL 2338	5	5		
471857	Saccharomonospora viridis DSM 43017	1	1		
479431	Nakamurella multipartita DSM 44233	4	4		
298653	Frankia sp. EAN1pec	0	0		
106370	Frankia sp. CcI3	0	0		
351607	Acidothermus cellulolyticus 11B	0	0		
267747	Propionibacterium acnes KPA171202	1	1		
196162	Nocardioides sp. JS614	2	3		
**Phylum**	**Bacteroidetes**	**23**	**27**	**38**	**8**
	**Bacteroidaceae**	1	1	11	1
537012	Bacteroides cellulosilyticus DSM 14838	0	0		
547042	Bacteroides coprophilus DSM 18228	0	0		
483217	Bacteroides dorei DSM 17855	0	0		
483216	Bacteroides eggerthii DSM 20697	0	0		
272559	Bacteroides fragilis NCTC 9343	0	0		
411476	Bacteroides ovatus ATCC 8483	1	1		
484018	Bacteroides plebeius DSM 17135	0	0		
449673	Bacteroides stercoris ATCC 43183	0	0		
226186	Bacteroides thetaiotaomicron VPI-5482	0	0		
411479	Bacteroides uniformis ATCC 8492	0	0		
435590	Bacteroides vulgatus ATCC 8482	0	0		
	**Flavobacteria**	7	8	19	2
331104	Blattabacterium sp. (Blattella germanica) str. Bge	0	0		

595499	Candidatus Sulcia muelleri SMDSEM	0	0		
521097	Capnocytophaga ochracea DSM 7271	0	0		
313590	Cellulophaga sp. MED134	0	0		
216432	Croceibacter atlanticus HTCC2559	0	0		
391598	Flavobacteria bacterium BAL38	1	1		
156586	Flavobacteria bacterium BBFL7	0	0		
531844	Flavobacteriaceae bacterium 3519-10	0	0		
391603	Flavobacteriales bacterium ALC-1	0	0		
313603	Flavobacteriales bacterium HTCC2170	1	1		
376686	Flavobacterium johnsoniae UW101	3	4		
402612	Flavobacterium psychrophilum JIP02/86	0	0		
411154	Gramella forsetii KT0803	0	0		
391587	Kordia algicida OT-1	0	0		
398720	Leeuwenhoekiella blandensis MED217	0	0		
313594	Polaribacter irgensii 23-P	1	1		
313595	Psychroflexus torquis ATCC 700755	0	0		
313596	Robiginitalea biformata HTCC2501	0	0		
313598	Tenacibaculum sp. MED152	1	1		
	**Sphingobacteria**	15	18	8	2
388413	Algoriphagus sp. PR1	0	0		
485918	Chitinophaga pinensis DSM 2588	7	9		
269798	Cytophaga hutchinsonii ATCC 33406	0	0		
471854	Dyadobacter fermentans DSM 18053	4	5		
313606	Microscilla marina ATCC 23134	1	1		
485917	Pedobacter heparinus DSM 2366	1	1		
391596	Pedobacter sp. BAL39	2	2		
309807	Salinibacter ruber DSM 13855	0	0		
**Phylum**	**Deinococcus-Thermus**	**1**	**5**	**5**	**1**
319795	Deinococcus geothermalis DSM 11300	0	1		
546414	Deinococcus deserti VCD115	1	2		
243230	Deinococcus radiodurans R1	0	2		
498848	Thermus aquaticus Y51MC23	0	0		
262724	Thermus thermophilus HB27	0	0		
**Phylum**	**Cyanobacteria**	**2**	**3**	**14**	**1**
32049	Synechococcus sp. PCC 7002	0	0		
1148	Synechocystis sp. PCC 6803	1	1		
43989	Cyanothece sp. ATCC 51142	0	0		
41431	Cyanothece sp. PCC 8801	0	0		
395961	Cyanothece sp. PCC 7425	0	0		
449447	Microcystis aeruginosa NIES-843	0	0		
103690	Nostoc sp. PCC 7120	0	1		
203124	Trichodesmium erythraeum IMS101	0	0		
1140	Synechococcus elongatus PCC 7942	1	1		
74547	Prochlorococcus marinus str. MIT 9313	0	0		
321327	Synechococcus sp. JA-3-3Ab	0	0		
84588	Synechococcus sp. WH 8102	0	0		
251221	Gloeobacter violaceus PCC 7421	0	0		
197221	Thermosynechococcus elongatus BP-1	0	0		
	**Total number of MocR-TFs with reconstructed regulons:**	**825**			
	**Total number of MocR-TFs in the analyzed genomes:**		**974**		
	**Total number of analyzed genomes:**			**390**	
	**Total number of reconstructed MocR-TF regulogs:**				**284**

* Number of MocR subfamily regulators analyzed in this work

** Total number of MocR subfamily regulators according to P2TF database (http://www.p2tf.org/)

[1] Taxonomic collections are according to the standartized genomic collections in the RegPrecise database.

[2] Genomes containing studied MocR-TFs are highlighted by yellow

Table S1b. Studied MocR-TF regulators and gene content of reconstructed regulogs .

Orthologous group [1]	Regulog	Genome	TF locus tag	Regulated genes [2]
Sbal_3352	Ent638_1108 - Enterobacteriales	Enterobacter sp. 638	Ent638_1108	COG456 (RimI)
Sbal_3352	Sbal_3352 - Shewanellaceae	Shewanella baltica OS155	Sbal_3352	COG456 (RimI), TF
Sbal_3352	Sbal_3352 - Shewanellaceae	Shewanella putrefaciens CN-32	Sputcn32_3003	COG456 (RimI), TF
Sbal_3352	Sbal_3352 - Shewanellaceae	Shewanella sp W3-18-1	Sputw3181_0946	COG456 (RimI), TF
AHA_3778	AHA_3778 - Psychromonadaceae/Aeromonadale	Aeromonas hydrophila subsp. hydrophila ATCC 79	AHA_3778	COG607 (PspE), COG1764 (OsmC), COG1670 (RimL)
AHA_3778	AHA_3778 - Psychromonadaceae/Aeromonadale	Aeromonas salmonicida subsp. salmonicida A449	ASA_3803	COG607 (PspE), COG1764 (OsmC), COG1670 (RimL)
AHA_3778	EAM_2333 - Enterobacteriales	Erwinia amylovora ATCC 49946	EAM_2333	COG607 (PspE)
Spro_2017	Spro_2017 - Enterobacteriales	Serratia proteamaculans 568	Spro_2017	COG456 (RimI), COG1670 (RimL)
PdxR (Proteobacteria)	PdxR - Rhodospirillales	Rhodospirillum rubrum ATCC 11170	Rru_A1143	COG2240 (PdxK), COG4992 (ArgD), COG3135 (BenE), COG156 (BioF), COG325, COG112 (GlyA)
PdxR (Proteobacteria)	PdxR - Rhizobiales	Mesorhizobium sp. BNC1	Meso_4362	TF
PdxR (Proteobacteria)	PdxR - Rhizobiales	Rhodopseudomonas palustris CGA009	RPA2797	COG2240 (PdxK), COG3135 (BenE), COG325, COG112 (GlyA)
PdxR (Proteobacteria)	PdxR - Alcaligenaceae	Bordetella avium 197N	BAV1500	COG3135 (BenE)
PdxR (Proteobacteria)	PdxR - Alcaligenaceae	Bordetella bronchiseptica RB50	BB0707	COG3135 (BenE), COG112 (GlyA)
PdxR (Proteobacteria)	PdxR - Comamonadaceae	Comamonas testosteroni KF-1	CtesDRAFT_4060	COG4992 (ArgD), COG3135 (BenE), COG325
PdxR (Proteobacteria)	PdxR - Comamonadaceae	Delftia acidovorans SPH-1	Daci_3711	COG4992 (ArgD), COG3135 (BenE), COG325, COG112 (GlyA)
PdxR (Proteobacteria)	PdxR - Various betaproteobacteria	Thauera sp. MZ1T	Tmz1t_0986	COG4992 (ArgD), COG3135 (BenE), COG325, COG112 (GlyA), COG518 (GuaA)
PdxR (Proteobacteria)	PdxR - Enterobacteriales	Erwinia carotovora subsp. atroseptica SCRI1043	ECA4048	COG456 (RimI), COG325, COG112 (GlyA)
PdxR (Proteobacteria)	PdxR - Pseudomonadaceae	Pseudomonas aeruginosa PAO1	PA4165	COG456 (RimI)
PdxR (Proteobacteria)	PdxR - Pseudomonadaceae	Pseudomonas fluorescens Pf-5	PFL_0754	COG3135 (BenE), COG456 (RimI)
PdxW (Proteobacteria)	PdxW - Alcaligenaceae	Bordetella petrii DSM 12804	Bpet2921	COG1670 (RimL)
PdxW (Proteobacteria)	PdxW - Alcaligenaceae	Bordetella avium 197N	BAV1623	COG1670 (RimL)
PdxW (Proteobacteria)	PdxW - Alcaligenaceae	Bordetella bronchiseptica RB50	BB2428	COG1670 (RimL)
PdxW (Proteobacteria)	PdxW - Burkholderia	Burkholderia cepacia AMMD	Bamb_3950	COG1670 (RimL)
PdxW (Proteobacteria)	PdxW - Burkholderia	Burkholderia cepacia AMMD	Bamb_5126	COG456 (RimI)
PdxW (Proteobacteria)	PdxW - Burkholderia	Burkholderia mallei ATCC 23344	BMAA1398	COG3467 (PdxO)
PdxW (Proteobacteria)	PdxW - Burkholderia	Burkholderia pseudomallei K96243	BPSS0726	COG3467 (PdxO)
PdxW (Proteobacteria)	PdxW - Burkholderia	Burkholderia glumae BGR1	bglu_2g17640	COG1670 (RimL)
PdxW (Proteobacteria)	PdxW - Burkholderia	Burkholderia vietnamiensis G4	Bcep1808_5059	COG1670 (RimL)
PdxW (Proteobacteria)	PdxW - Burkholderia	Burkholderia xenovorans LB400	Bxe_B0246	COG456 (RimI), COG1670 (RimL)
PdxW (Proteobacteria)	PdxW - Burkholderia	Burkholderia sp. 383	Bcep18194_B2202	COG456 (RimI)
PdxW (Proteobacteria)	PdxW - Burkholderia	Burkholderia sp. 383	Bcep18194_B1330	COG1670 (RimL)
PdxW (Proteobacteria)	PdxW - Burkholderia	Burkholderia phymatum STM815	Bphy_4112	COG456 (RimI), COG1670 (RimL)
PdxW (Proteobacteria)	PdxW - Comamonadaceae	Variovorax paradoxus S110	Vapar_3858	COG1670 (RimL)
PdxW (Proteobacteria)	PdxW - Comamonadaceae	Delftia acidovorans SPH-1	Daci_2817	COG1670 (RimL)
PdxW (Proteobacteria)	PdxW - Ralstonia	Ralstonia pickettii 12J	Rpic_3403	COG3467 (PdxO), COG1670 (RimL)
PdxW (Proteobacteria)	PdxW - Ralstonia	Ralstonia solanacearum GMI1000	RSc3119 (RS00574)	COG3467 (PdxO)
PdxW (Proteobacteria)	PdxW - Various betaproteobacteria	Thauera sp. MZ1T	Tmz1t_1910	COG3467 (PdxO)
PdxW (Proteobacteria)	PdxW - Enterobacteriales	Enterobacter sp. 638	Ent638_0937	COG1670 (RimL)
PdxW (Proteobacteria)	PdxW - Enterobacteriales	Klebsiella pneumoniae subsp. pneumoniae MGH 7	KPN_00438	COG1670 (RimL)
PdxW2 (Oceanospirillales)	PdxW2 - Oceanospirillales/Alteromonadales	Chromohalobacter salexigens DSM 3043	Csal_1152	COG456 (RimI), COG1670 (RimL), COG2808 (PdxO2)
CV4096	CV4096 - Various betaproteobacteria	Chromobacterium violaceum ATCC 12472	CV4096	COG1670 (RimL), COG607 (PspE)
GabR (Proteobacteria)	GabR - Alcaligenaceae	Bordetella avium 197N	BAV2547	COG160 (GabT), COG1012 (GabD)
GabR (Proteobacteria)	GabR - Alcaligenaceae	Bordetella bronchiseptica RB50	BB1808	COG160 (GabT), COG1012 (GabD)
GabR (Proteobacteria)	GabR - Alcaligenaceae	Bordetella petrii DSM 12804	Bpet3402	COG160 (GabT), COG1012 (GabD)
GabR (Proteobacteria)	GabR - Alcaligenaceae	Bordetella avium 197N	BAV0456	COG160 (GabT), COG624 (ArgE), COG2334
GabR (Proteobacteria)	GabR - Alcaligenaceae	Bordetella avium 197N	BAV2194	COG160 (GabT), COG747 (DdpA), COG601 (DppB), COG1173 (DppC), COG1123 (DppD)
GabR (Proteobacteria)	GabR - Alcaligenaceae	Bordetella bronchiseptica RB50	BB1885	COG160 (GabT), COG747 (DdpA), COG601 (DppB), COG1173 (DppC), COG1123 (DppD)
GabR (Proteobacteria)	GabR - Alcaligenaceae	Bordetella petrii DSM 12804	Bpet3078	COG160 (GabT), COG747 (DdpA), COG601 (DppB), COG1173 (DppC), COG1123 (DppD)
GabR (Proteobacteria)	GabR - Burkholderia	Burkholderia cepacia AMMD	Bamb_4710	COG160 (GabT), COG1012 (GabD)
GabR (Proteobacteria)	GabR - Burkholderia	Burkholderia mallei ATCC 23344	BMAA1478.1	COG160 (GabT), COG1012 (GabD)
GabR (Proteobacteria)	GabR - Burkholderia	Burkholderia phymatum STM815	Bphy_4619	COG160 (GabT), COG1012 (GabD)
GabR (Proteobacteria)	GabR - Burkholderia	Burkholderia phymatum STM815	Bphy_4852	COG160 (GabT), COG1012 (GabD)
GabR (Proteobacteria)	GabR - Burkholderia	Burkholderia sp. 383	Bcep18194_B0281	COG160 (GabT), COG1012 (GabD)
GabR (Proteobacteria)	GabR - Burkholderia	Burkholderia vietnamiensis G4	Bcep1808_3588	COG160 (GabT), COG1012 (GabD)
GabR (Proteobacteria)	GabR - Burkholderia	Burkholderia xenovorans LB400	Bxe_B0835	COG160 (GabT), COG1012 (GabD)
GabR (Proteobacteria)	GabR - Burkholderia	Burkholderia cepacia AMMD	Bamb_3295	COG3842 (GbtA), COG1176 (GbtB), COG1177 (GbtC), COG687 (GbtD)
GabR (Proteobacteria)	GabR - Burkholderia	Burkholderia mallei ATCC 23344	BMAA0430	COG3842 (GbtA), COG1176 (GbtB), COG687 (GbtD)
GabR (Proteobacteria)	GabR - Burkholderia	Burkholderia phymatum STM815	Bphy_4320	COG3842 (GbtA), COG1176 (GbtB), COG1177 (GbtC), COG687 (GbtD)
GabR (Proteobacteria)	GabR - Burkholderia	Burkholderia sp. 383	Bcep18194_B2171	COG3842 (GbtA), COG1176 (GbtB), COG1177 (GbtC), COG687 (GbtD)
GabR (Proteobacteria)	GabR - Burkholderia	Burkholderia vietnamiensis G4	Bcep1808_4394	COG3842 (GbtA), COG1176 (GbtB), COG1177 (GbtC), COG687 (GbtD)
GabR (Proteobacteria)	GabR - Burkholderia	Burkholderia xenovorans LB400	Bxe_B2934	COG3842 (GbtA), COG1176 (GbtB), COG1177 (GbtC), COG687 (GbtD)
GabR (Proteobacteria)	GabR - Burkholderia	Burkholderia pseudomallei K96243	BPSS1739	COG3842 (GbtA), COG1176 (GbtB), COG1177 (GbtC), COG687 (GbtD)
GabR (Proteobacteria)	GabR - Burkholderia	Burkholderia pseudomallei K96243	BPSS0282	COG160 (GabT), COG1012 (GabD)
GabR (Proteobacteria)	GabR - Burkholderia	Burkholderia glumae BGR1	bglu_2g03390	COG160 (GabT), COG1012 (GabD)
GabR (Proteobacteria)	GabR - Comamonadaceae	Delftia acidovorans SPH-1	Daci_5048	COG160 (GabT), COG1012 (GabD)
GabR (Proteobacteria)	GabR - Comamonadaceae	Rhodoferax ferrireducens DSM 15236	Rfer_0348	COG161 (GatP), COG174 (PuuA), COG2071 (PuuD), COG3842 (PuuL),COG1176 (PuuM),COG1177 (PuuN),COG687 (PuuO)
GabR (Proteobacteria)	GabR - Comamonadaceae	Polaromonas naphthalenivorans CJ2	Pnap_2866	COG161 (GatP), COG174 (PuuA), COG2071 (PuuD), COG3842 (PuuL),COG1176 (PuuM),COG1177 (PuuN),COG687 (PuuO)
GabR (Proteobacteria)	GabR - Ralstonia	Cupriavidus taiwanensis	RALTA_A1416	COG160 (GabT), COG1012 (GabD), COG747 (DdpA), COG601 (DppB), COG1173 (DppC), COG1123 (DppD), COG624 (ArgE)
GabR (Proteobacteria)	GabR - Ralstonia	Ralstonia eutropha H16	H16_B0980	COG160 (GabT), COG1012 (GabD)
GabR (Proteobacteria)	GabR - Ralstonia	Ralstonia metallidurans CH34	Rmet_1609	COG160 (GabT), COG747 (DdpA), COG601 (DppB), COG1173 (DppC), COG1123 (DppD), COG624 (ArgE)
GabR (Proteobacteria)	GabR - Ralstonia	Ralstonia pickettii 12J	Rpic_3720	COG160 (GabT), COG1012 (GabD), COG3842 (GbtA), COG1176 (GbtB), COG1177 (GbtC), COG687 (GbtD)
GabR (Proteobacteria)	GabR - Ralstonia	Ralstonia solanacearum GMI1000	RSc0030 (RS01853)	COG160 (GabT), COG1012 (GabD), COG3842 (GbtA), COG1176 (GbtB), COG1177 (GbtC), COG687 (GbtD)
GabR (Proteobacteria)	GabR - Various betaproteobacteria	Thauera sp. MZ1T	Tmz1t_0423	COG3842 (GbtA), COG1176 (GbtB), COG1177 (GbtC), COG687 (GbtD), COG160 (GabT), COG1012 (GabD, PutA)
GabR (Proteobacteria)	GabR - Enterobacteriales	Erwinia carotovora subsp. atroseptica SCRI1043	ECA2052	COG160 (GabT), COG1012 (GabD), COG1176 (GbtB), COG1177 (GbtC), COG687 (GbtD)
GabR (Proteobacteria)	GabR - Enterobacteriales	Serratia proteamaculans 568	Spro_4307	COG160 (GabT), COG1012 (GabD)
GabR (Proteobacteria)	GabR - Enterobacteriales	Erwinia amylovora ATCC 49946	EAM_2304	COG160 (GabT), COG1176 (GbtB), COG1177 (GbtC), COG687 (GbtD)
GabR (Proteobacteria)	GabR - Enterobacteriales	Klebsiella pneumoniae subsp. pneumoniae MGH 7	KPN_03826	COG160 (GabT)
GabR (Proteobacteria)	GabR - Enterobacteriales	Yersinia pestis KIM 10	y1389	COG160 (GabT), COG1176 (GbtB), COG1177 (GbtC), COG687 (GbtD)
GabR (Proteobacteria)	GabR - Moraxellaceae	Acinetobacter baumannii AB0057	AB57_3729	COG160 (GabT), COG1012 (GabD)
GabR (Proteobacteria)	GabR - Moraxellaceae	Acinetobacter sp. ADP1	ACIAD3447	COG160 (GabT), COG1012 (GabD), COG1113 (GabP)
GabR (Proteobacteria)	GabR - Psychromonadaceae/Aeromonadales	Psychromonas ingrahamii 37	Ping_0569	TF
PdxW2 (Proteobacteria)	PdxW2 - Burkholderia	Burkholderia cepacia AMMD	Bamb_4617	COG2808 (PdxO2)
PdxW2 (Proteobacteria)	PdxW2 - Burkholderia	Burkholderia glumae BGR1	bglu_2g19620	COG2808 (PdxO2)
PdxW2 (Proteobacteria)	PdxW2 - Burkholderia	Burkholderia mallei ATCC 23344	BMA3061	COG2808 (PdxO2)
PdxW2 (Proteobacteria)	PdxW2 - Burkholderia	Burkholderia phymatum STM815	Bphy_5851	COG2808 (PdxO2)
PdxW2 (Proteobacteria)	PdxW2 - Burkholderia	Burkholderia pseudomallei K96243	BPSL1310	COG2808 (PdxO2)
PdxW2 (Proteobacteria)	PdxW2 - Burkholderia	Burkholderia sp. 383	Bcep18194_B0395	COG2808 (PdxO2)
PdxW2 (Proteobacteria)	PdxW2 - Moraxellaceae	Acinetobacter baumannii AB0057	AB57_1649	COG1670 (RimL), COG2808 (PdxO2)
PdxW2 (Proteobacteria)	PdxW2 - Pseudomonadaceae	Pseudomonas aeruginosa PAO1	PA5431	COG456 (RimI), COG1670 (RimL)
PdxW2 (Proteobacteria)	PdxW2 - Pseudomonadaceae	Pseudomonas entomophila L48	PSEEN5491	COG456 (RimI), COG1670 (RimL), COG2808 (PdxO2)
PdxW2 (Proteobacteria)	PdxW2 - Pseudomonadaceae	Pseudomonas fluorescens Pf-5	PFL_6148	COG456 (RimI), COG1670 (RimL), COG2808 (PdxO2)
PdxW2 (Proteobacteria)	PdxW2 - Pseudomonadaceae	Pseudomonas mendocina ymp	Pmen_0130	COG456 (RimI), COG1670 (RimL), COG2808 (PdxO2)
PdxW2 (Proteobacteria)	PdxW2 - Pseudomonadaceae	Pseudomonas putida KT2440	PP5342	COG456 (RimI), COG1670 (RimL), COG2808 (PdxO2)
PdxW2 (Proteobacteria)	PdxW2 - Pseudomonadaceae	Pseudomonas syringae pv. tomato str. DC3000	PSPTO5504	COG456 (RimI), COG1670 (RimL), COG2808 (PdxO2)
PdxW (Gammaproteobacteria)	PdxW - Oceanospirillales/Alteromonadales	Marinobacter aqueolei	Maqu_2951	COG3467 (PdxO)
PdxW (Gammaproteobacteria)	PdxW - Oceanospirillales/Alteromonadales	Oceanospirillum sp. MED92	MED92_15483	COG3467 (PdxO)
PdxW (Gammaproteobacteria)	PdxW - Vibrionales	Vibrio harveyi ATCC BAA-1116	VIBHAR_05334	COG3467 (PdxO)
PdxW (Gammaproteobacteria)	PdxW - Vibrionales	Vibrio angustum S14	VAS14_14939	COG3467 (PdxO)
PdxW (Gammaproteobacteria)	PdxW - Vibrionales	Vibrio parahaemolyticus RIMD 2210633	VPA1237	COG3467 (PdxO)
PdxW (Gammaproteobacteria)	PdxW - Vibrionales	Vibrio shilonii AK1	VSAK1_18719	COG3467 (PdxO)
PdxW (Gammaproteobacteria)	PdxW - Vibrionales	Vibrio splendidus LGP32	VS_II0337	COG3467 (PdxO)
PdxW (Gammaproteobacteria)	PdxW - Vibrionales	Vibrio vulnificus CMCP6	VV2_0776	COG3467 (PdxO)
PdxQ (Bacillales)	PdxQ - Bacillales	Bacillus amyloliquefaciens FZB42	RBAM_005770	COG2808 (PdxO2), COG329 (DapA)
PdxQ (Bacillales)	PdxQ - Bacillales	Geobacillus kaustophilus HTA426	GK0738	COG2808 (PdxO2), COG3382, COG1280 (RhtB)
PdxW (Desulfuromonadales)	PdxW - Desulfuromonadales	Geobacter sp. M21	GM21_0205	PF01243 (PdxO3), TF
PdxR (Bacilli)	PdxR - Pasteurellales	Actinobacillus pleuropneumoniae serovar 7 str. AP	APP7_0615	COG214 (PdxS), COG311 (PdxT)
PdxR (Bacilli)	PdxR - Pasteurellales	Haemophilus ducreyi 35000HP	HD1595	COG214 (PdxS), COG311 (PdxT)
PdxR (Bacilli)	PdxR - Pasteurellales	Pasteurella multocida subsp. multocida str. Pm70	PM1231	COG214 (PdxS), COG311 (PdxT)
PdxR (Bacilli)	PdxR - Bacillales	Bacillus clausii KSM-K16	ABC0450	COG214 (PdxS), COG311 (PdxT)
PdxR (Bacilli)	PdxR - Bacillales	Oceanobacillus iheyensis HTE831	OB2688	COG214 (PdxS), COG311 (PdxT)
PdxR (Bacilli)	PdxR - Staphylococcaceae	Macrococcus caseolyticus JCSC5402	MCCL_1894	COG214 (PdxS), COG311 (PdxT)
PdxR (Bacilli)	PdxR - Staphylococcaceae	Staphylococcus aureus subsp. aureus N315	SA0476	COG214 (PdxS), COG311 (PdxT)

PdxR (Bacilli)	PdxR - Staphylococcaceae	Staphylococcus carnosus subsp. carnosus TM300	Sca_0172	COG214 (PdxS), COG311 (PdxT)
PdxR (Bacilli)	PdxR - Staphylococcaceae	Staphylococcus epidermidis ATCC 12228	SE2263	COG214 (PdxS), COG311 (PdxT)
PdxR (Bacilli)	PdxR - Staphylococcaceae	Staphylococcus haemolyticus JCSC1435	SH2491	COG214 (PdxS), COG311 (PdxT)
PdxR (Bacilli)	PdxR - Staphylococcaceae	Staphylococcus saprophyticus subsp. saprophyticu	SSP2238	COG214 (PdxS), COG311 (PdxT)
YhdI/YdeL	YhdI/YdeL - Staphylococcaceae	Staphylococcus saprophyticus subsp. saprophyticu	SSP1621	COG2962 (RarD)
YhdI/YdeL	YhdI/YdeL - Bacillales	Bacillus cereus ATCC 14579	BC0583	COG2962 (RarD)
YhdI/YdeL	YhdI/YdeL - Bacillales	Bacillus amyloliquefaciens FZB42	RBAM_005860	COG2962 (RarD)
YhdI/YdeL	YhdI/YdeL - Bacillales	Bacillus subtilis subsp. subtilis str. 168	BSU05240	COG2962 (RarD)
YhdI/YdeL	YhdI/YdeL - Bacillales	Bacillus cereus ATCC 14579	BC2068	COG456 (RimI)
YhdI/YdeL	YhdI/YdeL - Bacillales	Bacillus licheniformis ATCC 14580	BLi01016	COG456 (RimI)
YhdI/YdeL	YhdI/YdeL - Bacillales	Bacillus subtilis subsp. subtilis str. 168	BSU09480	COG456 (RimI)
PdxR (Clostridia)	PdxR - Clostridia-3	Bacteroides pectinophilus ATCC 43243	BACPEC_02643	COG4720 (PdxU)
PdxR (Clostridia)	PdxR - Clostridia-3	Blautia hansenii DSM 20583	BLAHAN_03146	COG4720 (PdxU)
PdxR (Clostridia)	PdxR - Clostridia-3	Bryantella formatexigens DSM 14469	BRYFOR_00578	COG4720 (PdxU)
PdxR (Clostridia)	PdxR - Clostridia-3	Clostridiales bacterium 1_7_47_FAA	Cbac1_01010001672	COG4720 (PdxU)*2, COG2240 (PdxK)
PdxR (Clostridia)	PdxR - Clostridia-3	Clostridium bolteae ATCC BAA-613	CLOBOL_06805	COG4720 (PdxU)*2, COG2240 (PdxK)
PdxR (Clostridia)	PdxR - Clostridia-3	Clostridium nexile DSM 1787	CLONEX_00239	COG4720 (PdxU)*2
PdxR (Clostridia)	PdxR - Clostridia-3	Clostridium scindens ATCC 35704	CLOSCI_01235	COG4720 (PdxU)*2, COG2240 (PdxK)
PdxR (Clostridia)	PdxR - Clostridia-3	Dorea formicigenerans ATCC 27755	DORFOR_00757	COG214 (PdxS), COG311 (PdxT)
PdxR (Clostridia)	PdxR - Clostridia-3	Dorea longicatena DSM 13814	DORLON_02845	COG214 (PdxS), COG311 (PdxT), COG4720 (PdxU)*2
PdxR (Clostridia)	PdxR - Clostridia-3	Eubacterium eligens ATCC 27750	EUBELI_20396	COG214 (PdxS), COG311 (PdxT)
PdxR (Clostridia)	PdxR - Clostridia-3	Eubacterium eligens ATCC 27750	EUBELI_20570	
PdxR (Clostridia)	PdxR - Clostridia-3	Eubacterium rectale ATCC 33656	EUBREC_2949	COG4720 (PdxU)
PdxR (Clostridia)	PdxR - Clostridia-3	Roseburia intestinalis L1-82	ROSINTL182_03113	COG214 (PdxS), COG311 (PdxT), hypothetical protein
PdxR (Clostridia)	PdxR - Clostridia-3	Ruminococcus gnavus ATCC 29149	RUMGNA_00641	COG214 (PdxS), COG311 (PdxT), COG4720 (PdxU)
PdxR (Clostridia)	PdxR - Clostridia-3	Ruminococcus lactaris ATCC 29176	RUMLAC_01873	COG214 (PdxS), COG311 (PdxT), COG4720 (PdxU)
GabR (Bacilli)	GabR - Bacillales	Bacillus amyloliquefaciens FZB42	RBAM_004140	COG160 (GabT), COG1012 (GabD)
GabR (Bacilli)	GabR - Bacillales	Bacillus licheniformis ATCC 14580	BLi00473	COG160 (GabT), COG1012 (GabD), COG1113 (GabP)
GabR (Bacilli)	GabR - Bacillales	Bacillus pumilus SAFR-032	BPUM_0361	COG160 (GabT), COG1012 (GabD), COG1113 (GabP)
GabR (Bacilli)	GabR - Bacillales	Bacillus subtilis subsp. subtilis str. 168	BSU03890	COG160 (GabT), COG1012 (GabD)
GabR (Bacilli)	GabR - Staphylococcaceae	Staphylococcus saprophyticus subsp. saprophyticu	SSP0186	COG160 (GabT), COG1012 (GabD), COG1113 (GabP)
PdxQ (Deltaproteobacteria)	PdxQ - Desulfuromonadales	Desulfuromonas acetoxidans DSM 684	Dace_1191	COG3467 (PdxO), COG1280 (RhtB), COG456 (RimI)
PdxQ (Deltaproteobacteria)	PdxQ - Desulfovibrionales	Desulfovibrio vulgaris str. Miyazaki F	DvMF_3005	COG3467 (PdxO)
PdxQ (Deltaproteobacteria)	PdxQ - Desulfovibrionales	Desulfovibrio desulfuricans subsp. desulfuricans st	Ddes_1162	COG3467 (PdxO)
PdxQ (Deltaproteobacteria)	PdxQ - Clostridia-1	Clostridium beijerinckii NCIMB 8052	Cbei_2205	COG3467 (PdxO)
PdxQ (Deltaproteobacteria)	PdxQ - Clostridia-1	Clostridium botulinum A str. ATCC 3502	CBO0640	COG2832, COG3467 (PdxO), hypothetical protein
DVU2086	DVU2086 - Desulfovibrionales	Desulfovibrio vulgaris Hildenborough	DVU2086	COG3382
DVU2086	BH1940 - Bacillales	Bacillus halodurans C-125	BH1940	COG506 (PutA), COG697 (RhaT)
DVU2086	CD3353 - Clostridia-2	Clostridium difficile 630	CD3353	COG3382
PdxW (Bacillales)	PdxW - Bacillales	Bacillus halodurans C-125	BH0578	COG3467 (PdxO)
PdxQ (Proteobacteria)	PdxQ - Rhodobacterales	Paracoccus denitrificans PD1222	Pden_4833	COG2808 (PdxO2)
PdxQ (Proteobacteria)	PdxQ - Rhodospirillales	Rhodospirillum rubrum ATCC 11170	Rru_A1925	COG3467 (PdxO)
PdxQ (Proteobacteria)	PdxQ - Rhizobiales	Mesorhizobium loti MAFF303099	mlr6174	COG329 (DapA), COG2808 (PdxO2), COG3382, COG1280 (RhtB)*2
PdxQ (Proteobacteria)	PdxQ - Alcaligenaceae	Bordetella petrii DSM 12804	Bpet0243	COG2808 (PdxO2)
PdxQ (Proteobacteria)	PdxQ - Comamonadaceae	Delftia acidovorans SPH-1	Daci_2224	COG2808 (PdxO2)
PdxQ (Proteobacteria)	PdxQ - Comamonadaceae	Polaromonas naphthalenivorans CJ2	Pnap_4754	pseudogene
PdxQ (Proteobacteria)	PdxQ - Ralstonia	Ralstonia eutropha H16	H16_B1977	COG2808 (PdxO2), COG1280 (RhtB)
PdxQ (Proteobacteria)	PdxQ - Ralstonia	Cupriavidus taiwanensis	RALTA_B0828	COG2808 (PdxO2), COG1280 (RhtB)
PdxQ (Proteobacteria)	PdxQ - Oceanospirillales/Alteromonadales	Hahella chejuensis KCTC 2396	HCH_01884	COG3467 (PdxO)
TauR	TauR - Rhodobacterales	Jannaschia sp. CCS1	Jann_2850	COG28 (Xsc), COG280 (Pta), COG243 (BisC), COG437 (HybA), COG3302 (DmsC), COG161(BioA), COG665 (DadA)
TauR	TauR - Rhodobacterales	Loktanella vestfoldensis SKA53	SKA53_00604	COG243 (BisC), COG437 (HybA), COG3302 (DmsC), COG730 (TauE), COG282 (AckA), hypothetical protein, TF
TauR	TauR - Rhodobacterales	Rhodobacter sphaeroides 2.4.1	RSP_4017	PF07366 (TauX), COG665 (TauY), COG3090 (TauL), COG1593 (TauM), COG1638 (TauK), COG28 (Xsc), COG2855 (TauZ), COG280 (Pta)
TauR	TauR - Rhodobacterales	Roseobacter sp. MED193	MED193_12203	COG161 (Tpa), COG28 (Xsc), COG243 (BisC), COG437 (HybA), COG3302 (DmsC), COG2855 (TauZ), COG282 (AckA), COG730 (TauE)
TauR	TauR - Rhodobacterales	Roseovarius nubinhibens ISM	ISM_10755	COG161 (Tpa), COG28 (Xsc), COG280 (Pta), COG243 (BisC), COG437 (HybA), COG3302 (DmsC)
TauR	TauR - Rhodobacterales	Roseovarius sp. 217	ROS217_11941	COG161 (Tpa)*2, COG28 (Xsc), COG282 (AckA), COG243 (BisC), COG437 (HybA), COG3302 (DmsC), hypothetical protein
TauR	TauR - Rhodobacterales	Silicibacter TM1040	TM1040_0156	COG161 (Tpa), COG28 (Xsc), COG2855 (TauZ), COG730 (TauE), COG2128
TauR	TauR - Rhodobacterales	Silicibacter pomeroyi DSS-3	SPO3562	COG161 (Tpa), COG28 (Xsc), COG243 (BisC), COG437 (HybA), COG3302 (DmsC), COG280 (Pta)
TauR	TauR - Rhodobacterales	Paracoccus denitrificans PD1222	Pden_1648	PF07366 (TauX), COG665 (TauY), COG3090 (TauL), COG1593 (TauM), COG1638 (TauK), COG28 (Xsc), COG2855 (TauZ), COG280 (Pta)
TauR	TauR - Rhodobacterales	Paracoccus denitrificans PD1222	Pden_4274	COG161 (Tpa), TF
TauR	TauR - Rhizobiales	Sinorhizobium meliloti 1021	SMb21525	COG4521 (TauA), COG4525/1116 (TauB), COG600 (TauC), PF07366 (TauX), COG665 (TauY), COG28 (Xsc),COG2855 (TauZ), COG280 (Pta)
TauR	TauR - Burkholderia	Burkholderia phymatum STM815	Bphy_6226	COG4521 (TauA), COG4525/1116 (TauB), COG600 (TauC), PF07366 (TauX), COG665 (TauY), COG28 (Xsc), COG280 (Pta), COG1113 (GabP)
TauR	TauR - Burkholderia	Burkholderia sp. 383	Bcep18194_B1990	PF07366 (TauX), COG665 (TauY), COG28 (Xsc), COG280 (Pta), COG1012 (PutA), COG456 (RimI), COG730 (TauE), COG1113 (GabP)
TauR	TauR - Burkholderia	Burkholderia xenovorans LB400	Bxe_B0695	COG4521 (TauA), COG4525/1116 (TauB), COG600 (TauC), PF07366 (TauX), COG665 (TauY), COG28 (Xsc), COG280 (Pta), COG1113 (GabP), COG730 (TauE)
TauR	TauR - Comamonadaceae	Verminephrobacter eiseniae EF01-2	Veis_4287	COG4521 (TauA), COG4525/1116 (TauB), COG600 (TauC), COG665 (TauY), COG747 (OppA), COG601 (OppB), COG1173 (OppC), COG1123/COG444 (OppD/OppF)*2, COG1574, COG523
TauR	TauR - Comamonadaceae	Comamonas testosteroni KF-1	CtesDRAFT_2751	COG4521 (TauA), COG4525/1116 (TauB), COG600 (TauC), PF07366 (TauX), COG665 (TauY), COG1113 (GabP)
TauR	TauR - Comamonadaceae	Variovorax paradoxus S110	Vapar_3848	COG4521 (TauA), COG4525/1116 (TauB), COG600 (TauC), PF07366 (TauX), COG665 (TauY)
TauR	TauR - Comamonadaceae	Delftia acidovorans SPH-1	Daci_2022	PF07366 (TauX), COG665 (TauY), COG1113 (GabP)
TauR	TauR - Comamonadaceae	Delftia acidovorans SPH-1	Daci_5672	COG1574, COG523, COG747 (OppA), COG601 (OppB), COG1173 (OppC), COG1123/COG444 (OppD/OppF)*2
TauR	TauR - Ralstonia	Cupriavidus taiwanensis	RALTA_B1590	PF07366 (TauX), COG665 (TauY), COG4521 (TauA), COG4525/1116 (TauB)*2, COG600 (TauC)*2, COG3090 (TauL), COG1593 (TauM), COG1638 (TauK), COG2175 (TauD)
TauR	TauR - Ralstonia	Ralstonia eutropha H16	H16_B1891	PF07366 (TauX), COG665 (TauY), COG4521 (TauA), COG4525/1116 (TauB)*2, COG600 (TauC)*2, COG2175 (TauD)
TauR	TauR - Ralstonia	Ralstonia eutropha JMP134	Reut_B3752	PF07366 (TauX), COG665 (TauY), COG4521 (TauA), COG4525/1116 (TauB), COG600 (TauC)
TauR	TauR - Enterobacteriales	Klebsiella pneumoniae subsp. pneumoniae MGH 7	KPN_00571	COG834 (HisJ), COG765 (HisM), COG4215 (ArtQ), COG1126 (GlnQ)
TauR	TauR - Oceanospirillales/Alteromonadales	Chromohalobacter salexigens DSM 3043	Csal_0157	COG161 (Tpa), COG834 (HisJ), COG4160 (ArtM)*2, COG1126 (GlnQ)
TauR	TauR - Oceanospirillales/Alteromonadales	Oceanospirillum sp. MED92	MED92_13211	COG4521 (TauA), COG4525/1116 (TauB), COG600 (TauC)
TauR	TauR - Psychromonadaceae/Aeromonadales	Psychromonas ingrahamii 37	Ping_0046	COG4521 (TauA), COG4525/1116 (TauB), COG600 (TauC), COG161 (Tpa)
TauR	TauR - Nocardiaceae	Rhodococcus sp. RHA1	RHA1_ro03548	COG161 (Tpa), COG280 (Pta), COG1113 (GabP)*2, COG686 (Ald), COG2022 (ThiG), COG214 (PdxS), COG730 (TauE), TF
TauR	TauR - Nocardiaceae	Rhodococcus opacus B4	ROP_33420	COG161 (Tpa), COG280 (Pta), COG1113 (GabP)*2, COG686 (Ald), COG2022 (ThiG), COG730 (TauE), TF
TauR2	TauR2 - Rhodobacterales	Silicibacter pomeroyi DSS-3	SPOA0375	COG3090 (DctM), COG1593 (DctQ), COG1638 (DctP), COG1012 (PutA), COG596 (MhpC)
TauR2	TauR2 - Rhodobacterales	Roseovarius sp. 217	ROS217_02035	COG3090 (DctM), COG1593 (DctQ), COG1638 (DctP), COG1012 (PutA), COG596 (MhpC)
TauR2	TauR2 - Rhizobiales	Rhodopseudomonas palustris CGA009	RPA0105	COG747 (OppA), COG601 (OppB), COG1173 (OppC), COG1123/COG444 (OppD/OppF)*2, COG1574, COG523, COG1012 (PutA), COG730 (TauE), TF
PdxW (Alteromonadales)	PdxW - Alteromonadales	Colwellia psychrerythraea 34H	CPS_3935	COG1670 (RimL), COG2808 (PdxO2)
PdxW (Alteromonadales)	PdxW - Alteromonadales	Pseudoalteromonas tunicata D2	PTD2_06334	COG1670 (RimL), COG2808 (PdxO2)
PdxW (Alteromonadales)	PdxW - Shewanellaceae	Shewanella woodyi ATCC 51908	Swoo_3373	COG1670 (RimL), COG2808 (PdxO2)
Cpin_2755	Cpin_2755 - Sphingobacteria	Chitinophaga pinensis DSM 2588	Cpin_2755	
Cpin_2755	Cpin_2755 - Sphingobacteria	Chitinophaga pinensis DSM 2588	Cpin_3547	COG2128, COG1182 (AcpD), PF00583
Cpin_2755	Cpin_2755 - Sphingobacteria	Pedobacter sp. BAL39	PBAL39_16224	COG702
Cpin_4623	Fjoh_4822 - Flavobacteria	Flavobacterium johnsoniae UW101	Fjoh_4822	COG4206/COG4771 (omp)
Cpin_4623	Fjoh_4822 - Flavobacteria	Flavobacteria bacterium BAL38	FBBAL38_06295	COG4206/COG4771 (omp)
Cpin_4623	Fjoh_4822 - Flavobacteria	Flavobacteriales bacterium HTCC2170	FB2170_07434	COG4206/COG4771 (omp)
Cpin_4623	Fjoh_4822 - Flavobacteria	Polaribacter irgensii 23-P	PI23P_03227	COG4206/COG4771 (omp)
Cpin_4623	Fjoh_4822 - Flavobacteria	Tenacibaculum sp. MED152	MED152_06575	COG4206/COG4771 (omp)
Cpin_4623	Cpin_4623 - Sphingobacteria	Chitinophaga pinensis DSM 2588	Cpin_4623	COG502 (BioB)
Cpin_4623	Cpin_4623 - Sphingobacteria	Dyadobacter fermentans DSM 18053	Dfer_3314	COG4206/COG4771 (omp)
Cpin_4623	Cpin_4623 - Sphingobacteria	Pedobacter sp. BAL39	PBAL39_24288	COG4206/COG4771 (omp)
PdxW (Sphingobacteria)	PdxW - Sphingobacteria	Dyadobacter fermentans DSM 18053	Dfer_1191	COG3467 (PdxO)
PdxW (Sphingobacteria)	PdxW - Sphingobacteria	Chitinophaga pinensis DSM 2588	Cpin_2637	COG456 (RimI), COG1670 (RimL)
PP2642	BBta_7471 - Rhizobiales	Bradyrhizobium sp. BTAi1	BBta_7471	COG2871 (NqrF)
PP2642	Bxe_A4302 - Burkholderia	Burkholderia xenovorans LB400	Bxe_A4302	COG235 (AraD), COG329 (DapA)
PP2642	AB57_0242 - Moraxellaceae	Acinetobacter baumannii AB0057	AB57_0242	COG235 (AraD), COG329 (DapA)
PP2642	Mmwyl1_4437 - Oceanospirillales/Alteromonad	Marinomonas sp. MWYL1	Mmwyl1_4437	COG235 (AraD), COG329 (DapA)
PP2642	PP2642 - Pseudomonadaceae	Pseudomonas putida KT2440	PP2642	COG456 (RimI), COG2871 (NqrF)
PdxR2 (Alphaproteobacteria)	PdxR2 - Rhodobacterales	Paracoccus denitrificans PD1222	Pden_1434	COG2240 (PdxK), COG2258, COG518 (GuaA)
PdxR2 (Alphaproteobacteria)	PdxR2 - Rhodobacterales	Rhodobacter sphaeroides 2.4.1	RSP_1922	COG2240 (PdxK), COG2258
PdxR2 (Alphaproteobacteria)	PdxR2 - Rhizobiales	Nitrobacter winogradskyi Nb-255	Nwi_0881	COG235 (AraD), COG329 (DapA)
PdxR2 (Alphaproteobacteria)	PdxR2 - Sphingomonadales	Zymomonas mobilis subsp. mobilis ZM4	ZMO1854	COG518 (GuaA)
PdxQ (Actinobacteria)	PdxQ - Frankineae/Propionibacterineae/Pseudo	Saccharopolyspora erythraea NRRL 2338	SACE_2681	COG697 (RhaT), TF

PdxQ (Actinobacteria)	PdxQ - Micrococcineae	Arthrobacter aurescens TC1	AAur_0279	COG697 (RhaT), COG2808 (PdxO2), TF
PdxQ (Actinobacteria)	PdxQ - Micrococcineae	Arthrobacter chlorophenolicus A6	Achl_1139	COG697 (RhaT), COG2808 (PdxO2), TF
PdxQ (Actinobacteria)	PdxQ - Micrococcineae	Arthrobacter sp. FB24	Arth_2457	COG697 (RhaT), COG2808 (PdxO2), TF
PdxQ (Actinobacteria)	PdxQ - Micrococcineae	Janibacter sp. HTCC2649	JNB_02495	COG697 (RhaT), TF
PdxQ (Actinobacteria)	PdxQ - Mycobacteriaceae	Mycobacterium abscessus ATCC 19977	MAB_0810c	COG697 (RhaT), TF
PdxQ (Actinobacteria)	PdxQ - Mycobacteriaceae	Mycobacterium smegmatis str. MC2 155	MSMEG_0426	COG697 (RhaT), TF
PdxQ (Actinobacteria)	PdxQ - Nocardiaceae	Rhodococcus erythropolis PR4	RER_10490	COG697 (RhaT), COG436, TF
PdxQ (Actinobacteria)	PdxQ - Streptomycetaceae	Streptomyces avermitilis MA-4680	SAV_6752	COG697 (RhaT), TF
PdxQ (Actinobacteria)	PdxQ - Streptomycetaceae	Streptomyces coelicolor A3(2)	SCO1587	COG697 (RhaT), TF
PdxQ (Actinobacteria)	PdxQ - Streptomycetaceae	Streptomyces griseus subsp. griseus NBRC 13350	SGR_5946	COG697 (RhaT), TF
PdxQ (Actinobacteria)	PdxQ - Streptomycetaceae	Streptomyces scabiei 87.22	SCAB_74111	COG697 (RhaT), TF
PBPRB0322	PTD2_03046 - Alteromonadales	Pseudoalteromonas tunicata D2	PTD2_03046	COG1280 (RhtB), TF
PBPRB0322	Csal_1101 - Oceanospirillales/Alteromonadales	Chromohalobacter salexigens DSM 3043	Csal_1101	COG1280 (RhtB), TF
PBPRB0322	SO2282 - Shewanellaceae	Shewanella amazonensis SB2B	Sama_2016	COG2814 (AraJ), COG1280 (RhtB), TF
PBPRB0322	SO2282 - Shewanellaceae	Shewanella baltica OS155	Sbal_2383	COG2814 (AraJ), COG1280 (RhtB), TF
PBPRB0322	SO2282 - Shewanellaceae	Shewanella halifaxensis HAW-EB4	Shal_1726	COG1280 (RhtB), TF
PBPRB0322	SO2282 - Shewanellaceae	Shewanella oneidensis MR-1	SO2282	COG2814 (AraJ), TF
PBPRB0322	SO2282 - Shewanellaceae	Shewanella pealeana ATCC 700345	Spea_2527	COG1280 (RhtB), TF
PBPRB0322	SO2282 - Shewanellaceae	Shewanella piezotolerans WP3	swp_1877	COG1280 (RhtB), TF
PBPRB0322	SO2282 - Shewanellaceae	Shewanella putrefaciens CN-32	Sputcn32_2134	COG1280 (RhtB), TF
PBPRB0322	SO2282 - Shewanellaceae	Shewanella sp ANA-3	Shewana3_2266	COG2814 (AraJ), COG1280 (RhtB), TF
PBPRB0322	SO2282 - Shewanellaceae	Shewanella sp MR-4	Shewmr4_1753	COG2814 (AraJ), COG1280 (RhtB), TF
PBPRB0322	SO2282 - Shewanellaceae	Shewanella sp MR-7	Shewmr7_1833	COG2814 (AraJ), COG1280 (RhtB), TF
PBPRB0322	SO2282 - Shewanellaceae	Shewanella sp W3-18-1	Sputw3181_1877	COG1280 (RhtB), TF
PBPRB0322	SO2282 - Shewanellaceae	Shewanella woodyi ATCC 51908	Swoo_1966	COG1280 (RhtB), TF
PBPRB0322	PBPRB0322 - Vibrionales	Photobacterium profundum SS9	PBPRB0322	COG1280 (RhtB), TF
PBPRB0322	PBPRB0322 - Vibrionales	Vibrio shilonii AK1	VSAK1_02859	COG1280 (RhtB), TF
PBPRA0298	Tola_2750 - Psychromonadaceae/Aeromonadale	Aeromonas hydrophila subsp. hydrophila ATCC 79	AHA_3592	COG1280 (RhtB), TF
PBPRA0298	Tola_2750 - Psychromonadaceae/Aeromonadale	Tolumonas auensis DSM 9187	Tola_2750	COG1280 (RhtB), TF
PBPRA0298	VSAK1_09283 - Vibrionales	Photobacterium profundum SS9	PBPRA0298	COG1280 (RhtB), TF
PBPRA0298	VSAK1_09283 - Vibrionales	Vibrio angustum S14	VAS14_21252	COG1280 (RhtB), TF
PBPRA0298	VSAK1_09283 - Vibrionales	Vibrio shilonii AK1	VSAK1_09283	COG1280 (RhtB), TF
YrdX2	YrdX2 - Rhizobiales	Rhizobium sp. NGR234	NGR_c19920	COG5349
YrdX2	YrdX2 - Rhizobiales	Sinorhizobium meliloti 1021	SMc01406	COG5349
YrdX2	YrdX2 - Rhizobiales	Xanthobacter autotrophicus Py2	Xaut_0414	COG5349, TF, COG1171 (IlvA), COG251 (TdcF), hypothetical protein
YrdX	YrdX - Rhizobiales	Azorhizobium caulinodans ORS 571	AZC_0074	PF02635, COG388, COG2516, COG3153, COG2144, COG2072 (TrkA), COG438 (RfaG), hypothetical protein
YrdX	YrdX - Rhizobiales	Rhizobium etli CFN 42	RHE_CH01275	COG5349
YrdX	YrdX - Rhizobiales	Rhizobium leguminosarum bv. viciae 3841	RL1422	COG5349, PF02635, COG388, COG2516, COG3153, COG2144, COG2072 (TrkA), COG438 (RfaG), hypothetical protein
YrdX	YrdX - Burkholderia	Burkholderia xenovorans LB400	Bxe_A1406	PF02635, COG388, COG2516, COG3153, COG2144, COG2072 (TrkA), COG438 (RfaG), hypothetical protein
YrdX	YrdX - Burkholderia	Burkholderia vietnamiensis G4	Bcep1808_6050	PF02635, COG388, COG2516, COG3153, COG2144, COG2072 (TrkA), COG438 (RfaG), hypothetical protein
YrdX	YrdX - Burkholderia	Burkholderia phymatum STM815	Bphy_4559	PF02635, COG388, COG2516, COG3153, COG2144, COG2072 (TrkA), COG438 (RfaG), hypothetical protein
YrdX	YrdX - Comamonadaceae	Methylibium petroleiphilum PM1	Mpe_A3480	PF02635, COG388, COG2516, COG3153, COG2144, COG2072 (TrkA), COG438 (RfaG), hypothetical protein
YrdX	YrdX - Comamonadaceae	Polaromonas naphthalenivorans CJ2	Pnap_2106	PF02635, COG388, COG2516, COG3153, COG2144, COG2072 (TrkA), COG438 (RfaG), hypothetical protein
YrdX	YrdX - Comamonadaceae	Variovorax paradoxus S110	Vapar_0563	PF02635, COG388, COG2516, COG3153, COG2144, COG2072 (TrkA), hypothetical protein
YrdX	YrdX - Ralstonia	Ralstonia eutropha H16	H16_A1958	PF02635, COG388, COG2516, COG3153, COG2144, COG2072 (TrkA), hypothetical protein
YrdX3	YrdX3 - Caulobacterales	Caulobacter segnis ATCC 21756	Cseg_0841	COG5349
YrdX3	YrdX3 - Caulobacterales	Caulobacter sp. K31	Caul_2483	COG5349
YrdX3	YrdX3 - Caulobacterales	Caulobacter crescentus CB15	CC3116	COG5349
YrdX3	YrdX3 - Rhodospirillales	Acetobacter pasteurianus IFO 3283-01	APA01_41680	PF02635, COG388, COG2516, COG3153, COG2144, COG2072 (TrkA), COG438 (RfaG)
YrdX3	YrdX3 - Rhodospirillales	Gluconacetobacter diazotrophicus PAl 5	Gdia_2617	PF02635, COG388, COG2516, COG3153, COG2144, COG2072 (TrkA), COG438 (RfaG), hypothetical protein
YrdX3	YrdX3 - Sphingomonadales	Sphingomonas wittichii RW1	Swit_2764	COG5349, TF
YrdX3	YrdX3 - Ralstonia	Ralstonia eutropha H16	H16_A1644	COG1622 (CyoA), COG843 (CyoB), COG1845 (CyoC), COG3125 (CyoD), COG155 (CysI), COG3749
YrdX3	YrdX3 - Ralstonia	Ralstonia eutropha JMP134	Reut_B4580	COG1622 (CyoA), COG843 (CyoB), COG1845 (CyoC), COG3125 (CyoD), TF
YrdX3	YrdX3 - Ralstonia	Ralstonia metallidurans CH34	Rmet_5794	COG1622 (CyoA), COG843 (CyoB), COG1845 (CyoC), COG3125 (CyoD), TF
YrdX3	YrdX3 - Ralstonia	Ralstonia solanacearum GMI1000	RSc1857 (RS03409)	COG1622 (CyoA), COG843 (CyoB), COG1845 (CyoC), COG3125 (CyoD), COG155 (CysI), COG3749
YrdX3	YrdX3(2) - Ralstonia	Ralstonia metallidurans CH34	Rmet_4954	COG1271 (CydA), COG1294 (CydB/AppB)
YrdX3	YrdX3 - Xanthomonadales	Stenotrophomonas maltophilia K279a	Smlt4402	COG1622 (CyoA), COG843 (CyoB), COG1845 (CyoC), COG3125 (CyoD), COG1999
YrdX3	YrdX3 - Xanthomonadales	Stenotrophomonas maltophilia K279a	Smlt4405	
PdxQ2 (Proteobacteria)	PdxQ2 - Caulobacterales	Caulobacter segnis ATCC 21756	Cseg_3419	COG1280 (RhtB), COG384 (PhzF), TF
PdxQ2 (Proteobacteria)	PdxQ2 - Caulobacterales	Caulobacter sp. K31	Caul_0975	COG1280 (RhtB), COG384 (PhzF), TF
PdxQ2 (Proteobacteria)	PdxQ2 - Rhodospirillales	Rhodospirillum rubrum ATCC 11170	Rru_A3041	COG520 (csdA), TF
PdxQ2 (Proteobacteria)	PdxQ2 - Rhodospirillales	Rhodospirillum rubrum ATCC 11170	Rru_A3760	COG1280 (RhtB), TF
PdxQ2 (Proteobacteria)	PdxQ2 - Rhodospirillales	Gluconacetobacter diazotrophicus PAl 5	Gdia_0682	COG384 (PhzF), TF, hypothetical protein
PdxQ2 (Proteobacteria)	PdxQ2 - Rhodospirillales	Gluconacetobacter diazotrophicus PAl 5	Gdia_1400	COG1359
PdxQ2 (Proteobacteria)	PdxQ2 - Rhizobiales	Agrobacterium tumefaciens str. C58 (Cereon)	Atu4266	COG1280 (RhtB), COG384 (PhzF), COG346 (GloA), TF
PdxQ2 (Proteobacteria)	PdxQ2 - Sphingomonadales	Sphingomonas wittichii RW1	Swit_4755	COG259 (PdxH), COG1280 (RhtB), COG384 (PhzF), COG53 (MMT1), TF
PdxQ2 (Proteobacteria)	PdxQ2 - Sphingomonadales	Zymomonas mobilis subsp. mobilis ZM4	ZMO1944	COG384 (PhzF), TF
PdxQ2 (Proteobacteria)	PdxQ2 - Comamonadaceae	Delftia acidovorans SPH-1	Daci_5878	COG384 (PhzF), COG346 (GloA), TF
EF0117	CBY_0654 - Clostridia-1	Clostridium botulinum A str. ATCC 3502	CBO2034	COG384 (PhzF), COG251 (TdcF), TF
EF0117	CBY_0654 - Clostridia-1	Clostridium butyricum 5521	CBY_0654	COG1167 (ARO8), TF
EF0117	CD2285 - Clostridia-2	Clostridium difficile 630	CD2253	COG1794 (RacX), TF
EF0117	CD2285 - Clostridia-2	Clostridium difficile 630	CD2285	COG1296 (AzlC), COG4392 (AzlD), TF
EF0117	CLOBOL_00921 - Clostridia-3	Clostridium bolteae ATCC BAA-613	CLOBOL_00921	COG1167 (ARO8), TF
EF0117	EF0117 - Enterococcaceae	Enterococcus faecalis V583	EF0117	COG2423, COG384 (PhzF), TF
EF0117	EF0117 - Enterococcaceae	Enterococcus faecium DO	EfaeDRAFT_1924	COG2423, COG384 (PhzF)*2, TF
EF0117	LAR_1807 - Lactobacillaceae	Lactobacillus reuteri JCM 1112	LAR_1807	COG1296 (AzlC), TF
EF0117	LAR_1807 - Lactobacillaceae	Leuconostoc mesenteroides subsp. mesenteroides	LEUM_1893	hypothetical membrane protein, TF
EF0117	SMU640c - Streptococcaceae	Streptococcus mutans UA159	SMU.640c	COG604 (Qor), TF
YdeF	YdeF - Bacillales	Bacillus subtilis subsp. subtilis str. 168	BSU05180	COG2271 (UhpC)
YdeF	YdeF - Bacillales	Paenibacillus sp. JDR-2	Pjdr2_3196	COG1231
mll0059	Mll0059 - Rhizobiales	Agrobacterium tumefaciens str. C58 (Cereon)	Atu1848	COG5457, TF
mll0059	Mll0059 - Rhizobiales	Mesorhizobium loti MAFF303099	mll0059	COG5457, TF
mll0059	Mll0059 - Rhizobiales	Rhizobium etli CFN 42	RHE_CH02284	COG384 (PhzF), COG5457, TF
mll0059	Mll0059 - Rhizobiales	Rhizobium leguminosarum bv. viciae 3841	RL2610	COG384 (PhzF), TF
mll0059	Mll0059 - Rhizobiales	Rhizobium sp. NGR234	NGR_c16240	COG384 (PhzF), TF
mll0059	Mll0059 - Rhizobiales	Sinorhizobium meliloti 1021	SMc00490	COG384 (PhzF), COG5457, TF
PdxQ3 (Proteobacteria)	PdxQ3 - Rhodobacterales	Silicibacter pomeroyi DSS-3	SPOA0149	COG2814 (AraJ)
PdxQ3 (Proteobacteria)	PdxQ3 - Rhodobacterales	Roseovarius nubinhibens ISM	ISM_17235	COG2814 (AraJ)
PdxQ3 (Proteobacteria)	PdxQ3 - Rhizobiales	Azorhizobium caulinodans ORS 571	AZC_4467	COG5457
PdxQ3 (Proteobacteria)	PdxQ3 - Rhizobiales	Bradyrhizobium sp. BTAi1	BBta_6780	COG384 (PhzF), COG5457
PdxQ3 (Proteobacteria)	PdxQ3 - Rhizobiales	Bradyrhizobium japonicum USDA 110	blr5860	COG5457
PdxQ3 (Proteobacteria)	PdxQ3 - Burkholderia	Burkholderia phymatum STM815	Bphy_5571	COG4977
PdxQ3 (Proteobacteria)	PdxQ3 - Burkholderia	Burkholderia pseudomallei K96243	BPSS0449	COG4977
PdxQ3 (Proteobacteria)	PdxQ3 - Burkholderia	Burkholderia sp. 383	Bcep18194_C7710	COG730
PdxQ3 (Proteobacteria)	PdxQ3 - Comamonadaceae	Delftia acidovorans SPH-1	Daci_2843	COG2050 (PaaI)
PdxQ3 (Proteobacteria)	PdxQ3 - Comamonadaceae	Methylibium petroleiphilum PM1	Mpe_A2041	COG4977, COG2808 (PdxO2)
PdxQ3 (Proteobacteria)	PdxQ3 - Ralstonia	Ralstonia eutropha H16	H16_B1957	COG1280 (RhtB), PF03992
PdxQ3 (Proteobacteria)	PdxQ3 - Oceanospirillales/Alteromonadales	Chromohalobacter salexigens DSM 3043	Csal_1090	COG2814 (AraJ)
PdxQ3 (Proteobacteria)	PdxQ3 - Pseudomonadaceae	Pseudomonas fluorescens Pf-5	PFL_2406	COG697 (RhaT)
PdxQ3 (Proteobacteria)	PdxQ3 - Pseudomonadaceae	Pseudomonas fluorescens Pf-5	PFL_4112	COG1280 (RhtB)
PdxQ3 (Proteobacteria)	PdxQ3 - Pseudomonadaceae	Pseudomonas mendocina ymp	Pmen_1514	COG4977
PdxQ3 (Proteobacteria)	PdxQ3 - Mycobacteriaceae	Mycobacterium abscessus ATCC 19977	MAB_3582	COG2050 (PaaI)
PdxQ3 (Proteobacteria)	PdxQ3 - Nocardiaceae	Nocardia farcinica IFM 10152	nfa20260	COG2050 (PaaI)
PdxQ3 (Proteobacteria)	PdxQ3 - Streptomycetaceae	Streptomyces avermitilis MA-4680	SAV_4411	COG596 (MhpC)
PdxQ3 (Proteobacteria)	PdxQ3 - Streptomycetaceae	Streptomyces avermitilis MA-4680	SAV_4412	
PdxQ3 (Proteobacteria)	PdxQ3 - Streptomycetaceae	Streptomyces griseus subsp. griseus NBRC 13350	SGR_1612	COG1280 (RhtB)
PdxQ3 (Proteobacteria)	PdxQ3 - Streptomycetaceae	Streptomyces scabiei 87.22	SCAB_44761	COG596 (MhpC)
EutR	EutR - Rhodobacterales	Loktanella vestfoldensis SKA53	SKA53_04438	TF, COG5457
EutR	EutR - Rhodobacterales	Oceanicola batsensis HTCC2597	OB2597_13923	COG3090 (TeaB), COG1593 (TeaC), COG1638 (TeaA), COG589 (UspA)
EutR	EutR - Rhodobacterales	Paracoccus denitrificans PD1222	Pden_0279	COG1126 (EhuA), COG834 (EhuB), COG765 (EhuC), COG765 (EhuD), COG3473 (EutA), COG1171 (EutB), COG2423 (EutC), COG6 (EutD), COG3608 (EutE), COG1012 (DoeC), COG161(DoeD)
EutR	EutR - Rhodobacterales	Rhodobacter sphaeroides 2.4.1	RSP_0018	TF, COG5457
EutR	EutR - Rhodobacterales	Roseobacter sp. MED193	MED193_04751	TF
EutR	EutR - Rhodobacterales	Roseovarius nubinhibens ISM	ISM_16300	TF, COG5457
EutR	EutR - Rhodobacterales	Silicibacter TM1040	TM1040_1386	TF
EutR	EutR - Rhodobacterales	Silicibacter pomeroyi DSS-3	SPO1690	TF, COG5457

EutR	EutR - Rhodobacterales	Silicibacter pomeroyi DSS-3	SPO1148	COG3090 (TeaB), COG1593 (TeaC), COG1638 (TeaA), COG589 (UspA), COG3473 (EutA), COG1171 (EutB), COG2423 (EutC), COG6 (EutD), COG3608 (EutE), COG1522 (DoeX), COG1012 (DoeC), COG161(DoeD)
EutR	EutR - Rhodobacterales	Sulfitobacter sp. EE-36	EE36_14882	TF, COG5457
EutR	EutR - Rhizobiales	Agrobacterium tumefaciens str. C58 (Cereon)	Atu4760	TF, COG1126 (EhuA), COG834 (EhuB), COG765 (EhuC), COG765 (EhuD), COG1171 (EutB), COG2423 (EutC), COG6 (EutD), COG3608 (EutE)
EutR	EutR - Rhizobiales	Mesorhizobium loti MAFF303099	mlr7131	TF, COG1126 (EhuA), COG834 (EhuB), COG765 (EhuC), COG765 (EhuD), COG3473 (EutA), COG1171 (EutB), COG2423 (EutC), COG6 (EutD), COG3608 (EutE), COG1522 (DoeX), COG1012 (DoeC), COG161(DoeD), COG604 (Qor)
EutR	EutR - Rhizobiales	Rhizobium etli CFN 42	RHE_PF00188	TF, COG1126 (EhuA), COG834 (EhuB), COG765 (EhuC), COG765 (EhuD), COG3473 (EutA), COG1171 (EutB), COG2423 (EutC), COG6 (EutD), COG3608 (EutE), COG1522 (DoeX)
EutR	EutR - Rhizobiales	Rhizobium leguminosarum bv. viciae 3841	pRL120046	TF, COG1126 (EhuA), COG834 (EhuB), COG765 (EhuC), COG765 (EhuD), COG3473 (EutA), COG1171 (EutB), COG2423 (EutC), COG6 (EutD), COG3608 (EutE), COG1522 (DoeX), COG1012 (DoeC), COG161(DoeD), COG604 (Qor)
EutR	EutR - Rhizobiales	Rhizobium sp. NGR234	NGR_b23330	TF, COG1126 (EhuA), COG834 (EhuB), COG765 (EhuC), COG765 (EhuD), COG3473 (EutA), COG1171 (EutB)*2, COG2423 (EutC)*2, COG6 (EutD)*3, COG3608 (EutE), COG1522 (DoeX)
EutR	EutR - Rhizobiales	Sinorhizobium meliloti 1021	SMb20426	TF, COG1126 (EhuA), COG834 (EhuB), COG765 (EhuC), COG765 (EhuD), COG3473 (EutA), COG1171 (EutB), COG2423 (EutC), COG6 (EutD), COG3608 (EutE), COG1522 (DoeX), COG1012 (DoeC), COG161(DoeD), COG604 (Qor)
EutR	EutR - Burkholderia	Burkholderia cepacia AMMD	Bamb_6017	COG1171 (EutB), COG2423 (EutC), COG6 (EutD), COG3608 (EutE), COG1522 (DoeX), COG1012 (DoeC), COG161(DoeD), TI
EutR	EutR - Burkholderia	Burkholderia phymatum STM815	Bphy_3865	COG1126 (EhuA), COG834 (EhuB), COG765 (EhuC), COG765 (EhuD), COG1171 (EutB), COG2423 (EutC), COG6 (EutD), COG3608 (EutE), COG1522 (DoeX), COG1012 (DoeC), COG161(DoeD), TF
EutR	EutR - Burkholderia	Burkholderia vietnamiensis G4	Bcep1808_5465	COG1171 (EutB), COG2423 (EutC), COG6 (EutD), COG3608 (EutE), COG1522 (DoeX), COG1012 (DoeC), COG161(DoeD), TF
EutR	EutR - Burkholderia	Burkholderia xenovorans LB400	Bxe_C0055	COG1126 (EhuA), COG834 (EhuB), COG765 (EhuC), COG765 (EhuD), COG3473 (EutA), COG1171 (EutB), COG2423 (EutC), COG6 (EutD), COG3608 (EutE), COG1522 (DoeX), COG1012 (DoeC), COG161(DoeD), TF
EutR	EutR - Comamonadaceae	Verminephrobacter eiseniae EF01-2	Veis_2152	COG1126 (EhuA), COG834 (EhuB), COG765 (EhuC), COG765 (EhuD), COG1171 (EutB), COG2423 (EutC), COG6 (EutD), COG3608 (EutE), COG1522 (DoeX), TF
EutR	EutR - Oceanospirillales/Alteromonadales	Chromohalobacter salexigens DSM 3043	Csal_2733	COG6 (EutD), COG3608 (EutE), COG1522 (DoeX), TF
EutR	EutR - Pseudomonadaceae	Pseudomonas putida KT2440	PP4429	COG1126 (EhuA), COG834 (EhuB), COG765 (EhuC), COG765 (EhuD), COG1171 (EutB), COG2423 (EutC), COG6 (EutD), TF
Tola_2572	PP0486 - Pseudomonadaceae	Pseudomonas entomophila L48	PSEEN3858	COG3135 (BenE), COG665 (DadA), COG251 (TdcF), COG498 (ThrC), COG112 (GlyA), TF
Tola_2572	PP0486 - Pseudomonadaceae	Pseudomonas fluorescens Pf-5	PFL_4913	COG3135 (BenE), TF
Tola_2572	PP0486 - Pseudomonadaceae	Pseudomonas putida KT2440	PP0486	COG4221, COG730, TF
Tola_2572	Tola_2572 - Psychromonadaceae/Aeromonadale	Tolumonas auensis DSM 9187	Tola_2572	COG2084 (MmsB), TF
Tola_2572	Tola_2572 - Psychromonadaceae/Aeromonadale	Moritella sp. PE36	PE36_04298	COG2084 (MmsB), COG730, TF
Tola_2572	Tola_2572 - Psychromonadaceae/Aeromonadale	Psychromonas sp. CNPT3	PCNPT3_11514	COG2084 (MmsB), TF
Tola_2572	VSAL_I1306 - Vibrionales	Vibrio angustum S14	VAS14_03178	COG2084 (MmsB), TF
Tola_2572	VSAL_I1306 - Vibrionales	Vibrio salmonicida LFI1238	VSAL_I1306	COG2084 (MmsB), COG730, hypothetical protein, TF
PA2100	TERTU_3902 - Oceanospirillales/Alteromonadale	Teredinibacter turnerae T7901	TERTU_3902	COG697 (RhaT)
PA2100	PA2100 - Pseudomonadaceae	Pseudomonas aeruginosa PAO1	PA2100	COG697 (RhaT), COG1310
PA2100	PA2100 - Pseudomonadaceae	Pseudomonas fluorescens Pf-5	PFL_1867	COG697 (RhaT)
PdxR3 (Actinobacteria)	PdxR3 - Caulobacterales	Phenylobacterium zucineum HLK1	PHZ_c3113	COG2364
PdxR3 (Actinobacteria)	PdxR3 - Rhodobacterales	Oceanicaulis alexandrii HTCC2633	OA2633_06364	COG2364
PdxR3 (Actinobacteria)	PdxR3 - Comamonadaceae	Methylibium petroleiphilum PM1	Mpe_A2441	COG596 (MhpC), COG657 (Aes), TF
PdxR3 (Actinobacteria)	PdxR3 - Enterobacteriales	Citrobacter koseri ATCC BAA-895	CKO_02700	COG2364
PdxR3 (Actinobacteria)	PdxR3 - Enterobacteriales	Enterobacter sp. 638	Ent638_0925	COG2364
PdxR3 (Actinobacteria)	PdxR3 - Enterobacteriales	Klebsiella pneumoniae subsp. pneumoniae MGH 7	KPN_00421	COG2364
PdxR3 (Actinobacteria)	PdxR3 -Frankineae/Propionibacterineae/Pseudo	Actinosynnema mirum DSM 43827	Amir_6790	COG2364
PdxR3 (Actinobacteria)	PdxR3 -Frankineae/Propionibacterineae/Pseudo	Nakamurella multipartita DSM 44233	Namu_3402	COG2364, PF10027, COG311 (PdxT), COG217
PdxR3 (Actinobacteria)	PdxR3 -Frankineae/Propionibacterineae/Pseudo	Nakamurella multipartita DSM 44233	Namu_1043	
PdxR3 (Actinobacteria)	PdxR3 -Frankineae/Propionibacterineae/Pseudo	Nocardioides sp. JS614	Noca_3373	COG2364
PdxR3 (Actinobacteria)	PdxR3 -Frankineae/Propionibacterineae/Pseudo	Saccharopolyspora erythraea NRRL 2338	SACE_7001	COG2364
PdxR3 (Actinobacteria)	PdxR3 - Micrococcineae	Arthrobacter aurescens TC1	AAur_0773	COG2364
PdxR3 (Actinobacteria)	PdxR3 - Micrococcineae	Arthrobacter sp. FB24	Arth_0620	COG2364
PdxR3 (Actinobacteria)	PdxR3 - Micrococcineae	Arthrobacter chlorophenolicus A6	Achl_0748	COG2364
PdxR3 (Actinobacteria)	PdxR3 - Micrococcineae	Beutenbergia cavernae DSM 12333	Bcav_3924	COG2364
PdxR3 (Actinobacteria)	PdxR3 - Micrococcineae	Brevibacterium linens BL2	BlinB01002592	COG2364
PdxR3 (Actinobacteria)	PdxR3 - Micrococcineae	Clavibacter michiganensis subsp. michiganensis NC	CMM_0763	COG2364
PdxR3 (Actinobacteria)	PdxR3 - Micrococcineae	Janibacter sp. HTCC2649	JNB_06014	COG2364
PdxR3 (Actinobacteria)	PdxR3 - Mycobacteriaceae	Mycobacterium abscessus ATCC 19977	MAB_3387	COG2364
PdxR3 (Actinobacteria)	PdxR3 - Mycobacteriaceae	Mycobacterium gilvum PYR-GCK	Mflv_4275	COG2364
PdxR3 (Actinobacteria)	PdxR3 - Mycobacteriaceae	Mycobacterium smegmatis str. MC2 155	MSMEG_1043	COG2364
PdxR3 (Actinobacteria)	PdxR3 - Mycobacteriaceae	Mycobacterium smegmatis str. MC2 155	MSMEG_2323	COG2364
PdxR3 (Actinobacteria)	PdxR3 - Mycobacteriaceae	Mycobacterium sp. JLS	Mjls_1835	COG2364
PdxR3 (Actinobacteria)	PdxR3 - Mycobacteriaceae	Mycobacterium vanbaalenii PYR-1	Mvan_2084	COG2364
PdxR3 (Actinobacteria)	PdxR3 - Nocardiaceae	Nocardia farcinica IFM 10152	nfa53660	COG350 (Ada), TF
PdxR3 (Actinobacteria)	PdxR3 - Nocardiaceae	Rhodococcus sp. RHA1	RHA1_ro05556	TF
PdxR3 (Actinobacteria)	PdxR3 - Nocardiaceae	Rhodococcus sp. RHA1	RHA1_ro06371	COG2364, TF
PdxR3 (Actinobacteria)	PdxR3 - Nocardiaceae	Rhodococcus opacus B4	ROP_64330	COG2364, TF
PdxR3 (Actinobacteria)	PdxR3 - Nocardiaceae	Rhodococcus opacus B4	ROP_56250	COG350 (Ada), TF
PdxR3 (Actinobacteria)	PdxR3 - Nocardiaceae	Rhodococcus erythropolis PR4	RER_14310	COG2364, TF
PdxR3 (Actinobacteria)	PdxR3 - Streptomycetaceae	Streptomyces avermitilis MA-4680	SAV_6928	COG2364, COG584 (UgpQ), COG2270, TF
PdxR3 (Actinobacteria)	PdxR3 - Streptomycetaceae	Streptomyces avermitilis MA-4680	SAV_980	COG2364, TF
PdxR3 (Actinobacteria)	PdxR3 - Streptomycetaceae	Streptomyces coelicolor A3(2)	SCO1417	COG2364, COG584 (UgpQ), COG2270, TF
PdxR3 (Actinobacteria)	PdxR3 - Streptomycetaceae	Streptomyces griseus subsp. griseus NBRC 13350	SGR_6115	COG2364, COG584 (UgpQ), COG2270, TF
PdxR3 (Actinobacteria)	PdxR3 - Streptomycetaceae	Streptomyces griseus subsp. griseus NBRC 13350	SGR_4328	COG2364, TF
PdxR3 (Actinobacteria)	PdxR3 - Streptomycetaceae	Streptomyces scabiei 87.22	SCAB_75881	COG2364, COG584 (UgpQ), COG2270, TF
Daci_0288	SPOA0164 - Rhodobacterales	Silicibacter pomeroyi DSS-3	SPOA0164	COG1257 (MvaA), COG4663 (FcbT1), COG4665/COG3090 (FcbT2), COG4664 (FcbT3), TF
Daci_0288	SPOA0164 - Rhodobacterales	Oceanicola batsensis HTCC2597	OB2597_01522	COG1257 (MvaA), COG4663 (FcbT1), COG4665/COG3090 (FcbT2), COG4664 (FcbT3), TF
Daci_0288	Bpet3338 - Alcaligenaceae	Bordetella petrii DSM 12804	Bpet3338	COG1257 (MvaA), COG4663 (FcbT1), COG4665/COG3090 (FcbT2), COG4664 (FcbT3), TF
Daci_0288	Daci_0288 - Comamonadaceae	Delftia acidovorans SPH-1	Daci_0288	COG1257 (MvaA), hypothetical protein, hypothetical protein
GabR2 (Proteobacteria)	GabR2 - Comamonadaceae	Polaromonas naphthalenivorans CJ2	Pnap_2629	COG3842 (GbtA), COG1176 (GbtB), COG1177 (GbtC), COG687 (GbtD), COG160 (GabT), COG1012 (GabD), COG10 (SpeB)
GabR2 (Proteobacteria)	GabR2 - Comamonadaceae	Polaromonas sp. JS666	Bpro_2911	COG3842 (GbtA), COG1176 (GbtB), COG1177 (GbtC), COG687 (GbtD), COG160 (GabT), COG1012 (GabD)
GabR2 (Proteobacteria)	GabR2 - Comamonadaceae	Rhodoferax ferrireducens DSM 15236	Rfer_0591	COG3842 (GbtA), COG1176 (GbtB), COG1177 (GbtC), COG687 (GbtD), COG160 (GabT), COG1012 (GabD), COG10 (SpeB)
GabR2 (Proteobacteria)	GabR2 - Comamonadaceae	Variovorax paradoxus S110	Vapar_4694	COG3842 (GbtA), COG1176 (GbtB), COG1177 (GbtC), COG687 (GbtD), COG160 (GabT), COG1012 (GabD), COG10 (SpeB)
GabR2 (Proteobacteria)	GabR2 - Pseudomonadaceae	Pseudomonas entomophila L48	PSEEN2394	COG1113 (GabP)
GabR2 (Proteobacteria)	GabR2 - Pseudomonadaceae	Pseudomonas putida KT2440	PP2542	COG1113 (GabP)
OapR	OapR - Rhodospirillales	Acetobacter pasteurianus IFO 3283-01	APA01_21590	COG161 (OapT), TF
OapR	OapR - Rhizobiales	Azorhizobium caulinodans ORS 571	AZC_0730	COG161 (OapT)
OapR	OapR - Alcaligenaceae	Bordetella avium 197N	BAV0505	COG161 (OapT), COG1012 (OapD)
OapR	OapR - Alcaligenaceae	Bordetella bronchiseptica RB50	BB0870	COG161 (OapT), COG1012 (OapD)
OapR	OapR - Alcaligenaceae	Bordetella petrii DSM 12804	Bpet4036	COG161 (OapT), COG1012 (OapD)
OapR	OapR - Burkholderia	Burkholderia phymatum STM815	Bphy_4469	COG161 (OapT)
OapR	OapR - Burkholderia	Burkholderia xenovorans LB400	Bxe_B0671	COG161 (OapT)
OapR	OapR - Ralstonia	Cupriavidus taiwanensis	RALTA_A0211	COG161 (OapT), COG1012 (OapD)
OapR	OapR - Ralstonia	Ralstonia eutropha H16	H16_A0271	COG161 (OapT), COG1012 (OapD)
OapR	OapR - Ralstonia	Ralstonia eutropha JMP134	Reut_A0247	COG161 (OapT), COG1012 (OapD)
OapR	OapR - Ralstonia	Ralstonia metallidurans CH34	Rmet_0204	COG161 (OapT), COG1012 (OapD)
OapR	OapR - Ralstonia	Ralstonia pickettii 12J	Rpic_4272	COG161 (OapT), COG1012 (OapD)
OapR	OapR - Ralstonia	Ralstonia solanacearum GMI1000	RS02394 (RSp1057)	COG161 (OapT), COG1012 (OapD)
RALTA_A0216	Bcep1808_4966 - Burkholderia	Burkholderia phymatum STM815	Bphy_0548	PF11142
RALTA_A0216	Bcep1808_4966 - Burkholderia	Burkholderia vietnamiensis G4	Bcep1808_4966	COG1280 (RhtB)
RALTA_A0216	RALTA_A0216 - Ralstonia	Cupriavidus taiwanensis	RALTA_A0216	PF11142
RALTA_A0216	RALTA_A0216 - Ralstonia	Ralstonia eutropha H16	H16_A0278	PF11142
RALTA_A0216	RALTA_A0216 - Ralstonia	Ralstonia eutropha JMP134	Reut_A0251	PF11142
RALTA_A0216	RALTA_A0216 - Ralstonia	Ralstonia metallidurans CH34	Rmet_0208	PF11142
RALTA_A0216	RALTA_A0216 - Ralstonia	Ralstonia pickettii 12J	Rpic_4705	PF11142
PdxW2 (Methylophilaceae)	PdxW2 - Various betaproteobacteria	Methylotenera mobilis JLW8	Mmol_1448	COG2808 (PdxO2), hypothetical protein
ACIAD1897	Pden_4836 - Rhodobacterales	Paracoccus denitrificans PD1222	Pden_4836	COG697 (RhaT)
ACIAD1897	SMb20039 - Rhizobiales	Agrobacterium tumefaciens str. C58 (Cereon)	Atu0235	COG697 (RhaT)
ACIAD1897	SMb20039 - Rhizobiales	Azorhizobium caulinodans ORS 571	AZC_0292	COG697 (RhaT)
ACIAD1897	SMb20039 - Rhizobiales	Bradyrhizobium japonicum USDA 110	blr0404	COG697 (RhaT)
ACIAD1897	SMb20039 - Rhizobiales	Bradyrhizobium sp. BTAi1	BBta_7475	COG697 (RhaT)
ACIAD1897	SMb20039 - Rhizobiales	Brucella melitensis 16M	BMEI0169	COG697 (RhaT)
ACIAD1897	SMb20039 - Rhizobiales	Mesorhizobium loti MAFF303099	mlr1139	COG697 (RhaT)
ACIAD1897	SMb20039 - Rhizobiales	Mesorhizobium sp. BNC1	Meso_0253	COG697 (RhaT)
ACIAD1897	SMb20039 - Rhizobiales	Rhizobium etli CFN 42	RHE_CH02666	COG697 (RhaT)
ACIAD1897	SMb20039 - Rhizobiales	Rhizobium leguminosarum bv. viciae 3841	RL3118	COG697 (RhaT)
ACIAD1897	SMb20039 - Rhizobiales	Rhizobium sp. NGR234	NGR_b00120	COG697 (RhaT)
ACIAD1897	SMb20039 - Rhizobiales	Sinorhizobium meliloti 1021	SMb20039	COG697 (RhaT)

ACIAD1897	Bamb_4265 - Burkholderia	Burkholderia cepacia AMMD	Bamb_4265	COG697 (RhaT)
ACIAD1897	Bamb_4265 - Burkholderia	Burkholderia glumae BGR1	bglu_2g04010	COG697 (RhaT)
ACIAD1897	Bamb_4265 - Burkholderia	Burkholderia sp. 383	Bcep18194_B0828	COG697 (RhaT)
ACIAD1897	Bamb_4265 - Burkholderia	Burkholderia vietnamiensis G4	Bcep1808_5370	COG697 (RhaT)
ACIAD1897	Daci_1890 - Comamonadaceae	Comamonas testosteroni KF-1	CtesDRAFT_0887	COG697 (RhaT)
ACIAD1897	Daci_1890 - Comamonadaceae	Delftia acidovorans SPH-1	Daci_1890	COG697 (RhaT)
ACIAD1897	ACIAD1897 - Moraxellaceae	Acinetobacter sp. ADP1	ACIAD1897	COG697 (RhaT)
ACIAD1897	ACIAD1897 - Moraxellaceae	Acinetobacter baumannii AB0057	AB57_2342	COG697 (RhaT)
ACIAD1897	Mmwyl1_0258 - Oceanospirillales/Alteromonad	Marinomonas sp. MWYL1	Mmwyl1_0258	COG697 (RhaT)
ACIAD1897	PP2948 - Pseudomonadaceae	Azotobacter vinelandii AvOP	Avin_48160	COG697 (RhaT)
ACIAD1897	PP2948 - Pseudomonadaceae	Pseudomonas putida KT2440	PP2948	COG697 (RhaT)
ACIAD1897	Smlt1075 - Xanthomonadales	Stenotrophomonas maltophilia K279a	Smlt1075	COG697 (RhaT)
Daci_0595	BAV2922 - Alcaligenaceae	Bordetella avium 197N	BAV2922	COG697 (RhaT), COG1280 (RhtB)
Daci_0595	BAV2922 - Alcaligenaceae	Bordetella bronchiseptica RB50	BB4262	COG697 (RhaT), COG1280 (RhtB)
Daci_0595	BAV2922 - Alcaligenaceae	Bordetella petrii DSM 12804	Bpet0647	COG697 (RhaT), COG1280 (RhtB)
Daci_0595	BPSL1086 - Burkholderia	Burkholderia cepacia AMMD	Bamb_2384	Pfam13468, COG697 (RhaT), COG384 (PhzF), COG1280 (RhtB), COG251 (TdcF), COG1167 (ARO8)
Daci_0595	BPSL1086 - Burkholderia	Burkholderia glumae BGR1	bglu_1g26830	Pfam13468, COG697 (RhaT), COG1280 (RhtB), COG251 (TdcF), COG1167 (ARO8)
Daci_0595	BPSL1086 - Burkholderia	Burkholderia mallei ATCC 23344	BMA1949	Pfam13468, COG697 (RhaT), COG1280 (RhtB), COG1167 (ARO8)
Daci_0595	BPSL1086 - Burkholderia	Burkholderia phymatum STM815	Bphy_2114	COG1280 (RhtB)
Daci_0595	BPSL1086 - Burkholderia	Burkholderia pseudomallei K96243	BPSL1086	Pfam13468, COG697 (RhaT), COG384 (PhzF), COG1280 (RhtB), COG251 (TdcF), COG1167 (ARO8)
Daci_0595	BPSL1086 - Burkholderia	Burkholderia sp. 383	Bcep18194_A5687	Pfam13468, COG697 (RhaT), COG384 (PhzF), COG1280 (RhtB), COG251 (TdcF), COG1167 (ARO8)
Daci_0595	BPSL1086 - Burkholderia	Burkholderia vietnamiensis G4	Bcep1808_2435	Pfam13468, COG697 (RhaT), COG384 (PhzF), COG1280 (RhtB), COG251 (TdcF), COG1167 (ARO8)
Daci_0595	BPSL1086 - Burkholderia	Burkholderia xenovorans LB400	Bxe_A3330	Pfam13468, COG384 (PhzF), COG1280 (RhtB), COG251 (TdcF), COG1167 (ARO8)
Daci_0595	Daci_0595 - Comamonadaceae	Acidovorax avenae subsp. citrulli AAC00-1	Aave_4434	Pfam13468, COG625 (Gst), COG697 (RhaT), COG384 (PhzF), COG1280 (RhtB), COG1167 (ARO8), COG1171 (IlvA)
Daci_0595	Daci_0595 - Comamonadaceae	Acidovorax sp. JS42	Ajs_3842	COG697 (RhaT), COG384 (PhzF), COG1280 (RhtB), COG1167 (ARO8), COG1171 (IlvA)
Daci_0595	Daci_0595 - Comamonadaceae	Comamonas testosteroni KF-1	CtesDRAFT_1191	COG1280 (RhtB)
Daci_0595	Daci_0595 - Comamonadaceae	Comamonas testosteroni KF-1	CtesDRAFT_4336	COG384 (PhzF), COG1167 (ARO8)
Daci_0595	Daci_0595 - Comamonadaceae	Delftia acidovorans SPH-1	Daci_0595	COG384 (PhzF), COG1280 (RhtB), COG1167 (ARO8)
Daci_0595	Daci_0595 - Comamonadaceae	Delftia acidovorans SPH-1	Daci_3668	COG1280 (RhtB)
Daci_0595	Daci_0595 - Comamonadaceae	Methylibium petroleiphilum PM1	Mpe_A0183	COG697 (RhaT), COG384 (PhzF), COG1794 (RacX), COG1167 (ARO8)
Daci_0595	Daci_0595 - Comamonadaceae	Polaromonas naphthalenivorans CJ2	Pnap_0403	COG697 (RhaT), COG384 (PhzF), COG1167 (ARO8)
Daci_0595	Daci_0595 - Comamonadaceae	Polaromonas sp. JS666	Bpro_4233	PF03992, COG4977, COG1171 (IlvA), Pfam13468, COG625 (Gst), COG697 (RhaT), COG384 (PhzF), COG1280 (RhtB)*2, COG1167 (ARO8), hypothetical protein, hypothetical protein
Daci_0595	Daci_0595 - Comamonadaceae	Rhodoferax ferrireducens DSM 15236	Rfer_3725	COG625 (Gst), COG697 (RhaT), COG384 (PhzF), COG1280 (RhtB), COG1794 (RacX), COG1167 (ARO8)
Daci_0595	Daci_0595 - Comamonadaceae	Variovorax paradoxus S110	Vapar_0748	COG4977, COG1171 (IlvA), Pfam13468, COG625 (Gst), COG697 (RhaT), COG384 (PhzF), COG1280 (RhtB)*3, COG1167 (ARO8)
Daci_0595	H16_A2655 - Ralstonia	Cupriavidus taiwanensis	RALTA_A2147	COG697 (RhaT), COG625 (Gst), COG1280 (RhtB)*2, Pfam13468, COG2872, COG251 (TdcF), COG1167 (ARO8)
Daci_0595	H16_A2655 - Ralstonia	Ralstonia eutropha JMP134	Reut_A2343	COG697 (RhaT), COG625 (Gst), COG1280 (RhtB)*2, Pfam13468, COG2872, COG251 (TdcF), COG1167 (ARO8), PF07883
Daci_0595	H16_A2655 - Ralstonia	Ralstonia eutropha H16	H16_A2655	COG697 (RhaT), COG625 (Gst), COG1280 (RhtB)*2, Pfam13468, COG2872, COG251 (TdcF), COG1167 (ARO8), PF07883
Daci_0595	H16_A2655 - Ralstonia	Ralstonia metallidurans CH34	Rmet_2507	COG697 (RhaT), COG625 (Gst), COG1280 (RhtB)*2, Pfam13468, COG2872, COG251 (TdcF), COG1167 (ARO8)
Daci_0595	H16_A2655 - Ralstonia	Ralstonia pickettii 12J	Rpic_0850	COG697 (RhaT), COG625 (Gst), COG2872, COG251 (TdcF), COG1167 (ARO8)
Daci_0595	H16_A2655 - Ralstonia	Ralstonia solanacearum GMI1000	RSc0989 (RS04303)	COG697 (RhaT), COG625 (Gst), COG2872, COG251 (TdcF), COG1167 (ARO8)
DVU0030	DVU0030 - Desulfovibrionales	Desulfovibrio vulgaris Hildenborough	DVU0030	COG1296 (AzlC), COG4392 (AzlD), TF
DVU0030	DVU0030 - Desulfovibrionales	Desulfomicrobium baculatum DSM 4028	Dbac_3052	COG1296 (AzlC), COG4392 (AzlD), COG1167 (ARO8), TF
DVU0030	DVU0030 - Desulfovibrionales	Desulfovibrio salexigens DSM 2638	Desal_3215	COG1296 (AzlC), COG4392 (AzlD), COG2316, TF
DVU0030	DVU0030 - Desulfovibrionales	Desulfovibrio vulgaris str. Miyazaki F	DvMF_2175	COG1296 (AzlC), COG4392 (AzlD), TF
DVU0030	DVU0030 - Desulfovibrionales	Desulfovibrio desulfuricans G20	Dde_0157	COG1296 (AzlC), COG4392 (AzlD), TF
DVU0030	DVU0030 - Desulfovibrionales	Desulfovibrio magneticus RS-1	DMR_35550	TF
DVU0030	DVU0030 - Desulfovibrionales	Desulfohalobium retbaense DSM 5692	Dret_1471	COG1296 (AzlC), COG4392 (AzlD), TF
VEA_000881	Daci_4286 - Comamonadaceae	Delftia acidovorans SPH-1	Daci_4286	COG2162 (NhoA)
VEA_000881	CPS_4612 - Alteromonadales	Pseudoalteromonas tunicata D2	PTD2_06140	COG697 (RhaT)
VEA_000881	CPS_4612 - Alteromonadales	Pseudoalteromonas haloplanktis TAC125	PSHAa0059	COG697 (RhaT)
VEA_000881	CPS_4612 - Alteromonadales	Colwellia psychrerythraea 34H	CPS_4612	COG697 (RhaT)
VEA_000881	MED92_15328 - Oceanospirillales/Alteromonad	Marinomonas sp. MWYL1	Mmwyl1_3154	COG1271 (CydA), COG1294 (CydB/AppB)
VEA_000881	MED92_15328 - Oceanospirillales/Alteromonad	Oceanospirillum sp. MED92	MED92_15328	COG348 (NapH)
VEA_000881	VEA_000881 - Vibrionales	Vibrio splendidus LGP32	VS_II1306	COG697 (RhaT)
VEA_000881	VEA_000881 - Vibrionales	Vibrio parahaemolyticus RIMD 2210633	VPA0118	COG697 (RhaT)
VEA_000881	VEA_000881 - Vibrionales	Vibrio harveyi ATCC BAA-1116	VIBHAR_06858	COG697 (RhaT)
VEA_000881	VEA_000881 - Vibrionales	Vibrio vulnificus CMCP6	VV2_1401	COG697 (RhaT)
VEA_000881	VEA_000881 - Vibrionales	Vibrio angustum S14	VAS14_15734	COG697 (RhaT)
VEA_000881	VEA_000881 - Vibrionales	Photobacterium profundum SS9	PBPRA2308	COG697 (RhaT)
VEA_000881	Cpin_6695 - Sphingobacteria	Chitinophaga pinensis DSM 2588	Cpin_6695	SSF55729
YE4144	CV1900 - Various betaproteobacteria	Chromobacterium violaceum ATCC 12472	CV1900	COG1280 (RhtB)
YE4144	CV1900 - Various betaproteobacteria	Chromobacterium violaceum ATCC 12472	CV1935	COG1280 (RhtB), TF
YE4144	YE4144 - Enterobacteriales	Serratia proteamaculans 568	Spro_0076	COG1280 (RhtB)
YE4144	YE4144 - Enterobacteriales	Erwinia carotovora subsp. atroseptica SCRI1043	ECA3308	COG1280 (RhtB)
YE4144	AB57_2651 - Moraxellaceae	Acinetobacter baumannii AB0057	AB57_2651	COG384 (PhzF)
YE4144	Sbal_1936 - Shewanellaceae	Shewanella baltica OS155	Sbal_1936	COG384 (PhzF), COG697 (RhaT)
YE4144	Smlt0590 - Xanthomonadales	Stenotrophomonas maltophilia K279a	Smlt0590	TF
H16_A0970	BAV2320 - Alcaligenaceae	Bordetella avium 197N	BAV2320	hypothetical protein, TF
H16_A0970	BAV2320 - Alcaligenaceae	Bordetella bronchiseptica RB50	BB2054	hypothetical protein
H16_A0970	BAV2320 - Alcaligenaceae	Bordetella petrii DSM 12804	Bpet2044	hypothetical protein, TF
H16_A0970	Bamb_3558 - Burkholderia	Burkholderia cepacia AMMD	Bamb_3558	hypothetical protein
H16_A0970	Bamb_3558 - Burkholderia	Burkholderia phymatum STM815	Bphy_3121	hypothetical protein
H16_A0970	Bamb_3558 - Burkholderia	Burkholderia sp. 383	Bcep18194_B1880	hypothetical protein
H16_A0970	Bamb_3558 - Burkholderia	Burkholderia vietnamiensis G4	Bcep1808_4710	hypothetical protein
H16_A0970	Aave_3430 - Comamonadaceae	Acidovorax avenae subsp. citrulli AAC00-1	Aave_3430	hypothetical protein, TF
H16_A0970	Aave_3430 - Comamonadaceae	Comamonas testosteroni KF-1	CtesDRAFT_4867	hypothetical protein, TF
H16_A0970	Aave_3430 - Comamonadaceae	Delftia acidovorans SPH-1	Daci_5367	hypothetical protein, TF
H16_A0970	Aave_3430 - Comamonadaceae	Polaromonas naphthalenivorans CJ2	Pnap_3431	hypothetical protein, TF
H16_A0970	Aave_3430 - Comamonadaceae	Polaromonas sp. JS666	Bpro_2661	hypothetical protein, TF
H16_A0970	Aave_3430 - Comamonadaceae	Variovorax paradoxus S110	Vapar_0983	hypothetical protein, TF
H16_A0970	H16_A0970 - Ralstonia	Cupriavidus taiwanensis	RALTA_A0963	COG2509, hypothetical protein
H16_A0970	H16_A0970 - Ralstonia	Ralstonia eutropha H16	H16_A0970	COG2509, hypothetical protein, TF
H16_A0970	H16_A0970 - Ralstonia	Ralstonia eutropha JMP134	Reut_A2460	COG2509, TF
H16_A0970	H16_A0970 - Ralstonia	Ralstonia metallidurans CH34	Rmet_0868	COG2509
H16_A0970	H16_A0970 - Ralstonia	Ralstonia pickettii 12J	Rpic_0528	hypothetical protein
H16_A0970	H16_A0970 - Ralstonia	Ralstonia solanacearum GMI1000	RS04846 (RSc0599)	hypothetical protein
H16_A0970	Daro_1932 - Various betaproteobacteria	Laribacter hongkongensis HLHK9	LHK_00949	hypothetical protein
H16_A0970	Daro_1932 - Various betaproteobacteria	Dechloromonas aromatica RCB	Daro_1932	PF11142
YjiR2	YjiR2 - Oceanospirillales/Alteromonadales	Cellvibrio japonicus Ueda107	CJA_1192	COG1280 (RhtB)
YjiR2	YjiR2 - Oceanospirillales/Alteromonadales	Hahella chejuensis KCTC 2396	HCH_06834	COG115 (IlvE)
YjiR2	YjiR2 - Oceanospirillales/Alteromonadales	Marinobacter aqueolei	Maqu_2096	COG384 (PhzF)
YjiR2	YjiR2 - Oceanospirillales/Alteromonadales	Marinobacter sp. ELB17	MELB17_07959	COG384 (PhzF)
YjiR2	YjiR2 - Oceanospirillales/Alteromonadales	Saccharophagus degradans 2-40	Sde_3059	COG384 (PhzF), COG115 (IlvE)
YjiR	YjiR - Enterobacteriales	Citrobacter koseri ATCC BAA-895	CKO_03450	COG5457
YjiR	YjiR - Enterobacteriales	Enterobacter sp. 638	Ent638_0494	COG5457
YjiR	YjiR - Enterobacteriales	Erwinia carotovora subsp. atroseptica SCRI1043	ECA0227	COG5457
YjiR	YjiR - Enterobacteriales	Escherichia coli str. K-12 substr. MG1655	b4340	COG5457
YjiR	YjiR - Enterobacteriales	Klebsiella pneumoniae subsp. pneumoniae MGH 7	KPN_04760	
YjiR	YjiR - Enterobacteriales	Serratia proteamaculans 568	Spro_1440	COG5457
YjiR	YjiR - Oceanospirillales/Alteromonadales	Hahella chejuensis KCTC 2396	HCH_00190	COG5457, COG1280 (RhtB)
YjiR	YjiR - Oceanospirillales/Alteromonadales	Marinomonas sp. MWYL1	Mmwyl1_1450	COG1280 (RhtB)
YjiR	YjiR - Oceanospirillales/Alteromonadales	Reinekea sp. MED297	MED297_00535	COG1280 (RhtB), hypothetical protein
YjiR	YjiR - Oceanospirillales/Alteromonadales	Oceanospirillum sp. MED92	MED92_02813	hypothetical protein
YjiR	YjiR - Oceanospirillales/Alteromonadales	Marinobacter sp. ELB17	MELB17_00760	COG1280 (RhtB)*2
YjiR	YjiR - Pseudomonadaceae	Pseudomonas aeruginosa PAO1	PA2032	COG5457
YjiR	YjiR - Pseudomonadaceae	Pseudomonas entomophila L48	PSEEN5421	COG5457
YjiR	YjiR - Pseudomonadaceae	Pseudomonas fluorescens Pf-5	PFL_6043	COG5457
YjiR	YjiR - Pseudomonadaceae	Pseudomonas mendocina ymp	Pmen_0240	COG5457
YjiR	YjiR - Pseudomonadaceae	Pseudomonas putida KT2440	PP5275	COG5457
YjiR	YjiR - Pseudomonadaceae	Pseudomonas syringae pv. tomato str. DC3000	PSPTO0096	COG5457
Pcar_1599	Gbem_0803 - Desulfuromonadales	Geobacter sp. M21	GM21_0850	COG463 (WcaA)
Pcar_1599	Gbem_0803 - Desulfuromonadales	Geobacter sulfurreducens PCA	GSU0018	COG1357
Pcar_1599	Gbem_0803 - Desulfuromonadales	Geobacter uraniumreducens Rf4	Gura_2450	COG599, COG2814 (AraJ), hypothetical protein
Pcar_1599	Gbem_0803 - Desulfuromonadales	Geobacter sp. FRC-32	Geob_1843	hypothetical protein

Pcar_1599	Gbem_0803 - Desulfuromonadales	Pelobacter carbinolicus str. DSM 2380	Pcar_1599	COG714, COG3864, hypothetical protein, TF
Vapar_1337	Bcep18194_A4479 - Burkholderia	Burkholderia sp. 383	Bcep18194_A4479	COG436, COG456 (RimI)
Vapar_1337	Vapar_1337 - Comamonadaceae	Variovorax paradoxus S110	Vapar_1337	COG436
Cpin_4810	BACOVA_03038 - Bacteroidaceae	Bacteroides ovatus ATCC 8483	BACOVA_03038	COG1167 (ARO8)
Cpin_4810	Fjoh_0145 - Flavobacteria	Flavobacterium johnsoniae UW101	Fjoh_3396	COG2962 (RarD)
Cpin_4810	Fjoh_0145 - Flavobacteria	Flavobacterium johnsoniae UW101	Fjoh_0145	COG456 (RimI), hypothetical protein, TF
Cpin_4810	Cpin_4810 - Sphingobacteria	Chitinophaga pinensis DSM 2588	Cpin_4810	PF00583
Cpin_4810	Cpin_4810 - Sphingobacteria	Chitinophaga pinensis DSM 2588	Cpin_2921	COG697 (RhaT), COG76 (GadB)
Cpin_4810	Cpin_4810 - Sphingobacteria	Dyadobacter fermentans DSM 18053	Dfer_1184	COG697 (RhaT), COG76 (GadB), PF07609
Cpin_4810	Cpin_4810 - Sphingobacteria	Dyadobacter fermentans DSM 18053	Dfer_0750	COG1893 (ApbA), COG1028 (FabG)
Cpin_4810	Cpin_4810 - Sphingobacteria	Microscilla marina ATCC 23134	M23134_05128	COG76 (GadB)
Cpin_4810	Cpin_4810 - Sphingobacteria	Pedobacter heparinus DSM 2366	Phep_3795	hypothetical protein, TF
sll5086	sll5086 - Cyanobacteria	Synechococcus elongatus PCC 7942	Synpcc7942_1725	hypothetical protein
sll5086	sll5086 - Cyanobacteria	Synechocystis sp. PCC 6803	sll5086	COG5502
YdcR	YdcR - Alcaligenaceae	Bordetella bronchiseptica RB50	BB1241	COG1271 (CydA), COG1294 (CydB/AppB), PF10617, COG2863, COG2010 (CccA), TF
YdcR	YdcR - Burkholderia	Burkholderia cepacia AMMD	Bamb_4500	COG823 (TolB)
YdcR	YdcR - Burkholderia	Burkholderia cepacia AMMD	Bamb_3927	COG155 (CysI), COG3749, PF11174
YdcR	YdcR - Burkholderia	Burkholderia mallei ATCC 23344	BMA3050	COG155 (CysI), COG3749
YdcR	YdcR - Burkholderia	Burkholderia phymatum STM815	Bphy_6904	COG2200 (Rtn), COG2199
YdcR	YdcR - Burkholderia	Burkholderia phymatum STM815	Bphy_6902	COG4943, TF
YdcR	YdcR - Burkholderia	Burkholderia phymatum STM815	Bphy_3581	COG155 (CysI), COG3749, PF11174
YdcR	YdcR - Burkholderia	Burkholderia pseudomallei K96243	BPSL1321	COG155 (CysI), COG3749, PF11174
YdcR	YdcR - Burkholderia	Burkholderia sp. 383	Bcep18194_B0568	COG823 (TolB)
YdcR	YdcR - Burkholderia	Burkholderia sp. 383	Bcep18194_B1356	COG155 (CysI), COG3749, PF11174
YdcR	YdcR - Burkholderia	Burkholderia vietnamiensis G4	Bcep1808_5040	COG155 (CysI), COG3749, PF11174
YdcR	YdcR - Burkholderia	Burkholderia xenovorans LB400	Bxe_B0924	COG155 (CysI), COG3749, PF11174
YdcR	YdcR - Comamonadaceae	Delftia acidovorans SPH-1	Daci_2454	COG348 (NapH)
YdcR	YdcR - Comamonadaceae	Leptothrix cholodnii SP-6	Lcho_0726	COG348 (NapH), COG2863, COG3258, COG1960 (CaiA)
YdcR	YdcR - Comamonadaceae	Methylibium petroleiphilum PM1	Mpe_A0752	COG2863, COG2010 (CccA), PF11174
YdcR	YdcR - Comamonadaceae	Methylibium petroleiphilum PM1	Mpe_A2446	
YdcR	YdcR - Comamonadaceae	Polaromonas naphthalenivorans CJ2	Pnap_2093	COG348 (NapH), COG2863, COG2010 (CccA)
YdcR	YdcR - Comamonadaceae	Variovorax paradoxus S110	Vapar_4651	COG2863, COG2010 (CccA), PF11174
YdcR	YdcR - Comamonadaceae	Polaromonas sp. JS666	Bpro_3442	COG2863, COG2010 (CccA)
YdcR	YdcR - Ralstonia	Cupriavidus taiwanensis	RALTA_A1516	AdhA, AdhB, PF11174, COG2124 (CypX), PF10617, COG1271 (CydA), COG1294 (CydB/AppB)
YdcR	YdcR - Ralstonia	Ralstonia eutropha H16	H16_A1589	AdhA, AdhB, PF11174, PF10617, COG1271 (CydA), COG1294 (CydB/AppB)
YdcR	YdcR - Ralstonia	Ralstonia eutropha JMP134	Reut_B3939	AdhA, AdhB, PF11174, COG1271 (CydA), COG1294 (CydB/AppB)
YdcR	YdcR - Various betaproteobacteria	Chromobacterium violaceum ATCC 12472	CV1451	COG348 (NapH), TF
YdcR	YdcR - Various betaproteobacteria	Thiobacillus denitrificans	Tbd_1972	COG1845 (CyoC), hypothetical protein
YdcR	YdcR - Various betaproteobacteria	Methylotenera mobilis JLW8	Mmol_1442	COG348 (NapH), COG155 (CysI), COG3749
YdcR	YdcR - Various betaproteobacteria	Methylobacillus flagellatus KT	Mfla_1892	COG348 (NapH)*2
YdcR	YdcR - Various betaproteobacteria	Laribacter hongkongensis HLHK9	LHK_02098	COG348 (NapH)
YdcR	YdcR - Various betaproteobacteria	Dechloromonas aromatica RCB	Daro_1063	COG1845 (CyoC)*2, COG348 (NapH), hypothetical protein*2
YdcR	YdcR - Various betaproteobacteria	Thauera sp. MZ1T	Tmz1t_2226	COG1845 (CyoC), COG348 (NapH), hypothetical protein
YdcR	YdcR - Enterobacteriales	Citrobacter koseri ATCC BAA-895	CKO_01477	PF10777 (ylaC)
YdcR	YdcR - Enterobacteriales	Enterobacter sp. 638	Ent638_2129	PF10777 (ylaC), COG1271 (CydA), COG1294 (CydB/AppB)
YdcR	YdcR - Enterobacteriales	Erwinia carotovora subsp. atroseptica SCRI1043	ECA3004	PF10777 (ylaC)
YdcR	YdcR - Enterobacteriales	Erwinia amylovora ATCC 49946	EAM_2947	PF10777 (ylaC)
YdcR	YdcR - Enterobacteriales	Escherichia coli str. K-12 substr. MG1655	b1439	PF10777 (ylaC)
YdcR	YdcR - Enterobacteriales	Klebsiella pneumoniae subsp. pneumoniae MGH 7	KPN_01929	PF10777 (ylaC), COG1271 (CydA), COG1294 (CydB/AppB)
YdcR	YdcR - Enterobacteriales	Photorhabdus luminescens subsp. laumondii TTO1	plu2044	COG1271 (CydA), COG1294 (CydB/AppB)
YdcR	YdcR - Enterobacteriales	Proteus mirabilis HI4320	PMI1433	COG1271 (CydA), COG1294 (CydB/AppB)
YdcR	YdcR - Enterobacteriales	Salmonella typhimurium LT2	STM1598	PF10777 (ylaC)
YdcR	YdcR - Enterobacteriales	Serratia proteamaculans 568	Spro_2858	PF10777 (ylaC), COG1271 (CydA), COG1294 (CydB/AppB)
YdcR	YdcR - Enterobacteriales	Yersinia pestis KIM 10	y2350	
YdcR	YdcR - Moraxellaceae	Acinetobacter baumannii AB0057	AB57_1664	COG1271 (CydA), COG1294 (CydB/AppB), hypothetical protein
YdcR	YdcR - Moraxellaceae	Acinetobacter sp. ADP1	ACIAD2032	hypothetical protein
YdcR	YdcR - Oceanospirillales/Alteromonadales	Hahella chejuensis KCTC 2396	HCH_03523	COG348 (NapH)
YdcR	YdcR - Oceanospirillales/Alteromonadales	Cellvibrio japonicus Ueda107	CJA_3711	COG348 (NapH), TF
YdcR	YdcR - Pseudomonadaceae	Pseudomonas aeruginosa PAO1	PA4132	COG3278 (CcoN), COG155 (CysI), COG3749
YdcR	YdcR - Pseudomonadaceae	Pseudomonas entomophila L48	PSEEN2988	COG348 (NapH), TF
YdcR	YdcR - Pseudomonadaceae	Pseudomonas fluorescens Pf-5	PFL_2868	COG2303 (BetA/GadH1), COG2010 (CccA/GadH2), GadH3 (Pfam13618), COG3278 (CcoN), COG155 (CysI), COG3749, COG348 (NapH), TF
YdcR	YdcR - Pseudomonadaceae	Pseudomonas mendocina ymp	Pmen_3095	COG348 (NapH), TF
YdcR	YdcR - Pseudomonadaceae	Pseudomonas putida KT2440	PP3544	COG348 (NapH), TF
YdcR	YdcR - Pseudomonadaceae	Pseudomonas stutzeri A1501	PST_2731	COG348 (NapH), TF
YdcR	YdcR - Psychromonadaceae/Aeromonadales	Aeromonas hydrophila subsp. hydrophila ATCC 79	AHA_1639	COG1271 (CydA), COG1294 (CydB/AppB)
YdcR	YdcR - Psychromonadaceae/Aeromonadales	Aeromonas salmonicida subsp. salmonicida A449	ASA_2722	COG1271 (CydA), COG1294 (CydB/AppB)
YdcR	YdcR - Shewanellaceae	Shewanella baltica OS155	Sbal_0915	COG1271 (CydA), COG1294 (CydB/AppB)
YdcR	YdcR - Shewanellaceae	Shewanella putrefaciens CN-32	Sputcn32_0931	COG1271 (CydA), COG1294 (CydB/AppB)
YdcR	YdcR - Shewanellaceae	Shewanella sp ANA-3	Shewana3_0929	COG1271 (CydA), COG1294 (CydB/AppB)
YdcR	YdcR - Shewanellaceae	Shewanella sp MR-7	Shewmr7_0965	COG1271 (CydA), COG1294 (CydB/AppB)
YdcR	YdcR - Shewanellaceae	Shewanella sp W3-18-1	Sputw3181_3245	COG1271 (CydA), COG1294 (CydB/AppB)
YdcR	YdcR - Vibrionales	Vibrio cholerae O1 biovar eltor str. N16961	VCA0871	COG1271 (CydA), COG1294 (CydB/AppB)
YdcR	YdcR - Vibrionales	Vibrio harveyi ATCC BAA-1116	VIBHAR_01894	hypothetical protein, COG348 (NapH)
YdcR	YdcR - Vibrionales	Vibrio parahaemolyticus RIMD 2210633	VPA1136	COG1271 (CydA), COG1294 (CydB/AppB)
YdcR	YdcR - Vibrionales	Vibrio parahaemolyticus RIMD 2210633	VP1896	hypothetical protein, COG348 (NapH)
YdcR	YdcR - Vibrionales	Vibrio splendidus LGP32	VS_1691	COG1271 (CydA), COG1294 (CydB/AppB)
YdcR	YdcR - Vibrionales	Vibrio splendidus LGP32	VS_1814	hypothetical protein, COG348 (NapH)
YdcR	YdcR - Vibrionales	Vibrio vulnificus CMCP6	VV1_2252	hypothetical protein, COG348 (NapH)
YdcR	YdcR - Vibrionales	Photobacterium profundum SS9	PBPRB1592	COG348 (NapH)
H16_B1468	BPSS0970 - Burkholderia	Burkholderia xenovorans LB400	Bxe_A1487	COG243 (BisC/FdhA)
H16_B1468	BPSS0970 - Burkholderia	Burkholderia sp. 383	Bcep18194_B1266	COG243 (BisC/FdhA), COG3781
H16_B1468	BPSS0970 - Burkholderia	Burkholderia cepacia AMMD	Bamb_4005	COG243 (BisC/FdhA), COG3781
H16_B1468	BPSS0970 - Burkholderia	Burkholderia vietnamiensis G4	Bcep1808_5103	COG243 (BisC/FdhA), COG3781
H16_B1468	BPSS0970 - Burkholderia	Burkholderia glumae BGR1	bglu_2g13870	COG243 (BisC/FdhA), COG3781
H16_B1468	BPSS0970 - Burkholderia	Burkholderia mallei ATCC 23344	BMAA1253	COG243 (BisC/FdhA), COG3781
H16_B1468	BPSS0970 - Burkholderia	Burkholderia pseudomallei K96243	BPSS0970	COG243 (BisC/FdhA), COG3781
H16_B1468	BPSS0970 - Burkholderia	Burkholderia phymatum STM815	Bphy_4744	COG243 (BisC/FdhA)
H16_B1468	H16_B1468 - Ralstonia	Ralstonia eutropha H16	H16_B1468	COG1271 (CydA), COG1294 (CydB/AppB), COG243 (BisC/FdhA), COG1526 (FdhD), COG2896 (MoaA), COG303 (MoeA),TF
H16_B1468	H16_B1468 - Ralstonia	Cupriavidus taiwanensis	RALTA_B1733	COG1271 (CydA), COG1294 (CydB/AppB), COG243 (BisC/FdhA), COG1526 (FdhD), COG2896 (MoaA), COG303 (MoeA),TF
H16_B1468	H16_B1468 - Ralstonia	Ralstonia eutropha JMP134	Reut_B4094	COG1271 (CydA), COG1294 (CydB/AppB), COG243 (BisC/FdhA), COG1526 (FdhD), COG303 (MoeA), TF
H16_B1468	H16_B1468 - Ralstonia	Ralstonia metallidurans CH34	Rmet_1126	COG243 (BisC/FdhA), COG1526 (FdhD), TF
H16_B1468	Mfla_0336 - Various betaproteobacteria	Methylobacillus flagellatus KT	Mfla_0336	COG243 (BisC/FdhA), hypothetical protein, TF
H16_B1468	Mfla_0336 - Various betaproteobacteria	Methylotenera mobilis JLW8	Mmol_0468	COG243 (BisC/FdhA), hypothetical protein, TF
Mmwyl1_0910	CPS_3393 - Alteromonadales	Colwellia psychrerythraea 34H	CPS_3393	COG1448 (TyrB), COG2962 (RarD)
Mmwyl1_0910	Spro_0112 - Enterobacteriales	Serratia proteamaculans 568	Spro_0112	SSF55729
Mmwyl1_0910	ACIAD3613 - Moraxellaceae	Acinetobacter baumannii AB0057	AB57_3892	hypothetical protein
Mmwyl1_0910	ACIAD3613 - Moraxellaceae	Acinetobacter sp. ADP1	ACIAD3613	hypothetical protein
Mmwyl1_0910	Mmwyl1_0910 - Oceanospirillales/Alteromonadales	Marinomonas sp. MWYL1	Mmwyl1_0910	COG2962 (RarD), COG1280 (RhtB)
Mmwyl1_0910	Mmwyl1_0910 - Oceanospirillales/Alteromonad	Hahella chejuensis KCTC 2396	HCH_00707	COG76 (GadB)
Mmwyl1_0910	PE36_10153 - Psychromonadaceae/Aeromonad	Moritella sp. PE36	PE36_10153	COG2962 (RarD)
Mmwyl1_0910	VV21354 - Vibrionales	Vibrio angustum S14	VAS14_03568	hypothetical protein
Mmwyl1_0910	VV21354 - Vibrionales	Vibrio splendidus LGP32	VS_1723	COG2962 (RarD)
Mmwyl1_0910	VV21354 - Vibrionales	Vibrio vulnificus CMCP6	VV2_1354	COG2962 (RarD)
Mmwyl1_0910	XAC0737 - Xanthomonadales	Xanthomonas axonopodis pv. citri str. 306	XAC0737	COG2962 (RarD), COG384 (PhzF)
Mmwyl1_0910	XAC0737 - Xanthomonadales	Xanthomonas campestris pv. campestris str. ATCC	XCC3386	COG2962 (RarD), COG384 (PhzF)
YdfD/YisV	YdfD/YisV - Bacillales	Bacillus cereus ATCC 14579	BC1987	COG1279
YdfD/YisV	YdfD/YisV - Bacillales	Bacillus cereus ATCC 14579	BC2680	COG697 (RhaT)
YdfD/YisV	YdfD/YisV - Bacillales	Bacillus clausii KSM-K16	ABC1201	COG1279
YdfD/YisV	YdfD/YisV - Bacillales	Bacillus halodurans C-125	BH0432	COG1279
YdfD/YisV	YdfD/YisV - Bacillales	Bacillus subtilis subsp. subtilis str. 168	BSU10880	COG1279
YdfD/YisV	YdfD/YisV - Bacillales	Bacillus subtilis subsp. subtilis str. 168	BSU05370	COG697 (RhaT)
YdfD/YisV	YdfD/YisV - Bacillales	Paenibacillus sp. JDR-2	Pjdr2_2789	COG1279
YdfD/YisV	YdfD/YisV - Clostridia-1	Clostridium beijerinckii NCIMB 8052	Cbei_2083	COG1279
YdfD/YisV	YdfD/YisV - Clostridia-1	Clostridium kluyveri DSM 555	CKL_2224	COG1279
YdfD/YisV	YdfD/YisV - Lactobacillaceae	Lactobacillus plantarum WCFS1	lp_1407	COG1279

YdfD/YisV	YdfD/YisV - Staphylococcaceae	Staphylococcus aureus subsp. aureus N315	SA0104	COG697 (RhaT)
YdfD/YisV	YdfD/YisV - Staphylococcaceae	Staphylococcus capitis SK14	STACA0001_1858	COG1279, COG3239 (DesA)
YdfD/YisV	YdfD/YisV - Staphylococcaceae	Staphylococcus carnosus subsp. carnosus TM300	Sca_0254	COG1279
YdfD/YisV	YdfD/YisV - Staphylococcaceae	Staphylococcus saprophyticus subsp. saprophyticu	SSP2041	COG1279
PdxR (Lactobacillales)	PdxR - Enterococcaceae	Enterococcus faecalis V583	EF2426	COG4720 (PdxU), COG351 (PdxK2)*2, COG4475, COG1670 (RimL) , TF
PdxR (Lactobacillales)	PdxR - Enterococcaceae	Enterococcus faecium DO	EfaeDRAFT_1355	COG4720 (PdxU), COG351 (PdxK2), COG4475
PdxR (Lactobacillales)	PdxR - Lactobacillaceae	Lactobacillus brevis ATCC 367	LVIS_2153	COG4720 (PdxU), COG351 (PdxK2), COG4475, TF
PdxR (Lactobacillales)	PdxR - Lactobacillaceae	Lactobacillus sakei subsp. sakei 23K	LSA1351	COG4720 (PdxU), COG351 (PdxK2), COG4475, TF
PdxR (Lactobacillales)	PdxR - Listeriaceae	Listeria innocua Clip11262	lin2204	COG214 (PdxS), COG311 (PdxT), TF
PdxR (Lactobacillales)	PdxR - Listeriaceae	Listeria monocytogenes EGD-e	lmo2100	COG214 (PdxS), COG311 (PdxT), TF
PdxR (Lactobacillales)	PdxR - Listeriaceae	Listeria seeligeri serovar 1/2b str. SLCC3954	lse_2090	COG214 (PdxS), COG311 (PdxT), TF
PdxR (Lactobacillales)	PdxR - Listeriaceae	Listeria welshimeri serovar 6b str. SLCC5334	lwe2121	COG214 (PdxS), COG311 (PdxT), TF
BC3039	BC3039 - Bacilliales	Bacillus cereus ATCC 14579	BC3039	COG697 (RhaT), TF
YcxD	YcxD - Bacillales	Bacillus amyloliquefaciens FZB42	RBAM_003730	COG697 (RhaT)
YcxD	YcxD - Bacillales	Bacillus licheniformis ATCC 14580	BLi00406	COG697 (RhaT)
YcxD	YcxD - Bacillales	Bacillus pumilus SAFR-032	BPUM_0326	COG697 (RhaT)
YcxD	YcxD - Bacillales	Bacillus subtilis subsp. subtilis str. 168	BSU03560	COG697 (RhaT)
YcxD	YcxD - Bacillales	Paenibacillus sp. JDR-2	Pjdr2_0546	COG697 (RhaT)
YcxD	YcxD - Clostridia-1	Clostridium botulinum A str. ATCC 3502	CBO2012	COG697 (RhaT)
YcxD	YcxD - Clostridia-1	Clostridium beijerinckii NCIMB 8052	Cbei_4035	COG1486 (CelF)
PdxR (Streptococcaceae)	PdxR - Streptococcaceae	Streptococcus dysgalactiae subsp. equisimilis GGS	SDEG_1023	COG2240 (PdxK), COG4720 (PdxU), TF
PdxR (Streptococcaceae)	PdxR - Streptococcaceae	Streptococcus equi subsp. zooepidemicus MGCS1C	Sez_0950	COG2240 (PdxK), COG4720 (PdxU), TF
PdxR (Streptococcaceae)	PdxR - Streptococcaceae	Streptococcus gallolyticus UCN34	GALLO_1111	COG2240 (PdxK), COG4720 (PdxU), TF
PdxR (Streptococcaceae)	PdxR - Streptococcaceae	Streptococcus gordonii str. Challis substr. CH1	SGO_0963	COG2240 (PdxK), COG4720 (PdxU), TF
PdxR (Streptococcaceae)	PdxR - Streptococcaceae	Streptococcus mutans UA159	SMU.953c	COG2240 (PdxK), COG4720 (PdxU), TF
PdxR (Streptococcaceae)	PdxR - Streptococcaceae	Streptococcus pyogenes M1 GAS	SPy1210	COG2240 (PdxK), COG4720 (PdxU), TF
PdxR (Streptococcaceae)	PdxR - Streptococcaceae	Streptococcus sanguinis SK36	SSA_1401	COG2240 (PdxK), COG4720 (PdxU), TF
PdxR (Thermoanaerobacterales)	PdxR - Thermoanaerobacterales	Anaerocellum thermophilum DSM 6725	Athe_1910	COG214 (PdxS), COG311 (PdxT), TF
PdxR (Thermoanaerobacterales)	PdxR - Thermoanaerobacterales	Caldicellulosiruptor saccharolyticus DSM 8903	Csac_1035	COG214 (PdxS), COG311 (PdxT), TF
Spro_3347	Spro_3347 - Enterobacteriales	Serratia proteamaculans 568	Spro_3347	COG5006 (rhtA)
Spro_3347	DVU2953 - Desulfovibrionales	Desulfomicrobium baculatum DSM 4028	Dbac_0439	COG5006 (rhtA)
Spro_3347	DVU2953 - Desulfovibrionales	Desulfovibrio vulgaris Hildenborough	DVU2953	COG5006 (rhtA)
PdxR2 (Actinobacteria)	PdxR2 - Frankineae/Propionibacterineae/Pseudc	Nakamurella multipartita DSM 44233	Namu_0648	COG3467 (PdxO)
PdxR2 (Actinobacteria)	PdxR2 - Frankineae/Propionibacterineae/Pseudc	Nocardioides sp. JS614	Noca_3468	COG3467 (PdxO)
PdxR2 (Actinobacteria)	PdxR2 - Frankineae/Propionibacterineae/Pseudc	Actinosynnema mirum DSM 43827	Amir_5991	COG3467 (PdxO), COG214 (PdxS)
PdxR2 (Actinobacteria)	PdxR2 - Frankineae/Propionibacterineae/Pseudc	Saccharomonospora viridis DSM 43017	Svir_09150	COG214 (PdxS)
PdxR2 (Actinobacteria)	PdxR2 - Frankineae/Propionibacterineae/Pseudc	Saccharopolyspora erythraea NRRL 2338	SACE_6131	COG3467 (PdxO), COG329 (DapA)
PdxR2 (Actinobacteria)	PdxR2(2) - Micrococcineae	Janibacter sp. HTCC2649	JNB_19088	COG3467 (PdxO)
PdxR2 (Actinobacteria)	PdxR2 - Mycobacteriaceae	Mycobacterium abscessus ATCC 19977	MAB_1353c	COG3467 (PdxO)
PdxR2 (Actinobacteria)	PdxR2 - Nocardiaceae	Rhodococcus sp. RHA1	RHA1_ro01235	COG3467 (PdxO)
PdxR2 (Actinobacteria)	PdxR2 - Nocardiaceae	Nocardia farcinica IFM 10152	nfa50160	COG3467 (PdxO)
PdxR2 (Actinobacteria)	PdxR2 - Nocardiaceae	Rhodococcus erythropolis PR4	RER_58480	COG3467 (PdxO)
PdxR2 (Actinobacteria)	PdxR2 - Nocardiaceae	Rhodococcus opacus B4	ROP_09570	COG3467 (PdxO)
PdxR2 (Actinobacteria)	PdxR2 - Deinococcus-Thermus	Deinococcus deserti VCD115	Deide_3p01060	COG3467 (PdxO), COG456 (RimI)
PdxR (Actinobacteria)	PdxR - Corynebacteriaceae	Corynebacterium aurimucosum ATCC 700975	cauri_0519	COG214 (PdxS), COG311 (PdxT)
PdxR (Actinobacteria)	PdxR - Corynebacteriaceae	Corynebacterium glutamicum R	cg0897	COG214 (PdxS), COG311 (PdxT)
PdxR (Actinobacteria)	PdxR - Corynebacteriaceae	Corynebacterium diphtheriae NCTC 13129	DIP0226	COG214 (PdxS), COG311 (PdxT)
PdxR (Actinobacteria)	PdxR - Frankineae/Propionibacterineae/Pseudor	Propionibacterium acnes KPA171202	PPA0964	COG214 (PdxS), COG311 (PdxT)
PdxR (Actinobacteria)	PdxR - Micrococcineae	Kocuria rhizophila DC2201	KRH_01150	COG214 (PdxS), COG311 (PdxT)
PdxR (Actinobacteria)	PdxR - Micrococcineae	Jonesia denitrificans DSM 20603	Jden_1599	COG214 (PdxS), COG311 (PdxT)
PdxR (Actinobacteria)	PdxR - Micrococcineae	Brevibacterium linens BL2	BlinB01003387	COG214 (PdxS), COG311 (PdxT)
SGR_2706	SACE_6928 - Frankineae/Propionibacterineae/Ps	Nakamurella multipartita DSM 44233	Namu_4809	COG697 (RhaT)
SGR_2706	SACE_6928 - Frankineae/Propionibacterineae/Ps	Saccharopolyspora erythraea NRRL 2338	SACE_6928	COG697 (RhaT)
SGR_2706	SACE_6928 - Frankineae/Propionibacterineae/Ps	Saccharopolyspora erythraea NRRL 2338	SACE_6850	COG329 (DapA)
SGR_2706	Bcav_0410 - Micrococcineae	Beutenbergia cavernae DSM 12333	Bcav_0410	hypothetical protein*3
SGR_2706	MSMEG_4140 - Mycobacteriaceae	Mycobacterium abscessus ATCC 19977	MAB_0196c	COG2128
SGR_2706	MSMEG_4140 - Mycobacteriaceae	Mycobacterium smegmatis str. MC2 155	MSMEG_4140	COG1087 (GalE), hypothetical protein
SGR_2706	MSMEG_4140 - Mycobacteriaceae	Mycobacterium smegmatis str. MC2 155	MSMEG_6371	COG2128
SGR_2706	MSMEG_4140 - Mycobacteriaceae	Mycobacterium smegmatis str. MC2 155	MSMEG_5760	COG1917
SGR_2706	MSMEG_4140 - Mycobacteriaceae	Mycobacterium smegmatis str. MC2 155	MSMEG_1572	COG697 (RhaT)
SGR_2706	MSMEG_4140 - Mycobacteriaceae	Mycobacterium vanbaalenii PYR-1	Mvan_5625	COG2128
SGR_2706	MSMEG_4140 - Mycobacteriaceae	Mycobacterium sp. JLS	Mjls_5379	COG2128, COG1917
SGR_2706	MSMEG_4140 - Mycobacteriaceae	Mycobacterium gilvum PYR-GCK	Mflv_1181	COG2128
SGR_2706	Nfa21130 - Nocardiaceae	Nocardia farcinica IFM 10152	nfa21130	COG2128
SGR_2706	Nfa21130 - Nocardiaceae	Rhodococcus erythropolis PR4	RER_05310	COG2128
SGR_2706	Nfa21130 - Nocardiaceae	Rhodococcus opacus B4	ROP_51330	COG2128
SGR_2706	Nfa21130 - Nocardiaceae	Rhodococcus sp. RHA1	RHA1_ro05072	COG2128
SGR_2706	SCO4836 - Streptomycetaceae	Streptomyces avermitilis MA-4680	SAV_3427	COG2128
SGR_2706	SCO4836 - Streptomycetaceae	Streptomyces avermitilis MA-4680	SAV_6256	COG2128
SGR_2706	SCO4836 - Streptomycetaceae	Streptomyces avermitilis MA-4680	SAV_7065	COG697 (RhaT)
SGR_2706	SCO4836 - Streptomycetaceae	Streptomyces avermitilis MA-4680	SAV_4231	
SGR_2706	SCO4836 - Streptomycetaceae	Streptomyces coelicolor A3(2)	SCO4836	COG2128*2
SGR_2706	SCO4836 - Streptomycetaceae	Streptomyces coelicolor A3(2)	SCO1289	COG697 (RhaT)
SGR_2706	SCO4836 - Streptomycetaceae	Streptomyces griseus subsp. griseus NBRC 13350	SGR_2706	COG2128
SGR_2706	SCO4836 - Streptomycetaceae	Streptomyces griseus subsp. griseus NBRC 13350	SGR_5306	COG2128
SGR_2706	SCO4836 - Streptomycetaceae	Streptomyces griseus subsp. griseus NBRC 13350	SGR_6261	COG697 (RhaT)
SGR_2706	SCO4836 - Streptomycetaceae	Streptomyces scabiei 87.22	SCAB_69291	COG2128
SGR_2706	SCO4836 - Streptomycetaceae	Streptomyces scabiei 87.22	SCAB_77981	
Bcav_0044	Bcav_0044 - Micrococcineae	Beutenbergia cavernae DSM 12333	Bcav_0044	COG667 (Tas)
PA0268	BPSS1200 - Burkholderia	Burkholderia cepacia AMMD	Bamb_3694	COG2128
PA0268	BPSS1200 - Burkholderia	Burkholderia phymatum STM815	Bphy_4510	COG2128
PA0268	BPSS1200 - Burkholderia	Burkholderia pseudomallei K96243	BPSS1200	COG2128, COG1917
PA0268	BPSS1200 - Burkholderia	Burkholderia sp. 383	Bcep18194_B1740	COG2128, COG1917
PA0268	BPSS1200 - Burkholderia	Burkholderia vietnamiensis G4	Bcep1808_4832	COG2128, COG1917
PA0268	BPSS1200 - Burkholderia	Burkholderia xenovorans LB400	Bxe_B0760	COG2128, COG1917, COG1182 (AcpD)
PA0268	H16_A0963 - Ralstonia	Cupriavidus taiwanensis	RALTA_A0957	COG2128
PA0268	H16_A0963 - Ralstonia	Ralstonia eutropha H16	H16_A0963	COG2128
PA0268	H16_A0963 - Ralstonia	Ralstonia eutropha JMP134	Reut_A1805	COG2128
PA0268	H16_A0963 - Ralstonia	Ralstonia metallidurans CH34	Rmet_4318	COG2128, COG1917
PA0268	H16_A0963 - Ralstonia	Ralstonia pickettii 12J	Rpic_3611	COG2128
PA0268	PA0268 - Pseudomonadaceae	Pseudomonas aeruginosa PAO1	PA0268	COG2128, COG1917
PA0268	PA0268 - Pseudomonadaceae	Pseudomonas entomophila L48	PSEEN3192	COG2128
PA0268	PA0268 - Pseudomonadaceae	Pseudomonas mendocina ymp	Pmen_1085	COG2128, COG5006 (rhtA)
Spro_3719	Bamb_5788 - Burkholderia	Burkholderia glumae BGR1	bglu_2g11570	COG451 (WcaG)
Spro_3719	Bamb_5788 - Burkholderia	Burkholderia glumae BGR1	bglu_2g19100	COG1182 (AcpD), PF00583
Spro_3719	Bamb_5788 - Burkholderia	Burkholderia sp. 383	Bcep18194_B3074	COG1182 (AcpD), PF00583
Spro_3719	Bamb_5788 - Burkholderia	Burkholderia cepacia AMMD	Bamb_5788	COG451 (WcaG)
Spro_3719	Vapar_5644 - Comamonadaceae	Verminephrobacter eiseniae EF01-2	Veis_1588	COG2128, PF07681
Spro_3719	Vapar_5644 - Comamonadaceae	Variovorax paradoxus S110	Vapar_5644	COG1917, COG1087 (GalE)
Spro_3719	H16_B1411 - Ralstonia	Ralstonia eutropha JMP134	Reut_A1580	COG702, COG1917
Spro_3719	H16_B1411 - Ralstonia	Ralstonia eutropha H16	H16_B1411	PF00583, COG702, COG2128, COG1917, COG596 (MhpC), COG1335 (PncA)
Spro_3719	CV1517 - Various betaproteobacteria	Chromobacterium violaceum ATCC 12472	CV1517	COG2128, COG2513 (PrpB)
Spro_3719	Spro_3719 - Enterobacteriales	Citrobacter koseri ATCC BAA-895	CKO_04020	COG2128
Spro_3719	Spro_3719 - Enterobacteriales	Enterobacter sp. 638	Ent638_0497	COG2128
Spro_3719	Spro_3719 - Enterobacteriales	Erwinia carotovora subsp. atroseptica SCRI1043	ECA2654	COG2128
Spro_3719	Spro_3719 - Enterobacteriales	Erwinia carotovora subsp. atroseptica SCRI1043	ECA3744	PF00583
Spro_3719	Spro_3719 - Enterobacteriales	Klebsiella pneumoniae subsp. pneumoniae MGH 7	KPN_03002	COG2128
Spro_3719	Spro_3719 - Enterobacteriales	Salmonella typhimurium LT2	STM2803	COG2128
Spro_3719	Spro_3719 - Enterobacteriales	Serratia proteamaculans 568	Spro_3719	COG2128
Spro_3719	HCH_03332 - Oceanospirillales/Alteromonadale	Hahella chejuensis KCTC 2396	HCH_03332	COG1182 (AcpD), COG1225 (Bcp), COG4232
Spro_3719	PFL_2191 - Pseudomonadaceae	Pseudomonas fluorescens Pf-5	PFL_2191	COG2128, COG1182 (AcpD), PF00583
Spro_3719	Sbal_4072 - Shewanellaceae	Shewanella baltica OS155	Sbal_4072	COG1182 (AcpD), COG1225 (Bcp), COG4232, PF00583
blr6977	blr6977 - Rhizobiales	Bradyrhizobium japonicum USDA 110	blr6977	COG2128, COG1917
blr6977	Swit_4344 - Sphingomonadales	Sphingomonas wittichii RW1	Swit_4344	COG4117, COG2041
blr6977	Bpet3002 - Alcaligenaceae	Bordetella petrii DSM 12804	Bpet3002	COG2128, COG1917
blr6977	Bamb_6386 - Burkholderia	Burkholderia cepacia AMMD	Bamb_6386	COG2128, COG1917, PF01966

blr6977	Bamb_6386 - Burkholderia	Burkholderia glumae BGR1	bglu_2g19020	COG2128, COG1917
blr6977	Bamb_6386 - Burkholderia	Burkholderia phymatum STM815	Bphy_6987	COG2128, COG1917, COG1028 (FabG), PF01966
blr6977	Bamb_6386 - Burkholderia	Burkholderia phymatum STM815	Bphy_3552	COG2128, COG1917, COG1028 (FabG), PF01966
blr6977	Bpro_5165 - Comamonadaceae	Polaromonas sp. JS666	Bpro_5165	COG1573, COG3189
blr6977	H16_B0275 - Ralstonia	Ralstonia eutropha H16	H16_B0275	COG2128, COG1917
blr6977	H16_B0275 - Ralstonia	Ralstonia eutropha JMP134	Reut_C6135	COG2128, COG1917, COG1028 (FabG), PF01966
blr6977	EbA483 - Various Betaproteobacteria	Azoarcus sp. EbN1	ebA6755	COG2128*2
blr6977	EbA483 - Various Betaproteobacteria	Azoarcus sp. EbN1	ebA483	COG2128*2
SPOA0379	SPOA0379 - Rhodobacterales	Silicibacter pomeroyi DSS-3	SPOA0379	COG4948, COG665 (DadA), COG3842 (PotA), COG1176 (PotB), COG1177 (PotC), COG687 (PotD)
SPOA0379	SPOA0379 - Rhodobacterales	Roseovarius sp. 217	ROS217_07305	COG4948, COG665 (DadA), COG3842 (PotA), COG1176 (PotB), COG1177 (PotC), COG687 (PotD), TF
SPOA0379	Veis_4721 - Comamonadaceae	Verminephrobacter eiseniae EF01-2	Veis_4721	COG687 (PotD)*2
Veis_0044	Mll2434 - Rhizobiales	Mesorhizobium loti MAFF303099	mll2434	COG1082 (IolE), COG673 (MviM)
Veis_0044	Mll2434 - Rhizobiales	Bradyrhizobium japonicum USDA 110	blr2901	COG697 (RhaT)
Veis_0044	Veis_0044 - Comamonadaceae	Verminephrobacter eiseniae EF01-2	Veis_0044	COG1082 (IolE)*2, COG1879 (RbsB), COG1172 (AraH), COG1129, COG2120, COG673 (MviM), TF
PdxW2 (Comamonadaceae)	PdxW2 - Comamonadaceae	Leptothrix cholodnii SP-6	Lcho_0025	COG456 (RimI), COG2808 (PdxO2), COG1764 (OsmC)
RHA1_ro08093	RHA1_ro08093 - Nocardiaceae	Rhodococcus sp. RHA1	RHA1_ro08093	COG1012 (PutA), PF05899*2, PF03992*2, hypothetical protein
PdxW (Rhodospirillales)	PdxW - Rhodospirillales	Magnetospirillum magneticum AMB-1	amb1376	COG3467 (PdxO)
PdxW (Rhodospirillales)	PdxW - Rhodospirillales	Magnetospirillum magnetotacticum MS-1	Magn03008368	COG3467 (PdxO)
PdxW (Rhodobacterales)	PdxW - Rhodobacterales	Roseovarius nubinhibens ISM	ISM_11775	COG3467 (PdxO)
PdxW (Alphaproteobacteria)	PdxW - Caulobacterales	Phenylobacterium zucineum HLK1	PHZ_c0123	COG3467 (PdxO), COG1670 (RimL)
PdxW (Alphaproteobacteria)	PdxW - Rhizobiales	Azorhizobium caulinodans ORS 571	AZC_0405	COG3467 (PdxO)
PdxW (Alphaproteobacteria)	PdxW - Rhizobiales	Mesorhizobium loti MAFF303099	mlr1821	COG3467 (PdxO)
PdxW (Alphaproteobacteria)	PdxW - Rhizobiales	Bradyrhizobium japonicum USDA 110	bll4154	COG3467 (PdxO)
PdxW (Alphaproteobacteria)	PdxW - Rhizobiales	Bradyrhizobium sp. BTAi1	BBta_6494	COG3467 (PdxO)
PdxR2 (Proteobacteria)	PdxR2 - Alcaligenaceae	Bordetella avium 197N	BAV1602	COG325, COG2240 (PdxK)
PdxR2 (Proteobacteria)	PdxR2 - Alcaligenaceae	Bordetella bronchiseptica RB50	BB2872	COG325, COG2240 (PdxK), TF
PdxR2 (Proteobacteria)	PdxR2 - Ralstonia	Ralstonia metallidurans CH34	Rmet_4117	COG325, COG2240 (PdxK), pdxZ, TF
PdxR2 (Proteobacteria)	PdxR2 - Enterobacteriales	Citrobacter koseri ATCC BAA-895	CKO_00371	COG2240 (PdxK), pdxZ, COG518 (GuaA), TF
PdxR2 (Proteobacteria)	PdxR2 - Enterobacteriales	Enterobacter sp. 638	Ent638_4213	COG2240 (PdxK), COG518 (GuaA), COG325, TF
PdxR2 (Proteobacteria)	PdxR2 - Enterobacteriales	Klebsiella pneumoniae subsp. pneumoniae MGH 7	KPN_02767	COG2240 (PdxK), pdxZ, TF
PdxR2 (Proteobacteria)	PdxR2 - Enterobacteriales	Salmonella typhimurium LT2	STM2436	COG2240 (PdxK), pdxZ, COG518 (GuaA), TF
PdxR2 (Proteobacteria)	PdxR2 - Enterobacteriales	Serratia proteamaculans 568	Spro_2563	COG2240 (PdxK), pdxZ, COG518 (GuaA), TF
PdxR2 (Proteobacteria)	PdxR2 - Xanthomonadales	Stenotrophomonas maltophilia K279a	Smlt2930	COG325, COG518 (GuaA), TF
PdxR2 (Proteobacteria)	PdxR2 - Micrococcineae	Brachybacterium faecium DSM 4810	Bfae_00360	COG214 (PdxS), TF
PdxR2 (Proteobacteria)	PdxR2 - Micrococcineae	Leifsonia xyli subsp. xyli str. CTCB07	Lxx10730	COG214 (PdxS), COG311 (PdxT), COG2240 (PdxK), TF
PdxW (Actinobacteria)	PdxW - Mycobacteriaceae	Mycobacterium avium 104	MAV_3214	COG3467 (PdxO), TF
PdxW (Actinobacteria)	PdxW - Mycobacteriaceae	Mycobacterium marinum M	MMAR_2376	COG3467 (PdxO), TF
PdxW (Actinobacteria)	PdxW - Streptomycetaceae	Streptomyces avermitilis MA-4680	SAV_7000	COG3467 (PdxO), TF
PdxW (Actinobacteria)	PdxW - Streptomycetaceae	Streptomyces avermitilis MA-4680	SAV_6971	TF
PdxW (Actinobacteria)	PdxW - Streptomycetaceae	Streptomyces coelicolor A3(2)	SCO1360	COG3467 (PdxO), TF
PdxW (Actinobacteria)	PdxW - Streptomycetaceae	Streptomyces griseus subsp. griseus NBRC 13350	SGR_6172	COG3467 (PdxO), TF
PdxW (Actinobacteria)	PdxW - Streptomycetaceae	Streptomyces scabiei 87.22	SCAB_76461	COG3467 (PdxO), TF
Detailed contents of recosntructed regulons is available in the RegPrecise database (http://regprecise.lbl.gov/RegPrecise/collection_tffam.jsp?tffamily_id=83).				

[1] Orthologous groups of regulators are listed according to the order of their branches on the MocR-TFs phylogenetic tree. Distinct annotated regulogs in each orthologous group are separated by horizontal lines.

[2] Multiple paralogs of a given gene in a regulon are highlighted with blue font and denoted as "* number of copies".

Table S2. Composition of 86 orthologous groups of MocR-TFs with reconstructed regulons.

Name of MocR-TF orthologous group [1]	Distribution of regulogs and regulons in the genomic collections [2].																																											Number of TFs	Number of reguloGs	Controlled Metabolic pathway or Biological Process [3]
	Caulobacterales (4)	Rhizobiales (15)	Rhodobacterales (15)	Rhodospirillales (9)	Sphingomonadales (7)	Alcaligenaceae (3)	Burkholderia (8)	Comamonadaceae (11)	Ralstonia (6)	Other various (12)	Alteromonadales (9)	Enterobacteriales (12)	Moraxellaceae (4)	Oceanospirillales/ Alteromonadales (12)	Pasteurellales (9)	Pseudomonadaceae (8)	Psychromonadaceae/ Aeromonadales (6)	Shewanellaceae (16)	Vibrionales (10)	Xanthomonadales (4)	Desulfovibrionales (10)	Desulfuromonadales (9)	Bacillales (11)	Enterococcaceae (2)	Lactobacillaceae (15)	Listeriaceae (4)	Staphylococcaceae (7)	Streptococcaceae (15)	Clostridia-1 (8)	Clostridia-2 (3)	Clostridia-3 (14)	Thermoanaero-bacterales (7)	Corynebacteriaceae (8)	Frankin./Propionibac. /Pseudonocard. (9)	Micrococcineae (14)	Mycobacteriaceae (9)	Nocardiaceae (4)	Streptomycetaceae (4)	Bacteroidaceae (11)	Flavobacteria (19)	Sphingobacteria (8)	Deinococcus-Thermus (5)	Cyanobacteria (14)			
Sbal_3352	0	0	0	0	0	0	0	0	0	0	0	1	0	0	0	0	0	3	0	0	0	0	0	0	0	0	0	0	0	0	0	0	0	0	0	0	0	0	0	0	0	0	0	4	2	Unknown metabolism
AHA_3778	0	0	0	0	0	0	0	0	0	0	0	1	0	0	0	0	2	0	0	0	0	0	0	0	0	0	0	0	0	0	0	0	0	0	0	0	0	0	0	0	0	0	0	3	2	Unknown metabolism
Spro_2017	0	0	0	0	0	0	0	0	0	0	0	1	0	0	0	0	0	0	0	0	0	0	0	0	0	0	0	0	0	0	0	0	0	0	0	0	0	0	0	0	0	0	0	1	1	Unknown metabolism
PdxR (Proteobacteria)	0	2	0	1	0	2	0	2	0	1	0	1	0	0	0	2	0	0	0	0	0	0	0	0	0	0	0	0	0	0	0	0	0	0	0	0	0	0	0	0	0	0	0	11	7	PLP biosynthesis and salvage
PdxW (Proteobacteria)	0	0	0	0	0	3	10	2	2	1	0	2	0	0	0	0	0	0	0	0	0	0	0	0	0	0	0	0	0	0	0	0	0	0	0	0	0	0	0	0	0	0	0	20	6	PLP metabolism
PdxW2 (Oceanospirillales)	0	0	0	0	0	0	0	0	0	0	0	0	0	1	0	0	0	0	0	0	0	0	0	0	0	0	0	0	0	0	0	0	0	0	0	0	0	0	0	0	0	0	0	1	1	PLP metabolism
CV4096	0	0	0	0	0	0	0	0	0	1	0	0	0	0	0	0	0	0	0	0	0	0	0	0	0	0	0	0	0	0	0	0	0	0	0	0	0	0	0	0	0	0	0	1	1	Unknown metabolism
GabR (Proteobacteria)	0	0	0	0	0	7	16	3	5	1	0	5	2	0	0	0	1	0	0	0	0	0	0	0	0	0	0	0	0	0	0	0	0	0	0	0	0	0	0	0	0	0	0	40	8	GABA metabolism
PdxW2 (Proteobacteria)	0	0	0	0	0	0	6	0	0	0	0	0	1	0	0	6	0	0	0	0	0	0	0	0	0	0	0	0	0	0	0	0	0	0	0	0	0	0	0	0	0	0	0	13	3	PLP metabolism
PdxW (Gammaproteobacteria)	0	0	0	0	0	0	0	0	0	0	0	0	0	2	0	0	0	0	6	0	0	0	0	0	0	0	0	0	0	0	0	0	0	0	0	0	0	0	0	0	0	0	0	8	2	PLP metabolism
PdxQ (Bacillales)	0	0	0	0	0	0	0	0	0	0	0	0	0	0	0	0	0	0	0	0	0	0	2	0	0	0	0	0	0	0	0	0	0	0	0	0	0	0	0	0	0	0	0	2	1	PLP metabolism, Metabolite transport
PdxW (Desulfuromonadales)	0	0	0	0	0	0	0	0	0	0	0	0	0	0	0	0	0	0	0	0	0	1	0	0	0	0	0	0	0	0	0	0	0	0	0	0	0	0	0	0	0	0	0	1	1	PLP metabolism
PdxR (Bacilli)	0	0	0	0	0	0	0	0	0	0	0	0	0	0	3	0	0	0	0	0	0	0	2	0	0	0	6	0	0	0	0	0	0	0	0	0	0	0	0	0	0	0	0	11	3	PLP biosynthesis and salvage
YhdI/YdeL	0	0	0	0	0	0	0	0	0	0	0	0	0	0	0	0	0	0	0	0	0	0	6	0	0	0	1	0	0	0	0	0	0	0	0	0	0	0	0	0	0	0	0	7	2	Unknown metabolism, Metabolite transport
PdxR (Clostridia)	0	0	0	0	0	0	0	0	0	0	0	0	0	0	0	0	0	0	0	0	0	0	0	0	0	0	0	0	0	0	15	0	0	0	0	0	0	0	0	0	0	0	0	15	1	PLP biosynthesis and salvage
GabR (Bacilli)	0	0	0	0	0	0	0	0	0	0	0	0	0	0	0	0	0	0	0	0	0	0	4	0	0	0	1	0	0	0	0	0	0	0	0	0	0	0	0	0	0	0	0	5	2	GABA metabolism
PdxQ (Deltaproteobacteria)	0	0	0	0	0	0	0	0	0	0	0	0	0	0	0	0	0	0	0	0	2	1	0	0	0	0	0	0	2	0	0	0	0	0	0	0	0	0	0	0	0	0	0	5	3	PLP metabolism, Metabolite transport
DVU2086	0	0	0	0	0	0	0	0	0	0	0	0	0	0	0	0	0	0	0	0	1	0	1	0	0	0	0	0	0	1	0	0	0	0	0	0	0	0	0	0	0	0	0	3	3	Unknown metabolism, Metabolite transport
PdxW (Bacillales)	0	0	0	0	0	0	0	0	0	0	0	0	0	0	0	0	0	0	0	0	0	0	1	0	0	0	0	0	0	0	0	0	0	0	0	0	0	0	0	0	0	0	0	1	1	PLP metabolism
PdxQ (Proteobacteria)	0	1	1	1	0	1	0	2	2	0	0	0	0	1	0	0	0	0	0	0	0	0	0	0	0	0	0	0	0	0	0	0	0	0	0	0	0	0	0	0	0	0	0	9	7	PLP metabolism, Metabolite transport
TauR	0	1	10	0	0	0	3	5	3	0	0	1	0	2	0	0	1	0	0	0	0	0	0	0	0	0	0	0	0	0	0	0	0	0	0	0	2	0	0	0	0	0	0	28	9	Taurine metabolism
TauR2	0	1	2	0	0	0	0	0	0	0	0	0	0	0	0	0	0	0	0	0	0	0	0	0	0	0	0	0	0	0	0	0	0	0	0	0	0	0	0	0	0	0	0	3	2	Taurine metabolism
PdxW (Alteromonadales)	0	0	0	0	0	0	0	0	0	0	2	0	0	0	0	0	0	1	0	0	0	0	0	0	0	0	0	0	0	0	0	0	0	0	0	0	0	0	0	0	0	0	0	3	2	PLP metabolism
Cpin_2755	0	0	0	0	0	0	0	0	0	0	0	0	0	0	0	0	0	0	0	0	0	0	0	0	0	0	0	0	0	0	0	0	0	0	0	0	0	0	0	0	3	0	0	3	1	Unknown metabolism, RedOx
Cpin_4623	0	0	0	0	0	0	0	0	0	0	0	0	0	0	0	0	0	0	0	0	0	0	0	0	0	0	0	0	0	0	0	0	0	0	0	0	0	0	0	5	3	0	0	8	2	Unknown metabolism, Metabolite transport
PdxW (Sphingobacteria)	0	0	0	0	0	0	0	0	0	0	0	0	0	0	0	0	0	0	0	0	0	0	0	0	0	0	0	0	0	0	0	0	0	0	0	0	0	0	0	0	2	0	0	2	1	PLP metabolism
PP2642	0	1	0	0	0	0	1	0	0	0	0	0	1	1	0	1	0	0	0	0	0	0	0	0	0	0	0	0	0	0	0	0	0	0	0	0	0	0	0	0	0	0	0	5	5	Unknown metabolism, RedOx
PdxR2 (Alphaproteobacteria)	0	1	2	0	1	0	0	0	0	0	0	0	0	0	0	0	0	0	0	0	0	0	0	0	0	0	0	0	0	0	0	0	0	0	0	0	0	0	0	0	0	0	0	4	3	PLP biosynthesis and salvage
PdxQ (Actinobacteria)	0	0	0	0	0	0	0	0	0	0	0	0	0	0	0	0	0	0	0	0	0	0	0	0	0	0	0	0	0	0	0	0	0	1	4	2	1	4	0	0	0	0	0	12	5	PLP metabolism, Metabolite transport
PBPRB0322	0	0	0	0	0	0	0	0	0	0	1	0	0	1	0	0	0	12	2	0	0	0	0	0	0	0	0	0	0	0	0	0	0	0	0	0	0	0	0	0	0	0	0	16	4	Metabolite transport
PBPRA0298	0	0	0	0	0	0	0	0	0	0	0	0	0	0	0	0	2	0	3	0	0	0	0	0	0	0	0	0	0	0	0	0	0	0	0	0	0	0	0	0	0	0	0	5	2	Metabolite transport
YrdX2	0	3	0	0	0	0	0	0	0	0	0	0	0	0	0	0	0	0	0	0	0	0	0	0	0	0	0	0	0	0	0	0	0	0	0	0	0	0	0	0	0	0	0	3	1	Unknown metabolism, RedOx
YrdX	0	3	0	0	0	0	3	3	1	0	0	0	0	0	0	0	0	0	0	0	0	0	0	0	0	0	0	0	0	0	0	0	0	0	0	0	0	0	0	0	0	0	0	10	4	Unknown metabolism, RedOx
YrdX3	3	0	0	2	1	0	0	0	5	0	0	0	0	0	0	0	0	0	0	2	0	0	0	0	0	0	0	0	0	0	0	0	0	0	0	0	0	0	0	0	0	0	0	13	6	Unknown metabolism, RedOx
PdxQ2 (Proteobacteria)	2	1	0	4	2	0	0	1	0	0	0	0	0	0	0	0	0	0	0	0	0	0	0	0	0	0	0	0	0	0	0	0	0	0	0	0	0	0	0	0	0	0	0	10	5	PLP metabolism, Metabolite transport
EF0117	0	0	0	0	0	0	0	0	0	0	0	0	0	0	0	0	0	0	0	0	0	0	0	2	2	0	0	1	2	2	1	0	0	0	0	0	0	0	0	0	0	0	0	10	6	Unknown metabolism, Metabolite transport
YdeF	0	0	0	0	0	0	0	0	0	0	0	0	0	0	0	0	0	0	0	0	0	0	2	0	0	0	0	0	0	0	0	0	0	0	0	0	0	0	0	0	0	0	0	2	1	Unknown metabolism, Metabolite transport
mll0059	0	6	0	0	0	0	0	0	0	0	0	0	0	0	0	0	0	0	0	0	0	0	0	0	0	0	0	0	0	0	0	0	0	0	0	0	0	0	0	0	0	0	0	6	1	Unknown metabolism
PdxQ3 (Proteobacteria)	0	3	2	0	0	0	3	2	1	0	0	0	0	1	0	3	0	0	0	0	0	0	0	0	0	0	0	0	0	0	0	0	0	0	0	1	1	4	0	0	0	0	0	21	10	PLP metabolism, Metabolite transport
EutR	0	6	10	0	0	0	4	1	0	0	0	0	0	1	0	1	0	0	0	0	0	0	0	0	0	0	0	0	0	0	0	0	0	0	0	0	0	0	0	0	0	0	0	23	6	Ectoine metabolism
Tola_2572	0	0	0	0	0	0	0	0	0	0	0	0	0	0	0	3	3	0	2	0	0	0	0	0	0	0	0	0	0	0	0	0	0	0	0	0	0	0	0	0	0	0	0	8	3	Unknown metabolism, RedOx
PA2100	0	0	0	0	0	0	0	0	0	0	0	0	0	1	0	2	0	0	0	0	0	0	0	0	0	0	0	0	0	0	0	0	0	0	0	0	0	0	0	0	0	0	0	3	2	Unknown metabolism, Metabolite transport
PdxR3 (Actinobacteria)	1	0	1	0	0	0	0	1	0	0	0	3	0	0	0	0	0	0	0	0	0	0	0	0	0	0	0	0	0	0	0	0	0	5	7	6	6	6	0	0	0	0	0	36	9	PLP biosynthesis/salvage; Metabolite transport
Daci_0288	0	0	2	0	0	1	0	1	0	0	0	0	0	0	0	0	0	0	0	0	0	0	0	0	0	0	0	0	0	0	0	0	0	0	0	0	0	0	0	0	0	0	0	4	3	Metabolite transport, RedOx
GabR2 (Proteobacteria)	0	0	0	0	0	0	0	4	0	0	0	0	0	0	0	2	0	0	0	0	0	0	0	0	0	0	0	0	0	0	0	0	0	0	0	0	0	0	0	0	0	0	0	6	2	GABA metabolism
OapR	0	1	0	1	0	3	2	0	6	0	0	0	0	0	0	0	0	0	0	0	0	0	0	0	0	0	0	0	0	0	0	0	0	0	0	0	0	0	0	0	0	0	0	13	5	GABA metabolism
RALTA_A0216	0	0	0	0	0	0	2	0	5	0	0	0	0	0	0	0	0	0	0	0	0	0	0	0	0	0	0	0	0	0	0	0	0	0	0	0	0	0	0	0	0	0	0	7	2	Unknown metabolism, Metabolite transport
PdxW (Methylophilaceae)	0	0	0	0	0	0	0	0	0	1	0	0	0	0	0	0	0	0	0	0	0	0	0	0	0	0	0	0	0	0	0	0	0	0	0	0	0	0	0	0	0	0	0	1	1	PLP metabolism
ACIAD1897	0	11	1	0	0	0	4	2	0	0	0	0	2	1	0	2	0	0	0	1	0	0	0	0	0	0	0	0	0	0	0	0	0	0	0	0	0	0	0	0	0	0	0	24	8	Metabolite transport
Daci_0595	0	0	0	0	0	3	8	11	6	0	0	0	0	0	0	0	0	0	0	0	0	0	0	0	0	0	0	0	0	0	0	0	0	0	0	0	0	0	0	0	0	0	0	28	4	Unknown metabolism, Metabolite transport
DVU0030	0	0	0	0	0	0	0	0	0	0	0	0	0	0	0	0	0	0	0	0	7	0	0	0	0	0	0	0	0	0	0	0	0	0	0	0	0	0	0	0	0	0	0	7	1	Unknown metabolism, Metabolite transport
VEA_000881	0	0	0	0	0	0	0	1	0	0	3	0	0	2	0	0	0	0	6	0	0	0	0	0	0	0	0	0	0	0	0	0	0	0	0	0	0	0	0	0	1	0	0	13	5	Metabolite transport, RedOx
YE4144	0	0	0	0	0	0	0	0	0	2	0	2	1	0	0	0	0	1	0	1	0	0	0	0	0	0	0	0	0	0	0	0	0	0	0	0	0	0	0	0	0	0	0	7	5	Unknown metabolism, Metabolite transport
H16_A0970	0	0	0	0	0	3	4	6	6	2	0	0	0	0	0	0	0	0	0	0	0	0	0	0	0	0	0	0	0	0	0	0	0	0	0	0	0	0	0	0	0	0	0	21	5	Unknown metabolism, RedOx
YjiR2	0	0	0	0	0	0	0	0	0	0	0	0	0	5	0	0	0	0	0	0	0	0	0	0	0	0	0	0	0	0	0	0	0	0	0	0	0	0	0	0	0	0	0	5	1	Unknown metabolism, Metabolite transport
YjiR	0	0	0	0	0	0	0	0	0	0	0	6	0	5	0	6	0	0	0	0	0	0	0	0	0	0	0	0	0	0	0	0	0	0	0	0	0	0	0	0	0	0	0	17	3	Unknown metabolism, Metabolite transport
Pcar_1599	0	0	0	0	0	0	0	0	0	0	0	0	0	0	0	0	0	0	0	0	0	5	0	0	0	0	0	0	0	0	0	0	0	0	0	0	0	0	0	0	0	0	0	5	1	Unknown metabolism
Vapar_1337	0	0	0	0	0	0	1	1	0	0	0	0	0	0	0	0	0	0	0	0	0	0	0	0	0	0	0	0	0	0	0	0	0	0	0	0	0	0	0	0	0	0	0	2	2	Unknown metabolism
Cpin_4810	0	0	0	0	0	0	0	0	0	0	0	0	0	0	0	0	0	0	0	0	0	0	0	0	0	0	0	0	0	0	0	0	0	0	0	0	0	0	1	2	6	0	0	9	3	Unknown metabolism, Metabolite transport
sll5086	0	0	0	0	0	0	0	0	0	0	0	0	0	0	0	0	0	0	0	0	0	0	0	0	0	0	0	0	0	0	0	0	0	0	0	0	0	0	0	0	0	0	2	2	1	Unknown metabolism
YdcR	0	0	0	0	0	1	11	7	3	7	0	11	2	2	0	6	2	5	8	0	0	0	0	0	0	0	0	0	0	0	0	0	0	0	0	0	0	0	0	0	0	0	0	65	12	Metabolite transport, RedOx
H16_B1468	0	0	0	0	0	0	8	0	4	2	0	0	0	0	0	0	0	0	0	0	0	0	0	0	0	0	0	0	0	0	0	0	0	0	0	0	0	0	0	0	0	0	0	14	3	Metabolite transport, RedOx
Mmwyl1_0910	0	0	0	0	0	0	0	0	0	0	1	1	2	2	0	0	1	0	3	2	0	0	0	0	0	0	0	0	0	0	0	0	0	0	0	0	0	0	0	0	0	0	0	12	7	Unknown metabolism, Metabolite transport
YdfD/YisV	0	0	0	0	0	0	0	0	0	0	0	0	0	0	0	0	0	0	0	0	0	0	7	0	1	0	4	0	2	0	0	0	0	0	0	0	0	0	0	0	0	0	0	14	4	Unknown metabolism, Metabolite transport
PdxR (Lactobacillales)	0	0	0	0	0	0	0	0	0	0	0	0	0	0	0	0	0	0	0	0	0	0	0	2	2	4	0	0	0	0	0	0	0	0	0	0	0	0	0	0	0	0	0	8	3	PLP biosynthesis and salvage
BC3039	0	0	0	0	0	0	0	0	0	0	0	0	0	0	0	0	0	0	0	0	0	0	1	0	0	0	0	0	0	0	0	0	0	0	0	0	0	0	0	0	0	0	0	1	1	Metabolite transport
YcxD	0	0	0	0	0	0	0	0	0	0	0	0	0	0	0	0	0	0	0	0	0	0	5	0	0	0	0	0	2	0	0	0	0	0	0	0	0	0	0	0	0	0	0	7	2	Unknown metabolism, Metabolite transport
PdxR (Streptococcaceae)	0	0	0	0	0	0	0	0	0	0	0	0	0	0	0	0	0	0	0	0	0	0	0	0	0	0	0	7	0	0	0	0	0	0	0	0	0	0	0	0	0	0	0	7	1	PLP biosynthesis and salvage
PdxR (Thermoanaerobacterales)	0	0	0	0	0	0	0	0	0	0	0	0	0	0	0	0	0	0	0	0	0	0	0	0	0	0	0	0	0	0	0	2	0	0	0	0	0	0	0	0	0	0	0	2	1	PLP biosynthesis and salvage
Spro_3347	0	0	0	0	0	0	0	0	0	0	0	1	0	0	0	0	0	0	0	0	2	0	0	0	0	0	0	0	0	0	0	0	0	0	0	0	0	0	0	0	0	0	0	3	2	Metabolite transport
PdxR2 (Actinobacteria)	0	0	0	0	0	0	0	0	0	0	0	0	0	0	0	0	0	0	0	0	0	0	0	0	0	0	0	0	0	0	0	0	0	5	1	1	4	0	0	0	0	1	0	12	5	PLP biosynthesis and salvage
PdxR (Actinobacteria)	0	0	0	0	0	0	0	0	0	0	0	0	0	0	0	0	0	0	0	0	0	0	0	0	0	0	0	0	0	0	0	0	3	1	3	0	0	0	0	0	0	0	0	7	3	PLP biosynthesis and salvage
SGR_2706	0	0	0	0	0	0	0	0	0	0	0	0	0	0	0	0	0	0	0	0	0	0	0	0	0	0	0	0	0	0	0	0	0	3	1	8	4	11	0	0	0	0	0	27	5	Metabolite transport, RedOx
Bcav_0044	0	0	0	0	0	0	0	0	0	0	0	0	0	0	0	0	0	0	0	0	0	0	0	0	0	0	0	0	0	0	0	0	0	0	1	0	0	0	0	0	0	0	0	1	1	Unknown metabolism, RedOx
PA0268	0	0	0	0	0	0	6	0	5	0	0	0	0	0	0	3	0	0	0	0	0	0	0	0	0	0	0	0	0	0	0	0	0	0	0	0	0	0	0	0	0	0	0	14	3	Metabolite transport, RedOx
Spro_3719	0	0	0	0	0	0	4	2	2	1	0	7	0	1	0	1	0	1	0	0	0	0	0	0	0	0	0	0	0	0	0	0	0	0	0	0	0	0	0	0	0	0	0	19	8	Unknown metabolism, RedOx
blr6977	0	1	0	0	1	1	4	1	2	2	0	0	0	0	0	0	0	0	0	0	0	0	0	0	0	0	0	0	0	0	0	0	0	0	0	0	0	0	0	0	0	0	0	12	7	Unknown metabolism, RedOx
SPOA0379	0	0	2	0	0	0	0	1	0	0	0	0	0	0	0	0	0	0	0	0	0	0	0	0	0	0	0	0	0	0	0	0	0	0	0	0	0	0	0	0	0	0	0	3	2	Unknown metabolism, Metabolite transport
Veis_0044	0	2	0	0	0	0	0	1	0	0	0	0	0	0	0	0	0	0	0	0	0	0	0	0	0	0	0	0	0	0	0	0	0	0	0	0	0	0	0	0	0	0	0	3	2	Unknown metabolism, Metabolite transport
PdxW2 (Comamonadaceae)	0	0	0	0	0	0	0	1	0	0	0	0	0	0	0	0	0	0	0	0	0	0	0	0	0	0	0	0	0	0	0	0	0	0	0	0	0	0	0	0	0	0	0	1	1	PLP metabolism
RHA1_ro08093	0	0	0	0	0	0	0	0	0	0	0	0	0	0	0	0	0	0	0	0	0	0	0	0	0	0	0	0	0	0	0	0	0	0	0	0	1	0	0	0	0	0	0	1	1	Unknown metabolism, RedOx
PdxW (Rhodospirillales)	0	0	0	2	0	0	0	0	0	0	0	0	0	0	0	0	0	0	0	0	0	0	0	0	0	0	0	0	0	0	0	0	0	0	0	0	0	0	0	0	0	0	0	2	1	PLP metabolism
PdxW (Rhodobacterales)	0	0	1	0	0	0	0	0	0	0	0	0	0	0	0	0	0	0	0	0	0	0	0	0	0	0	0	0	0	0	0	0	0	0	0	0	0	0	0	0	0	0	0	1	1	PLP metabolism
PdxW (Alphaproteobacteria)	1	4	0	0	0	0	0	0	0	0	0	0	0	0	0	0	0	0	0	0	0	0	0	0	0	0	0	0	0	0	0	0	0	0	0	0	0	0	0	0	0	0	0	5	2	PLP metabolism
PdxR2 (Proteobacteria)	0	0	0	0	0	2	0	0	1	0	0	5	0	0	0	0	0	0	0	1	0	0	0	0	0	0	0	0	0	0	0	0	0	0	2	0	0	0	0	0	0	0	0	11	5	PLP biosynthesis and salvage
PdxW (Actinobacteria)	0	0	0	0	0	0	0	0	0	0	0	0	0	0	0	0	0	0	0	0	0	0	0	0	0	0	0	0	0	0	0	0	0	0	0	2	0	5	0	0	0	0	0	7	2	PLP metabolism
**(1) TFs**	7	48	34	11	5	27	100	61	59	21	7	48	11	29	3	38	12	23	30	7	12	7	31	4	5	4	12	8	8	3	16	2	3	15	19	20	19	30	1	7	15	1	2	825	-	**Total number of reguloNs = 761 (825 TFs)**
**(2) ReguloGs**	4	17	11	6	4	11	19	23	17	11	4	15	7	16	1	13	7	6	7	5	4	3	10	2	3	1	4	2	4	2	2	1	1	5	7	6	7	5	1	2	5	1	1	-	284	**Total number of regulogs = 284**

[1] Orthologous groups containing 2 or more regulogs are highlighted by yellow; singleton groups are not colored.

[2] Genomic collections are from the RegPrecise database. Number of studied genomes in each collection is given in parenthesis.

[3] Cells are colored according to the type of the metabolic process (see Table S3).

Each non-empty cell in the table represents a reconstructed reguloG and indicates a number of reconstructed regulons for MocR-TFs from a specific orthologous group in a specific taxonomic collection.

Table S3. Functional classification of genes from the reconstructed MocR-TF regulons.

Protein ID (COG, Pfam, name of the gene)	Function	Regulated gene count
**Ectoine metabolic and transport genes (members of the EutR-regulons)**		
COG3473, PF01177, eutA	Putative PLP-dependent decarboxylase/isomerase	8
COG1171 (IlvA), PF00291, eutB	PLP-dependent threonine dehydratase (EC 4.3.1.19)	15
COG2423, PF02423, eutC	Putative ornithine cyclodeaminase (EC 4.3.1.12)	15
COG6 (PepP), eutD/doeA	Putative ectoine hydrolase (EC 3.4.13.9)	17
COG3608, PF04952, eutE/doeB	Putative N-α-acetyl-L-2,4-diaminobutyrate deacetylase	14
COG1012 (PutA), PF00171, doeC	Putative aspartate-semialdehyde dehydrogenase	9
COG161 (BioA), PF00202, doeD	Putative PLP-dependent L-2,4-diaminobutyrate transaminase	9
COG1522 (Lrp), PF01037, doeX	Transcriptional regulator of ectoine metabolism, AsnC/Lrp family	12
COG1126 (GlnQ), PF00005, ehuA	Ectoine/hydroxyectoine ABC transporter, ATP-binding protein	11
COG834 (HisJ), PF00497, ehuB	Ectoine/hydroxyectoine ABC transporter, periplasmic binding protein	11
COG765 (HisM), PF00528, ehuC	Ectoine/hydroxyectoine ABC transporter, permease protein	11
COG765 (HisM), PF00528, ehuD	Ectoine/hydroxyectoine ABC transporter, permease protein	11
COG1638 (DctP), PF03480, teaA	Ectoine/hydroxyectoine TRAP transporter, periplasmic binding component	2
COG3090 (DctQ), PF04290, teaB	Ectoine/hydroxyectoine TRAP transporter, small permease component	2
COG1593 (DctM), PF06808, teaC	Ectoine/hydroxyectoine TRAP transporter, large permease component	2
COG589 (UspA), PF00582	Universal stress protein	2
COG5457, PF06568	Uncharacterized conserved small protein, yjjS-like	5
COG604 (Qor)	Quinone oxidoreductase (EC 1.6.5.5)/alcohol dehydrogenase	3
**GABA/putrescine metabolic and transport genes (members of the GabR-, GabR2- and OapR-regulons)**		
COG160 (GabT), PF00202, gabT	PLP-dependent γ-aminobutyrate:2-oxoglutarate aminotransferase (EC 2.6.1.19)	39
COG161 (BioA), PF00202, oapT	PLP-dependent ω-amino acid/γ-aminobutyrate:pyruvate aminotransferase (EC 2.6.1.18)	13
COG161 (BioA), PF00202, gatP	PLP-dependent γ-aminobutyrate transaminase (EC 2.6.1.96)	2
COG1012 (PutA), PF00171, gabD	NADP-dependent succinate-semialdehyde dehydrogenase (EC 1.2.1.16)	31
COG1012 (PutA), PF00171, oapD	Predicted NAD-dependent succinate-semialdehyde dehydrogenase (EC 1.2.1.16)	9
COG1113 (AnsP)/COG531 (PotE), PF00324, gabP	γ-aminobutyrate permease	6
COG3842 (PotA), PF00005, gbtA	Predicted γ-aminobutyrate ABC transporter, ATP-binding protein	14
COG1176 (PotB), PF00528, gbtB	Predicted γ-aminobutyrate ABC transporter, permease protein	17
COG1177 (PotC), PF00528, gbtC	Predicted γ-aminobutyrate ABC transporter, permease protein	16
COG687 (PotD), PF01547, gbtD	Predicted γ-aminobutyrate ABC transporter, periplasmic binding protein	17
COG3842 (PotA), PF00005, puuL	Predicted putrescine ABC transporter, ATP-binding protein	2
COG1176 (PotB), PF00528, puuM	Predicted putrescine ABC transporter, permease protein	2
COG1177 (PotC), PF00528, puuN	Predicted putrescine ABC transporter, permease protein	2
COG687 (PotD), PF01547, puuO	Predicted putrescine ABC transporter, periplasmic binding protein	2
COG747 (DdpA), PF00496, dppA	Predicted dipeptide ABC transporter, periplasmic component	5
COG601 (DppB), PF00528, dppB	Predicted dipeptide ABC transporter, permease component	5
COG1173 (DppC), PF00528, dppC	Predicted dipeptide ABC transporter, permease component	5
COG1123 (DppD), PF00005, dppD	Predicted dipeptide ABC transporter, ATP-binding component	5
COG174 (GlnA), PF00120, puuA	γ-glutamyl-putrescine synthetase (EC 6.3.1.11)	2
COG2071, puuD	γ-glutamyl-γ-aminobutyrate hydrolase (EC 3.5.1.94)	2
COG10 (SpeB), PF00491, speB	Agmatinase, arginase family (EC 3.5.3.11)	3
COG624 (ArgE), argE	Acetylornithine deacetylase (EC 3.5.1.16)	3
COG2334, PF01636	Putative aminoglycoside phosphotransferase/homoserine kinase type II	1
**Taurine metabolic and transport genes (members of the TauR- and TauR2-regulons)**		
COG4521 (TauA), PF04069, tauA	Taurine ABC transporter, periplasmic binding protein	11
COG4525 (TauB)/COG1116 (TauB), PF00005, tauB	Taurine ABC transporter, ATP-binding protein	13
COG600 (TauC), PF00528, tauC	Taurine ABC transporter, permease protein	13
COG1638 (DctP), PF03480, tauK	Taurine TRAP transporter, periplasmic binding component	3
COG3090 (DctQ), PF04290, tauL	Taurine TRAP transporter, small permease component	3
COG1593 (DctM), PF06808, tauM	Taurine TRAP transporter, large permease component	3
COG2175 (TauD), PF02668, tauD	α-ketoglutarate-dependent taurine dioxygenase (EC 1.14.11.17)	2
COG3631/COG4319, PF07366, tauX	Taurine dehydrogenase (EC 1.4.2.-), small subunit	12
COG665 (DadA), PF01266, tauY	Taurine dehydrogenase (EC 1.4.2.-), large subunit	13
COG161 (BioA), PF00202, tpa	Taurine-pyruvate aminotransferase (EC 2.6.1.77)	11
COG161 (BioA), PF00202	PLP-dependent aminotransferase	1
COG28 (IlvB), xsc	Thiamin diphosphate (ThDP)-coupled sulfoacetaldehyde acetyltransferase (EC 2.3.3.15)	12
COG280 (Pta), PF01515	Phosphate acetyltransferase (EC 2.3.1.8)	11
COG1113 (AnsP), PF00324, gabP	γ-aminobutyrate/homotaurine permease	9
COG730, PF01925, tauE	Predicted sulfite transporter	8
COG2855, PF03601, tauZ	Sulfate exporter protein	5
COG243 (BisC), PF00384, bisC	4Fe-4S anaerobic molybdopterin oxidoreductase subunit	6
COG3302 (DmsC), PF04976, dmsC	DMSO reductase membrane anchor subunit	6
COG437 (HybA), PF00037, hybA	4Fe-4S ferredoxin, dehydrogenase subunit (EC 1.97.1.9)	6
COG686 (Ald), ald	Alanine dehydrogenase (EC 1.4.1.1)	2
COG282 (AckA), PF00871, ackA	Acetate kinase (EC 2.7.2.1)	3
COG2022 (ThiG), PF05690, thiG	Thiazole biosynthesis protein	2
COG1574, PF07969	Metal-dependent amidohydrolase	3
COG0523, PF02492	P47K protein (cobalamin biosynthesis protein/nitrile hydratase activator/zinc-transport protein)	3
COG1638 (DctP), PF03480	TRAP-type transport system, periplasmic binding component	2
COG3090 (DctQ), PF04290	TRAP-type transport system, small permease component	2
COG1593 (DctM), PF06808	TRAP-type transport system, large permease component	2

COG1126 (GlnQ), PF00005	Amino acid ABC transporter, ATP-binding protein	2
COG834 (HisJ), PF00497	Amino acid ABC transporter, periplasmic binding protein	2
COG765 (HisM), PF00528	Amino acid ABC transporter, permease protein	1
COG4160 (ArtM), PF00528	Amino acid ABC transporter, permease protein	2
COG4215 (ArtQ), PF00528	Amino acid ABC transporter, permease protein	1
COG1012 (PutA), PF00171	Putative NAD-dependent phosphonoacetaldehyde/ 3-sulfopropanal dehydrogenase (EC 1.2.1.-)	4
COG596 (MhpC), PF06441	Epoxide hydrolase domain protein (alpha/beta hydrolase superfamily)	2
COG747 (DdpA), PF00496, oppA	Predicted dipeptide ABC transporter, periplasmic binding component	3
COG601 (DppB), PF00528, oppB	Predicted dipeptide ABC transporter, permease component	3
COG1173 (DppC), PF00528, oppC	Predicted dipeptide ABC transporter, permease component	3
COG1123 (DppD), PF00005, oppD	Predicted dipeptide ABC transporter, ATP-binding component	3
COG444 (DppD), PF00005, oppF	Predicted dipeptide ABC transporter, ATP-binding component	3
COG665 (DadA), PF01266	FAD dependent oxidoreductase	1
COG456 (RimI), PF00583, rimI	GCN5-related N-acetyltransferase (EC 2.3.1.-)	1
COG214 (PdxS), PF01680, pdxS	Pyridoxine biosynthesis glutamine amidotransferase, synthase subunit (EC 2.4.2.-)	1
COG2128, PF02627	Alkylhydroperoxidase, AhpD family/carboxymuconolactone decarboxylase (EC 4.1.1.44)	1
**PLP metabolic and transport genes (members of the PdxR-, PdxW- and PdxQ-regulons)**		
COG214 (PdxS), PF01680, pdxS	Pyridoxine biosynthesis glutamine amidotransferase, synthase subunit (EC 2.4.2.-)	34
COG311 (PdxT), PF01174, pdxT	Pyridoxine biosynthesis glutamine amidotransferase, glutaminase subunit (EC 2.4.2.-)	32
COG2240 (PdxK), PF08543, pdxK	Pyridoxal/pyridoxine/pyridoxamine kinase (EC 2.7.1.35)	23
COG351 (ThiD), PF08543, pdxK2	Pyridoxal kinase, ThiD family (EC 2.7.1.35)	5
COG4720 (PdxU), PF07155, pdxU	Substrate-specific component of predicted pyridoxine ECF transporter	27
COG3467 (PdxO), PF01243, pdxO	Pyridoxamine 5'-phosphate oxidase, FMN-binding domain protein	47
COG2808 (PaiB), PF04299, pdxO2	Putative pyridoxamine 5'-phosphate oxidase, FMN-binding domain protein	30
PF01243, pdxO3	Putative pyridoxamine 5'-phosphate oxidase, FMN-binding domain protein	1
COG259 (PdxH), PF01243, pdxH	Pyridoxamine 5'-phosphate oxidase (EC 1.4.3.5)	1
pdxZ	Short cytoplasmic protein involved in pyridoxine metabolism	5
COG456 (RimI), PF00583, rimI	GCN5-related N-acetyltransferase (EC 2.3.1.-)	18
COG1670 (RimL), PF00583, rimL	GCN5-related N-acetyltransferase (EC 2.3.1.-)	28
COG3135 (BenE), PF03594	Putative pyridoxine transport protein	8
COG112 (GlyA), PF00464, glyA	PLP-dependent glycine/serine hydroxymethyltransferase (EC 2.1.2.1)	6
COG329 (DapA), PF00701, dapA	Dihydrodipicolinate synthase/N-acetylneuraminate lyase (EC 4.2.1.52)	4
COG4992 (ArgD), PF00202, argD	PLP-dependent ornithine/acetylornithine aminotransferase (EC 2.6.1.11)	4
COG518 (GuaA), PF00117, guaA	GMP synthase - Glutamine amidotransferase class I (EC 6.3.5.2)	8
COG436, PF00155	PLP-dependent aspartate/tyrosine/aromatic aminotransferase class I/classII	1
COG156 (BioF), PF00155, bioF	PLP-dependent aminotransferase class I/classII	1
COG325, PF01168	PLP-binding protein involved in PLP homeostasis	11
COG2258, PF03473	PLP-binding MOSC (molybdenum cofactor sulfurase, C-terminal) domain containing protein	2
COG384 (PhzF), PF02567, phzF	Predicted epimerase, phenazine biosynthesis protein homolog, PhzF family	8
COG1764 (OsmC), PF02566, osmC	Peroxiredoxin, regulator of disulfide bond formation, OsmC/Ohr family protein	1
COG235 (AraD), PF00596, araD	Ribulose-5-phosphate 4-epimerase and related class II epimerases and aldolases	1
COG346 (GloA), PF00903, gloA	Putative dioxygenase/lyase, related to lactoylglutathione lyase, glyoxalase family protein	2
COG520 (csdA), PF00266, csdA	PLP-dependent transferase class-V, cysteine desulfurase (EC 2.8.1.7)/selenocysteine lyase (EC	1
COG697 (RhaT), PF00892	Permease of the drug/metabolite transporter (DMT) superfamily	13
COG1280 (RhtB), PF01810	Lysine-type exporter protein (LysE/YggA family)	14
COG53 (MMT1), PF01545	Predicted Co/Zn/Cd cation transporter	1
COG2832, PF04304	Putative inner membrane protein	1
COG1359, PF03992	Antibiotic biosynthesis monooxygenase	1
PF03992	Antibiotic biosynthesis monooxygenase	1
COG5457, PF06568	Uncharacterized conserved small protein, yjjS-like	3
COG4475, PF04260, ywlG	Uncharacterized protein conserved in bacteria	4
COG3382, PF03483	Solo B3/4 domain (OB-fold DNA/RNA-binding) of Phe-aaRS-beta	2
COG596 (MhpC), PF06441	Epoxide hydrolase domain protein (alpha/beta hydrolase superfamily)	3
COG2814 (AraJ), PF07690	MFS sugar transporter/drug resistance transporter Bcr/CflA subfamily	3
COG4977, PF01965	ThiJ/PfpI domain protein, transcriptional regulator containing an amidase domain and an AraC-type	4
COG2050 (PaaI), PF03061	Protein, possibly involved in aromatic compounds catabolism, thioesterase superfamily	3
COG350 (Ada), PF01035	Methylated DNA-protein cysteine methyltransferase (2.1.1.63)	2
COG657 (Aes), PF07859	Esterase/lipase, alpha/beta hydrolase fold-3	1
COG584 (UgpQ), PF03009	Glycerophosphoryl diester phosphodiesterase (EC 3.1.4.46)	4
COG2270, PF11700	Permeases of the major facilitator superfamily	4
COG2364	Putative inner membrane protein regulating antibiotic production	31
COG217, PF01709	DNA-binding regulatory protein, YebC/PmpR family	1
PF10027	Conjugal transfer protein TrbL, integral membrane protein	1
COG730, PF01925	Predicted sulfite transporter	1
**Other regulons**		
**RedOx genes (cytochromes, ferredoxins and related genes, oxidoreductases and dehydrogenases)**		
COG1622 (CyoA), PF00116	Cytochrome c oxidase subunit II (EC 1.9.3.1)	5
COG843 (CyoB), PF00115	Cytochrome c oxidase, subunit I (EC 1.9.3.1)	5
COG1845 (CyoC), PF00510	Cytochrome c oxidase subunit III (EC 1.9.3.1)	9
COG3125 (CyoD), PF03626	Cytochrome c oxidase subunit IV (EC 1.9.3.1)	5
COG1271 (CydA), PF01654	Cytochrome bd-type quinol oxidase, subunit I (EC 1.10.3.-)	25
COG1294 (CydB/AppB), PF02322	Cytochrome bd-type quinol oxidase, subunit II (EC 1.10.3.-)	25
COG2124 (CypX), PF00067	Cytochrome P450 (EC 1.14.-.-)	1

COG3278 (CcoN), PF02433	Cytochrome c oxidase cbb3-type, subunit I (EC 1.9.3.1)	2
COG2863, PF00034	Cytochrome c553	6
COG3258, PF00034	Cytochrome c, class I	1
COG4117, PF00033	Thiosulfate reductase cytochrome b subunit (membrane anchoring protein)	1
COG348 (NapH)	4Fe-4S polyferredoxin, cytochrome c oxidase accessory protein	23
COG243 (BisC/FdhA)	4Fe-4S formate dehydrogenase subunit, molydopterin-binding (EC 1.2.1.2)	14
COG1526 (FdhD), PF02634	Formate dehydrogenase accessory protein	4
COG155 (CysI), PF01077	Nitrite/sulphite reductase (NADPH-binding) 4Fe-4S domain, ferrodoxin-like beta subunit (EC 1.8.1.2)	12
AdhA	Three-component membrane-bound alcohol dehydrogenase subunit	3
AdhB, PF00034	Three-component membrane-bound alcohol dehydrogenase, cytochrome c subunit	3
AdhS, PF11174	Three-component membrane-bound alcohol dehydrogenase, small gamma subunit	11
COG2303 (BetA/GadH1)	Gluconate 2-dehydrogenase, membrane-bound, flavoprotein subunit (EC 1.1.99.3)	1
COG2010 (CccA/GadH2), PF00034	Gluconate 2-dehydrogenase, membrane-bound, cytochrome c subunit (EC 1.1.99.3)	1
GadH3, Pfam13618	Gluconate 2-dehydrogenase, membrane-bound, small gamma subunit (EC 1.1.99.3)	1
COG2010 (CccA), PF00034	Membrane-bound cytochrome c subunit	5
COG3749	Putative sulfate/sulfite oxidoreductase	12
PF10617	Protein of unknown function (DUF2474), cytochrome bd ubiquinol oxidase associated protein	3
COG2896 (MoaA)	Molybdenum cofactor biosynthesis protein, iron-sulfur binding	2
COG303 (MoeA)	Molybdenum cofactor biosynthesis protein	3
COG2871 (NqrF)	Na^+-translocating NADH:ubiquinone oxidoreductase, ferrodoxin-like beta subunit (EC 1.6.5.-)	2
COG5349, PF06170	Conserved hypothetical protein in cyt c oxidase gene clusters	9
COG604 (Qor)	Quinone oxidoreductase (EC 1.6.5.5)/alcohol dehydrogenase	1
COG2084 (MmsB), PF03446	NAD-binding 3-hydroxyacid dehydrogenase (EC 1.1.1.-)	5
COG2041, PF00174	Oxidoreductase, molybdopterin-binding domain	1
COG4232, PF02683	Thioredoxin-like cytochrome c assembly protein	2
COG1225 (Bcp), PF00578	Alkyl hydroperoxide reductase, AhpC/TSA family, thioredoxin-like fold protein (EC 1.11.1.-)	2
COG2128, PF02627	Alkylhydroperoxidase, AhpD family/carboxymuconolactone decarboxylase (EC 4.1.1.44)	53
COG599, PF02627	Alkylhydroperoxidase-like protein, AhpD family	1
COG1917, PF07883	Putative dioxygenase with cupin 2, RmlC-type domain	19
PF07883	Putative dioxygenase with cupin 2, RmlC-type domain	2
COG1182 (AcpD), PF02525	Acyl carrier protein phosphodiesterase/FMN-dependent NADH-azoreductase (EC 3.1.4.14)	7
COG665 (DadA), PF01266	FAD-dependent glycine/D-amino acid oxidases (deaminating)	3
COG1028 (FabG), PF00106	Short-chain dehydrogenase	4
COG4221	Short-chain dehydrogenase/reductase of unknown specificity	1
COG1257 (HMG1/MvaA), PF00368	Hydroxymethylglutaryl-CoA reductase (EC 1.1.1.34)	4
COG2509	FAD-dependent oxidoreductase	4
COG1999	Thioredoxin-like copper chaperone SCO1/SenC, involved in biogenesis of respiratory and photosynthetic systems	1
COG1764 (OsmC), PF02566, osmC	Peroxiredoxin, regulator of disulfide bond formation, OsmC/Ohr family protein	2
COG1960 (CaiA)	Acyl-CoA dehydrogenase, short-chain specific (EC 1.3.99.-)	1
COG667 (Tas), PF00248	Putative NADP-dependent aldo/keto oxidoreductase (related to aryl-alcohol dehydrogenases)	1
COG673 (MviM), PF01408	NADH-dependent oxidoreductase domain protein, putative myo-inositol 2-dehydrogenase	2
COG2072 (TrkA), PF07992	FAD-dependent flavin-containing oxidoreductase	11
COG1893 (ApbA)	Ketopantoate reductase ApbA/PanE domain protein (EC 1.1.1.169)	1
COG506 (PutA), PF01619	Proline dehydrogenase (EC 1.5.99.8)	1
COG1012 (PutA), PF00171	NAD-dependent aldehyde dehydrogenase (EC 1.2.1.3)	1
COG1231, PF01593	Flavin-containing monoamine oxidase (EC 1.4.3.4)	1
PF05899	Putative dioxygenase with cupin 3, RmlC-type domain	2
PF03992	Antibiotic biosynthesis monooxygenase	3
PF10777, ylaC	Extracytoplasmic function sigma factor YlaC involved in resistance to oxidative stress	8
**PLP-dependent enzymes**		
COG436, PF00155	PLP-dependent aspartate/tyrosine/aromatic aminotransferase class I/classII	2
COG112 (GlyA), PF00464, glyA	PLP-dependent glycine/serine hydroxymethyltransferase (EC 2.1.2.1)	1
COG1167 (ARO8), PF00155, aspB	PLP-dependent aminotransferase class I/classII	26
COG1448 (TyrB), PF00155, tyrB	PLP-dependent aspartate/tyrosine/aromatic aminotransferase class I/classII (2.6.1.57)	1
COG76 (GadB), PF00282, gadB	PLP-dependent transferase glutamate decarboxylase	4
COG115 (IlvE), PF01063, ilvE	PLP-dependent branched-chain amino acid aminotransferase class IV (EC 2.6.1.42)	2
COG1171 (IlvA), PF00291, ilvA	PLP-dependent threonine dehydratase (4.3.1.19)	5
COG498 (ThrC), PF00291, thrC	PLP-dependent threonine synthase (EC 4.2.3.1)	1
**Transferases**		
COG456 (RimI), PF00583, rimI	GCN5-related N-acetyltransferase (EC 2.3.1.-)	11
COG1670 (RimL), PF00583, rimL	GCN5-related N-acetyltransferase (EC 2.3.1.-)	4
PF00583	GCN5-related N-acetyltransferase (EC 2.3.1.-)	8
SSF55729	GCN5-related N-acetyltransferase (EC 2.3.1.-)	2
COG2162 (NhoA), PF00797	Arylamine N-acetyltransferase (EC 2.3.1.118)	1
COG607 (PspE), PF00581, pspE	Rhodanese-related sulfurtransferase	4
COG463 (WcaA), PF00535	Glycosyltransferases family 2 involved in cell wall biogenesis	1
COG3153, PF00583	GCN5-related N-acetyltransferase	11
COG438 (RfaG), PF00534	Glycosyltransferase, group 1	9
COG625 (Gst)	Glutathione S-transferase (EC 2.5.1.18)	10
COG388, PF00795	Nitrilase/cyanide hydratase and apolipoprotein N-acyltransferase (EC 3.5.5.7)	11
**Epimerases/isomerases, mutases and racemases**		
COG384 (PhzF), PF02567, phzF	Predicted epimerase, phenazine biosynthesis protein homolog, PhzF family	29

COG235 (AraD), PF00596, araD	Ribulose-5-phosphate 4-epimerase and related class II epimerases and aldolases	3
COG1082 (IolE), PF01261	Sugar phosphate isomerase/epimerase/ myo-inosose-2 dehydratase (EC 4.2.1.44)	3
COG4948	Muconolactone isomerase (EC 5.3.3.4)	2
COG1087 (GalE)	NAD-dependent UDP-glucose 4-epimerase	2
COG451 (WcaG)	NAD-dependent nucleoside-diphosphate-sugar epimerases, NmrA family	2
COG702, PF05368	Predicted nucleoside-diphosphate-sugar epimerases, NmrA-like domain	3
COG2513 (PrpB)	PEP phosphonomutase related protein, putative carboxyvinyl-carboxyphosphonate phosphorylmutase (EC 2.7.8.23)	1
COG1794 (RacX), PF01177, racX	Aspartate racemase, aspartate/glutamate/hydantoin racemase family (EC 5.1.1.13)	3
**Transport genes**		
COG3135 (BenE), PF03594, benE	Putative benzoate or pyridoxine transport protein	2
COG2962 (RarD), PF00892	Permease of the drug/metabolite transporter (DMT) superfamily	12
COG697 (RhaT), PF00892	Permease of the drug/metabolite transporter (DMT) superfamily	79
COG5006 (RhtA), PF00892	Permease of the drug/metabolite transporter (DMT) superfamily	4
COG1280 (RhtB), PF01810	Lysine-type exporter protein (LysE/YggA family)	62
COG1279, PF01810	Lysine-type exporter protein (LysE/YggA family)	11
COG2814 (AraJ), PF07690	MFS sugar transporter/drug resistance transporter Bcr/CflA subfamily	7
COG1296 (AzlC), PF03591, azlC	Branched-chain amino acid permease, AzlC family protein (azaleucine resistance)	8
COG4392 (AzlD), PF05437, azlD	Branched-chain amino acid transport protein (azaleucine resistance)	7
COG4206 (BtuB)/ COG4771 (FepA), omp	TonB-dependent receptor domain protein, outer membrane cobalamin/ferrienterochelin receptor	7
COG823 (TolB)	Periplasmic component of the Tol biopolymer transport system	2
COG4663 (FcbT1), PF03480	TRAP-type mannitol/chloroaromatic compound transport system, periplasmic binding component	3
COG4664 (FcbT3), PF06808	TRAP-type mannitol/chloroaromatic compound transport system, large permease component	3
COG4665 (FcbT2), PF04290	TRAP-type mannitol/chloroaromatic compound transport system, small permease component	3
COG1129, PF00005	Sugar ABC transporter, ATP-binding protein	1
COG1172 (AraH), PF02653	Sugar ABC transporter, permease protein	1
COG1879 (RbsB), PF00532	Sugar ABC transporter, periplasmic binding component	1
COG2271 (UhpC), PF07690, ydeG	Sugar phosphate permease	1
COG3842 (PotA), PF00005	ABC-type spermidine/putrescine transport system, ATP-binding protein	2
COG1176 (PotB), PF00528	ABC-type spermidine/putrescine transport system, permease component	2
COG1177 (PotC), PF00528	ABC-type spermidine/putrescine transport system, permease component	2
COG687 (PotD), PF01547	ABC-type spermidine/putrescine transport system, periplasmic binding protein	4
COG3781, PF01062	Predicted membrane protein	6
COG730, PF01925	Predicted sulfite transporter	3
**Other genes**		
COG251 (TdcF), PF01042, tdcF	Putative enamine/imine deaminase, RidA (YjgF) family	15
COG2516, PF04055	Biotin synthase-related radical SAM domain protein	11
COG2144	AIR synthase-like protein, selenophosphate synthetase-related	11
Pfam13468	Riboflavin-specific deaminase/glyoxalase-like domain protein	14
PF02635	Putative GTP cyclohydrolase II/sulphur relay, DsrE/DsrF-like protein	11
PF11142	Protein of unknown function DUF2917, Cupin, RmlC-type	7
COG2872	Predicted metal-dependent hydrolases related to threonyl/alanyl-tRNA synthetase (EC 6.1.1.7)	6
COG329 (DapA), PF00701, dapA	Dihydrodipicolinate synthase/N-acetylneuraminate lyase (EC 4.2.1.52)	4
COG2423, PF02423	Ornithine cyclodeaminase (EC 4.3.1.12)	2
PF01966	Metal dependent phosphohydrolase (HD superfamily)	4
COG2316, PF01966	Metal dependent phosphohydrolase (HD superfamily)	1
COG596 (MhpC), PF06441, mhpC	Epoxide hydrolase domain protein (alpha/beta hydrolase superfamily)	1
COG1486 (CelF)	6-phospho-beta-glucosidase (EC 3.2.1.86), family 4 of glycosyl hydrolases	1
COG4977, PF01965	ThiJ/PfpI domain protein, transcriptional regulator containing an amidase domain and an AraC-type	2
COG3382, PF03483	Solo B3/4 domain (OB-fold DNA/RNA-binding) of Phe-aaRS-beta	2
COG1335 (PncA), PF00857	Amidases related to nicotinamidase (EC 3.5.1.-)	1
COG1573, PF03167	Uracil-DNA glycosylase	1
COG2120, PF02585	LmbE family protein, putative N-acetylglucosaminyl phosphatidylinositol deacetylase	1
COG2199, PF00990	Diguanylate cyclase with PAS/PAC sensor	1
COG2200 (Rtn), PF00563	Diguanylate phosphodiesterase	1
COG3239 (DesA), PF00487	Fatty acid desaturase	1
COG4943, PF00563	Diguanylate phosphodiesterase, predicted signal transduction protein containing sensor domain	1
COG502 (BioB), PF06968	Biotin synthase (EC 2.8.1.6)	1
COG714, PF07728	MoxR-like ATPase	1
COG1310	Predicted metal-dependent protease of the PAD1/JAB1 superfamily	1
COG5457, PF06568	Uncharacterized conserved small protein, yjjS-like	16
COG1357, PF00805	Pentapeptide repeat family protein	1
COG3189, PF04343	Protein of unknown function DUF488	1
COG3864, PF09967	Protein of unknown function DUF2201, metallopeptidase-related	1
COG5502, PF10025	Protein of unknown function DUF2267	1
PF07681	DoxX family protein	1
PF07609	Protein of unknown function DUF1572	1
		1826

Revised manuscript with tracking changes

Click here to access/download

Additional Material for Reviewer

MGen_Suvorova_Rodionov_revised_with_tracking.docx
